# Calcium Hydride Catalysts for Olefin Hydrofunctionalization: Ring‐Size Effect of Macrocyclic Ligands on Activity

**DOI:** 10.1002/chem.202004931

**Published:** 2021-01-18

**Authors:** Thomas Höllerhage, Danny Schuhknecht, Alisha Mistry, Thomas P. Spaniol, Yan Yang, Laurent Maron, Jun Okuda

**Affiliations:** ^1^ Institute of Inorganic Chemistry RWTH Aachen University Landoltweg 1 52056 Aachen Germany; ^2^ CNRS, INSA, UPS, UMR 5215, LPCNO Université de Toulouse 135 avenue de Rangueil 31077 Toulouse France

**Keywords:** calcium hydride, hydrogenation, hydrosilylation, kinetic analysis, macrocycles

## Abstract

The fifteen‐membered NNNNN macrocycle Me_5_PACP (Me_5_PACP=1,4,7,10,13‐pentamethyl‐1,4,7,10,13‐pentaazacyclopentadecane) stabilized the [CaH]^+^ fragment as a dimer with a distorted pentagonal bipyramidal coordination geometry at calcium. The hydride complex was prepared by protonolysis of calcium dibenzyl with the conjugate acid of Me_5_PACP followed by hydrogenolysis or treating with ^n^OctSiH_3_ of the intermediate calcium benzyl cation. The calcium hydride catalyzed the hydrogenation and hydrosilylation of unactivated olefins faster than the analogous calcium complex stabilized by the twelve‐membered NNNN macrocycle Me_4_TACD (Me_4_TACD=1,4,7,10‐tetramethyl‐1,4,7,10‐tetraazacyclododecane). Kinetic investigations indicate that higher catalytic efficiency for the Me_5_PACP stabilized calcium hydride is due to easier dissociation of the dimer in solution when compared to the Me_4_TACD analogue.

## Graphical abstract

**Macrocyclic ligands** recognize ion size, but are rarely used to influence metal‐centered catalytic activity. Calcium hydride cation [CaH]^+^ supported by the fifteen‐membered NNNNN macrocycle Me_5_PACP (Me_5_PACP=1,4,7,10,13‐pentamethyl‐1,4,7,10,13‐pentaazacyclopentadecane) catalyzes hydrogenation and hydrosilylation of unactivated olefins significantly faster than the analogous calcium catalyst containing the twelve‐membered NNNN macrocycle (see figure).

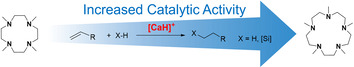

Calcium dihydride [CaH_2_]_*n*_ forms an ionic lattice with calcium ions of coordination number nine (PbCl_2_‐type, Δ*H*(lattice)=2410 kJ mol^−1^).^[1]^ Heating in vacuum activates its surface so that ethylene is hydrogenated using dihydrogen possibly by cationic calcium sites at the surface.^[2]^ Since the isolation of the first molecular calcium hydride complex [(BDI)Ca(thf)_2_(μ‐H)]_2_ (**A**; BDI=CH[C(CH_3_)NDipp]_2_, Dipp=2,6‐diisopropylphenyl), molecular calcium hydrides containing a variety of ancillary ligands^[3]^ have shown activity in olefin hydrogenation and in a number of related catalytic reactions, previously thought to be reserved for transition metal and lanthanide complexes (Figure 1).^[4]^ It can be assumed that the combination of electrophilic calcium center with a nucleophilic hydride ligand enables these catalytic reactions. In addition to colloidal [CaH_2_]_*n*_, calcium hydride clusters have been reported to catalyze olefin hydrogenation^[5]^ and a variety of hydride clusters of up to ten calcium atoms have been isolated.^[6]^ As an example of a mononuclear calcium hydride with a terminal Ca−H bond, [(Tp^Ad,*i*Pr^)Ca(H)(thp)] (Tp^Ad,*i*Pr^=hydrotris(3‐adamantyl‐5‐isopropyl‐pyrazolyl)borate) (**B**) has been reported more recently to catalyze olefin hydrogenation.^[3f]^ Such reactive mononuclear calcium hydrides can generally be conceived as the active species in catalytic reactions involving olefinic substrates.

**Figure 1:**
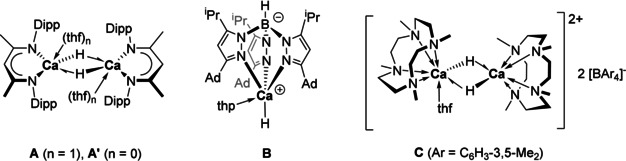
Examples of calcium hydride complexes for olefin hydrogenation.^[3a, 3f, 7c]^.

Compared to transition‐metal and lanthanide‐based systems, alkaline earth metal catalysts for hydrofunctionalization are still underdeveloped and understanding structure–activity/selectivity relationships in these systems appears crucial for their improvement. Previously we have introduced the NNNN macrocycle Me_4_TACD (Me_4_TACD=1,4,7,10‐tetramethyl‐1,4,7,10‐tetraazacyclododecane) derived from the twelve‐membered cyclen to support [CaH]^+^ as dinuclear hydride cations [(Me_4_TACD)_2_Ca_2_(μ‐H)_2_(thf)][BAr_4_]_2_ (**C**, Ar=C_6_H_3_‐3,5‐Me_2_) and shown their reactivity toward inactivated olefins.[^7b^, ^7c^] Evidently, this macrocycle is capable of kinetically stabilizing highly electrophilic fragments such as [CaH]^+^, although the mononuclear species [(Me_4_TACD)CaH(thf)_*x*_]^+^ remains elusive. We show here how the ring‐size of the supporting macrocycle affects the catalytic activity of the catalyst. The fifteen‐membered NNNNN ligand Me_5_PACP (Me_5_PACP=1,4,7,10,13‐pentamethyl‐1,4,7,10,13‐pentaazacyclopentadecane) supports [CaH]^+^ and precludes the coordination of THF at the cationic calcium center. The resulting hydride cation, isolated as a dimer [(Me_5_PACP)_2_Ca_2_(μ‐H)_2_]^2+^, catalyzed the H/D isotopic exchange as well as olefin hydrogenation and hydrosilylation significantly more efficiently than [(Me_4_TACD)_2_Ca_2_(μ‐H)_2_(thf)_*x*_]^2+^ due to easier dissociation into the monomer in solution.

When a solution of [Ca(CH_2_Ph)_2_]^[8]^ in THF was treated with Me_5_PACP, an immediate color change from orange to dark red was observed. NMR spectra of the reaction mixture showed decomposition of the ligand backbone and formation of toluene. Compared with the analogous Me_4_TACD complex,^[7b]^ the putative neutral benzyl complex [(Me_5_PACP)Ca(CH_2_Ph)_2_] appears to be unstable, presumably due to deprotonation of the ligand backbone by the benzyl anion. To circumvent this decomposition pathway, we reacted the conjugated acid of the ligand [(Me_5_PACP)H][BAr_4_] (**1 a**, Ar=C_6_H_3_‐3,5‐Me_2_; **1 b**, Ar=C_6_H_4_‐4‐^n^Bu), conveniently prepared by protonation of Me_5_PACP with [NEt_3_H][BAr_4_], with one equivalent of [Ca(CH_2_Ph)_2_] to give the cationic calcium benzyl complexes [(Me_5_PACP)Ca(CH_2_Ph)][BAr_4_] (**2 a**, Ar=C_6_H_3_‐3,5‐Me_2_; **2 b**, Ar=C_6_H_4_‐4‐^n^Bu) in moderate yields.

Hydrogenolysis of **2 a** and **2 b** with H_2_ (1 bar) gave the hydride complexes [(Me_5_PACP)_2_Ca_2_(μ‐H)_2_][BAr_4_]_2_ (**3 a**, Ar=C_6_H_3_‐3,5‐Me_2_; **3 b**, Ar=C_6_H_4_‐4‐^n^Bu). More conveniently, **3 a** and **3 b** were prepared in a one‐pot synthesis starting from [Ca(CH_2_Ph)_2_] and **1 a**,**b** followed by either hydrogenolysis with H_2_ (1 bar) or by reaction with ^n^octylsilane and isolated in yields of up to 82 %. **3 a** crystallized from THF and remained insoluble in THF as well as aliphatic or aromatic solvents even at elevated temperatures up to 70 °C. Using the anion [B(C_6_H_4_‐4‐^n^Bu)_4_]^−^ increased solubility of the calcium complexes and allowed the one‐pot synthesis to be carried out in benzene, from which THF‐soluble **3 b** precipitated in 80 % yield (Scheme 1).

**Scheme 1:**
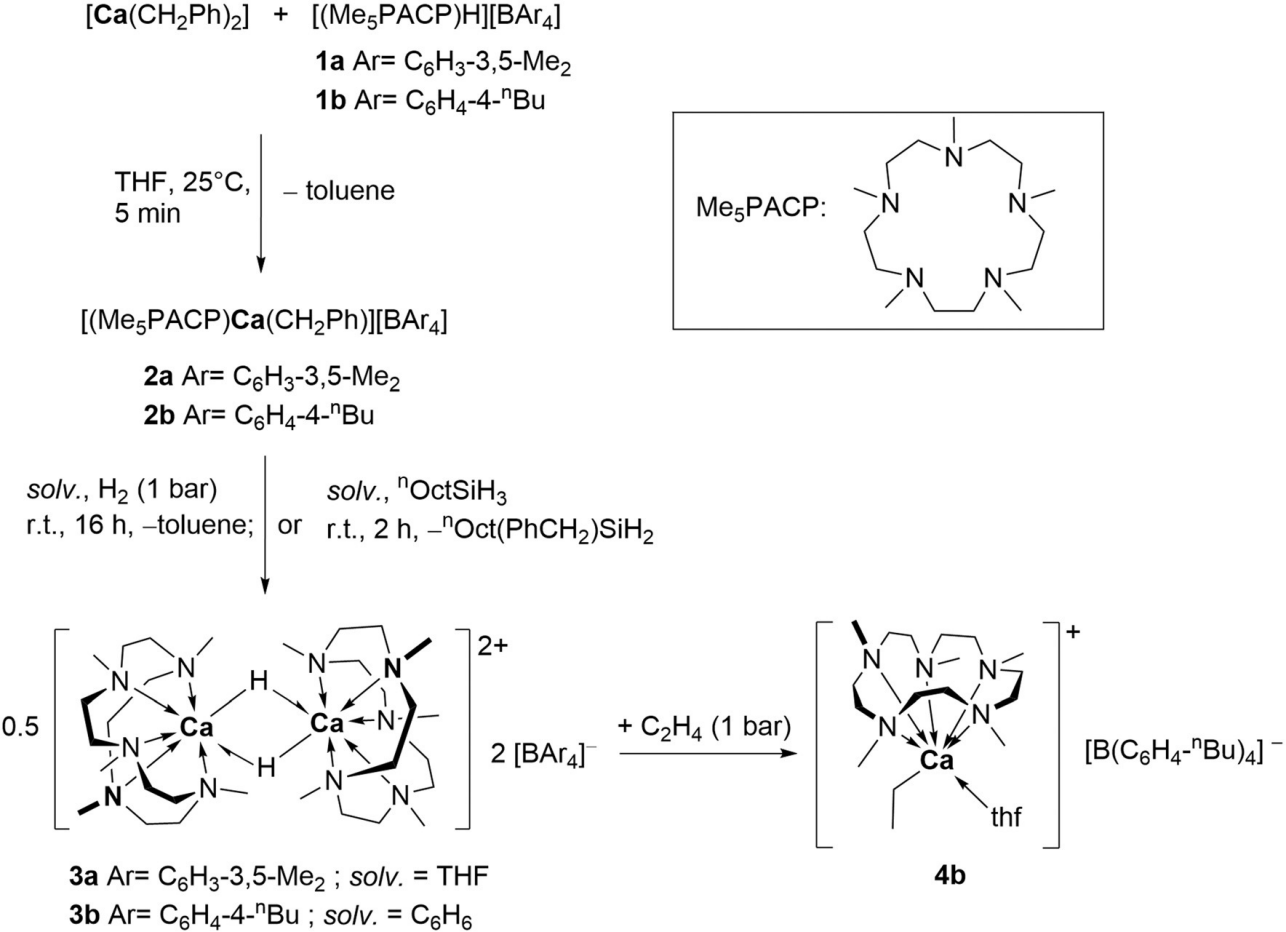
Synthesis of cationic dinuclear Me_5_PACP‐stabilized hydride complexes.

Complex **3 b** crystallizes from THF/n‐pentane as a centrosymmetric dimer with the calcium seven‐coordinate with a distorted pentagonal bipyramidal geometry (Figure 2). The apical positions are occupied by the nitrogen donor N1/N1’ and the hydride ligands. Characteristically the methyl group at the apical position points away from the calcium center as opposed to the methyl groups at the equatorial nitrogen donors N2‐N5. Ca‐N distances range from 2.619(3) to 2.754(2) Å and are slightly longer when compared to those in [(Me_4_TACD)_2_Ca_2_(μ‐H)_2_(thf)]^2+^ (**C**) (2.544(3) to 2.586(3) Å). The Ca⋅⋅⋅Ca distance is 3.7178(10) Å in **3 b** and longer than that in the Me_4_TACD analogue (3.6306(11) Å). The Ca‐H distances of 2.24(2) and 2.27(2) Å in **3 b** are in the same range as for **C** (2.22(3) to 2.34(3) Å).^[7c]^ A folded coordination pattern in Me_4_TACD stabilized complexes has been observed only in the solid state for [(Me_4_TACD)_2_Mg_2_(μ‐O_2_CH)_2_][B(C_6_H_3_‐3,5‐Me_2_)_4_]^[9]^ but is more common for larger macrocyclic ligands.^[10]^ For the complex [(H_5_PACP)CdCl_2_], the coordination of the macrocycle depends on the solvent and both the planar and the folded coordination are maintained in solution.^[11]^ A study regarding the dependency of alkali metal ion size and folding of the macrocycle was carried out for the eighteen‐membered NNNNNN macrocycle Me_6_HACO (Me_6_HACO=1,4,7,10,13,16‐hexamethyl‐1,4,7,10,13,16‐hexaazacyclooctadecane).^[12]^

**Figure 2:**
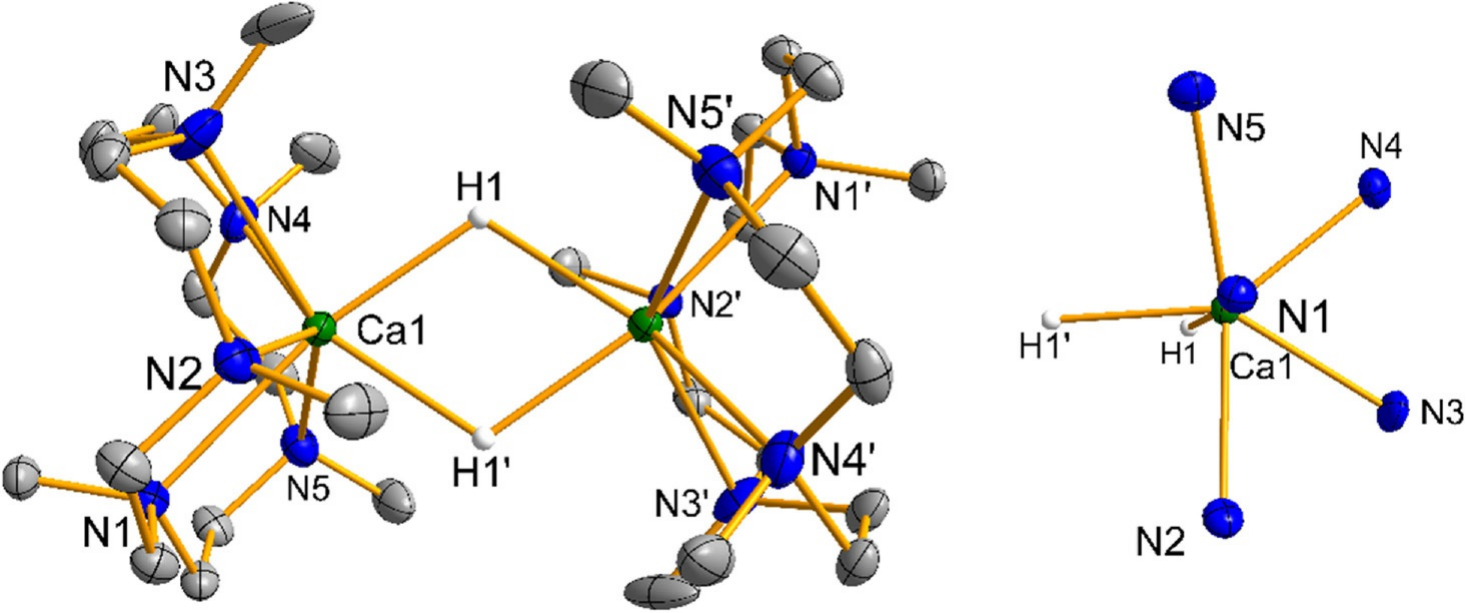
Left: Molecular structure of the dication of **3** **b**. Thermal ellipsoids are set at 30 *%* probability; the anions and the hydrogen atoms except for the hydride ligands are omitted for clarity. Selected interatomic distances [Å]: Ca1⋅⋅⋅Ca1*’* 3.7178(10), Ca1−H1 2.24(2), Ca1−H1*’* 2.27(2), Ca1−N1 2.689(2), Ca1−N2 2.694(2), Ca1−N3 2.633(3), Ca1−N4 2.619(3), Ca1−N5 2.754(2) Å. Right: Top view on the distorted bipyramidal coordination of Ca1.

In line with the molecular structure in the solid state, the NMR spectra of **3 b** in solution show three distinct resonances for the methyl groups in a 2:2:1 ratio. The ^1^H NMR spectrum shows sharp multiplets for the methylene groups at room temperature which indicates a rigid coordination. Above 50 °C, broader signals for the methylene groups indicate conformational fluxionality of the CH_2_CH_2_ linkers. Complex **3 b** decomposes at 50 °C within 6 h. In the ^1^H NMR spectrum of **3 b**, the hydride resonance appears at *δ*=4.63 ppm which is in the expected region for molecular calcium hydrides.[^3a^, ^7b^, ^7c^] The resonance is shifted downfield when compared to the hydride resonance at *δ*=4.46 ppm in **C** and slightly upfield shifted compared to [(Me_4_TACD)_2_Ca_2_(μ‐H)_3_][B(C_6_H_3_‐3,5‐Me_2_)_4_] at *δ*=4.71 ppm.[^7b^, ^7c^] A solution of **3 b** in [D_8_]THF reacted with D_2_ (1 bar) to give **3 b**‐***d***_**2**_ and HD within 3 h. This reaction is faster than that observed for **C** (8 h). In the ^2^H NMR spectrum the deuteride resonance of **3 b**‐***d***_**2**_ appears at *δ*=4.72 ppm. The isotopomer **3 b**–***d***_**1**_ was not observed in this reaction.

When a solution of **3 b** in [D_8_]THF was charged with ethylene (1 bar) at 0 °C, the olefin was smoothly inserted into the Ca−H bond to form an ethyl complex [(Me_5_PACP)Ca(C_2_H_5_)(thf)]^+^ (**4 b**) immediately. Contrary to the slow formation of the Me_4_TACD containing calcium ethyl species,^[7c]^ complete conversion of **3 b** to the cationic ethyl complex was observed, as suggested by the complete disappearance of the hydride resonance of **3 b** in the ^1^H NMR spectrum. The characteristic resonances of the ethyl ligand at *δ*=−0.87 (q) and *δ* 1.29 (t) ppm are shifted when compared to the Me_4_TACD stabilized analogue (*δ*=−1.02 (q), *δ* 1.26 (t) ppm).^[7c]^ The ethyl complex is stable in solution at −20 °C, allowing also ^13^C NMR spectroscopic characterization (*δ*=17.2 and 14.0 ppm). It decomposed at room temperature within 30 min, presumably by deprotonation of the ligand backbone with formation of ethane. Ethylene oligomerization in solution by **4 b** was not observed in contrast to the Me_4_TACD ethyl intermediate [(Me_4_TACD)Ca(C_2_H_5_)(thf)_*x*_]^+^ which quickly oligomerized ethylene to give n‐butyl and n‐hexyl species [(Me_4_TACD)Ca{(C_2_H_4_)_n_H}(thf)_*x*_]^+^ (*n*=2, 3) in [D_8_]THF.^[7c]^ Characterization of **4 b** was mostly limited to in situ NMR spectroscopy due to the low stability of the compound but single crystals of **4 b** grew on a single occasion from a THF/n‐pentane mixture at −40 °C. The crystal structure of **4 b** revealed a mononuclear terminal ethyl complex with one THF molecule coordinated (Scheme 1). Crystallographic details are not discussed due to a possible modulation of the structure (see Supporting Information, Figure S57). When **3 b** was treated with 1‐octene, the formation of an n‐octyl complex was observed by a characteristic triplet at *δ*=−0.76 ppm in the ^1^H NMR spectrum. Such an intermediate was not observed for the reaction with **C**. These findings indicate that the insertion of alkenes is faster and that the n‐alkyl intermediates formed are more stable for [(Me_5_PACP)_2_Ca_2_(μ‐H)_2_]^2+^ when compared to **C**.

Based on the observation that 1‐alkenes readily insert into the Ca−H bond, we assessed the catalytic activity of **3 b** in hydrogenation and hydrosilylation catalysis. In both cases **3 b** showed improved catalytic efficiency compared to **C** under comparable conditions and allowed the catalysis to be carried out even at room temperature (Table 1). 1‐Octene was hydrogenated by 1 bar of H_2_ within 36 h at 25 °C (compared to 24 h at 60 °C).^[7b]^ At elevated temperatures, 1‐octene was hydrogenated within 12 h (40 °C) or 6 h (50 °C). Contrary to **C** and reports on other calcium hydride‐catalyzed hydrogenation, no formation of 2‐octene was observed in this reaction.[^5b^, ^7b^] When 1,4‐hexadiene was used as a substrate, the internal double bond was not hydrogenated as in the case of **C**. Above 50 °C, the catalytic performance of **3 b** dropped, attributable to the relatively low thermal stability of the hydride complex when compared to **C**.

**Table 1:** Hydrogenation and hydrosilylation of unactivated 1‐alkenes catalyzed by complex **3 b**.^[a]^

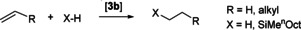						
Entry	Substrate	*T* [°C]	*T* [h]^[b]^	Product	TOF [h^−1^]	TOF [h^−1^] of **C** ^[c]^
1^[d]^	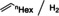	25	36	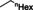	0.5	0.8
2^[d]^	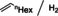	40	12	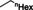	1.6	0.8
3^[d]^	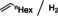	50	6	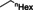	3.3	0.8
4^[d]^	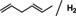	40	12	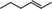	1.6	0.8
5^[e]^	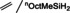	25	0.16	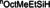	250	160
6^[f]^	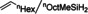	25	36	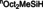	0.5	0.8

[a] 5 mol % of catalyst, 0.1 m of substrate in 0.6 mL of [D_8_]THF, 1,4‐(SiMe_3_)_2_C_6_H_4_ as internal standard. [b] Conversion >95 % as analyzed by ^1^H NMR spectroscopy. [c] TOFs for **C** are given for catalytic runs at 60 °C (hydrogenation) or 70 °C (hydrosilylation).[^7b^, ^7c^] [d] 1 bar of H_2_. [e] 1 bar of ethylene, 2.5 mol % of catalyst [f]<5 % of ^n^OctMe_2_SiH observed as a side product.

Hydrosilylation of ethylene with ^n^OctMeSiH_2_ proceeded rapidly at ambient conditions (25 °C, 1 bar ethylene) within 10 min as compared to 15 min at 70 °C with **C**. Hydrosilylation of 1‐octene with ^n^OctMeSiH_2_ was readily achieved within 36 h at 25 °C. Apart from the main product ^n^Oct_2_MeSiH, traces of ^n^OctMe_2_SiH were detected as a side product. The formation of this hydrosilane can be explained by methyl group transfer. Redistribution of alkyl substituents is not as common as redistribution of aryl substituents in hydrosilanes but has been reported to be catalyzed by transition metal complexes^[13]^ as well as by strong Lewis acids such as silylium ions.^[14]^ In the kinetic experiments using excess hydrosilane (see below), we observed amounts up to 25 % of ^n^OctMe_2_SiH in the product mixture, whereas in the presence of excess 1‐alkene, ^n^Oct_2_MeSiH was formed exclusively.

To assess the origin of the improved catalytic efficiency of [(Me_5_PACP)_2_Ca_2_(μ‐H)_2_]^2+^ compared to [(Me_4_TACD)_2_Ca_2_(μ‐H)_2_(thf)]^2+^ we monitored the hydrosilylation of 1‐octene with ^n^OctMeSiH_2_ at room temperature over the course of 24 h (for [(Me_5_PACP)_2_Ca_2_(μ‐H)_2_]^2+^) or 576 h (for [(Me_4_TACD)_2_Ca_2_(μ‐H)_2_(thf)]^2+^) with varying catalyst and substrate concentrations. As a suitable tool to derive kinetic data from the concentration profiles observed in the NMR spectra we used the Variable Time Normalization Analysis (VTNA) method as described by Burés (see Supporting Information).^[15]^ In order to obtain comparable results for [(Me_5_PACP)_2_Ca_2_(μ‐H)_2_]^2+^ and [(Me_4_TACD)_2_Ca_2_(μ‐H)_2_(thf)]^2+^, we synthesized a Me_4_TACD‐stabilized calcium hydride cation with the more soluble borate anion [B(C_6_H_4_‐4‐^n^Bu)_4_]^−^. [(Me_4_TACD)_2_Ca_2_(μ‐H)_2_(thf)][B(C_6_H_4_‐4‐^n^Bu)_4_]_2_ (**6**) was prepared in analogy to **3 b** in a one‐pot synthesis or starting from the isolated benzyl intermediate [(Me_4_TACD)Ca(CH_2_Ph)][B(C_6_H_4_‐4‐^n^Bu)_4_] (**5**) (see Supporting Information).

The partial reaction orders derived from the VTNA for catalyst, alkene and hydrosilane gave the same values for **3 b** and **6** (Figure 3, see Supporting Information). This indicates that the same reaction mechanism is operative for both cases and rules out the possibility that the higher efficiency of **3 b** is derived from a deviant mechanistic pathway. The partial reaction order in the catalyst is 0.5 which agrees with a monomer‐dimer equilibrium in solution that was previously proposed for the [(Me_4_TACD)_2_Ca_2_(μ‐H)_2_(thf)]^2+^ catalyzed hydrogenation and hydrosilylation.[^7b^, ^7c^, ^16^] The partial reaction order of the alkene is 1 which indicates that the alkene is involved in the rate determining step. This fits the observation that the insertion of alkenes is facilitated for [(Me_5_PACP)_2_Ca_2_(μ‐H)_2_]^2+^ compared to [(Me_4_TACD)_2_Ca_2_(μ‐H)_2_(thf)]^2+^. The observed kinetic isotope effect for the reaction is ≈1 (Supporting Information, Figure S49). This observation shows that while the alkene is involved in the rate determining step, the insertion proceeds equally fast into the Ca‐H and Ca‐D bond. This can be explained if the insertion depends on the concentration of the active catalyst rather than on the nature of the metal‐hydride bond, indicating the importance of the preceding monomer‐dimer equilibrium in the catalytic cycle. In the case of [(Me_5_PACP)_2_Ca_2_(μ‐H)_2_]^2+^ this equilibrium appears to be shifted to the side of the reactive monomer when compared to [(Me_4_TACD)_2_Ca_2_(μ‐H)_2_(thf)]^2+^. This corresponds to a higher concentration of catalytically active species in solution. The partial reaction order for hydrosilane is *pseudo* 0^th^ order, suggesting that the σ‐bond metathesis of the calcium n‐alkyl intermediate with the hydrosilane is faster than the insertion of the alkene. A comparison of the observed reaction rate constants *k*_obs_ derived from the VTNA gave a ratio of *k*_obs_(**3 b**)/*k*_obs_(**6**)≈32.8 (see Supporting Information).

**Figure 3:**
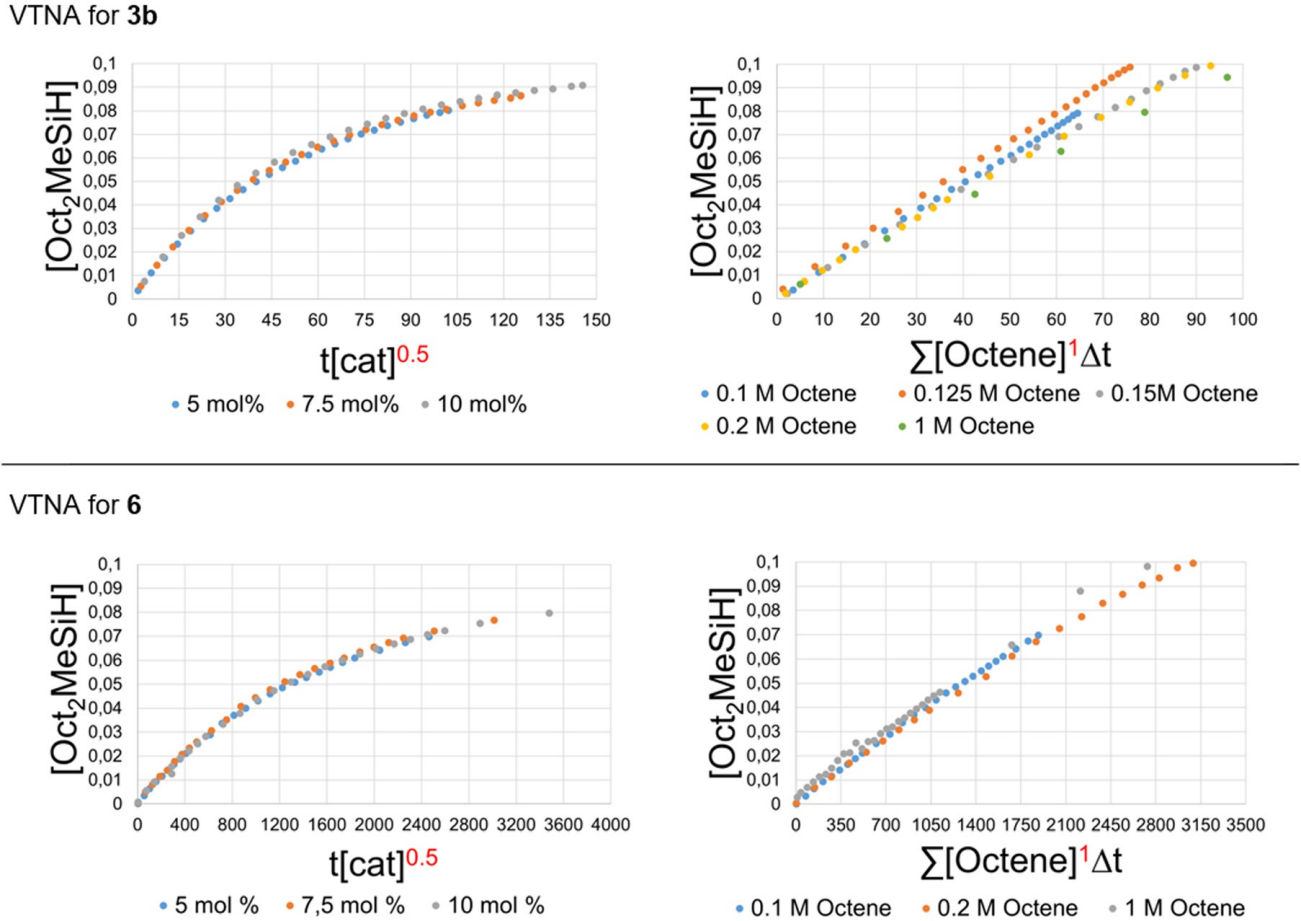
Selected Variable Time Normalization (VTNA) plots demonstrating the partial reaction orders of catalyst and alkenes for the hydrosilylation of 1‐octene mediated by **3** **b** (top) and **6** (bottom). Left: VTNA plot for catalyst, Right: VTNA plot for octene.

DFT calculations (B3PW91) were applied to analyze the bonding in complex **3**. The Ca−H bond distances are fairly well reproduced (2.31 and 2.27 Å vs. 2.27 and 2.24 Å experimentally), indicating the validity of the computational method. Natural Bonding Orbital (NBO) and Molecular Orbitals analysis are consistent with two 3 center‐2 electron Ca‐H‐Ca bonds which are strongly polarized (82 %) toward H (see Supporting Information). Calculations also confirmed the significant exothermicity (−34.1 kcal mol^−1^) of dimer‐monomer dissociation in the presence of THF. This is in stark contrast to the 1‐alkene insertion into the Ca−H bond in [(BDI)Ca(μ‐H]_2_ (**A’**) which forms the n‐alkyl dimer from the hydride dimer without dissociation.[^7a^, ^17^]

In conclusion, the fifteen‐membered NNNNN macrocycle Me_5_PACP stabilizes the reactive fragment [CaH]^+^ as a dinuclear complex with a characteristic pentagonal bipyramidal coordination geometry at the calcium center. This hydride complex shows higher activity in catalytic hydrogenation and hydrosilylation of unactivated alkenes than the previously reported dinuclear hydride complex supported by the twelve‐membered macrocycle Me_4_TACD.[^7b^, ^7c^] Kinetic investigations show that an insertion mechanism is operative in hydrosilylation catalysis. This is further supported by the direct observation of a terminal ethyl complex.[^7a^, ^17b^] The increased catalytic performance may be explained by easier dissociation of the dinuclear hydride into the reactive monomeric calcium hydride cation [(Me_5_PACP)CaH]^+^ in solution when compared to the previously reported cationic calcium hydride complexes.

## Conflict of interest

The authors declare no conflict of interest.

## Supporting information

As a service to our authors and readers, this journal provides supporting information supplied by the authors. Such materials are peer reviewed and may be re‐organized for online delivery, but are not copy‐edited or typeset. Technical support issues arising from supporting information (other than missing files) should be addressed to the authors.

Supplementary
