# Supplementary material for: Calcium Hydride Catalysts for Olefin Hydrofunctionalization: Ring‐Size Effect of Macrocyclic Ligands on Activity
Source: Chemistry. 2021 Jan 18;27(9):3002–7. doi: 10.1002/chem.202004931 (PMC7898310; doi:10.1002/chem.202004931)
Supplement: Supplementary file 1 — Supplementary [file CHEM-27-3002-s001.pdf]

# Chemistry–A European Journal

Supporting Information


## Calcium Hydride Catalysts for Olefin Hydrofunctionalization: Ring-Size Effect of Macrocyclic Ligands on Activity


Thomas Höllerhage,[a] Danny Schuhknecht,[a] Alisha Mistry,[a] Thomas P. Spaniol,[a] Yan Yang,[b] Laurent Maron,*[b] and Jun Okuda*[a]

## Table of Contents


1 General Remarks ........ 3

2 Synthetic Procedures and Characterizations ........ 4

2.1 Synthesis of $[NEt_3H][B(C_6H_4\text{-}4\text{-}^nBu)_4]$. ........ 4

2.2 NMR and IR Spectra of $[NEt_3H][B(C_6H_4\text{-}4\text{-}^nBu)_4]$. ........ 5

2.3 Synthesis of $[(Me_4TACD)H][B(C_6H_4\text{-}4\text{-}^nBu)_4]$. ........ 7

2.4 NMR Spectra of $[(Me_4TACD)H][B(C_6H_4\text{-}4\text{-}^nBu)_4]$ ........ 8

2.5 Synthesis of $[(Me_5PACP)H][B(C_6H_3\text{-}3,5\text{-}Me_2)_4]$ (**1a**) ........ 10

2.6 NMR Spectra of $[(Me_5PACP)H][B(C_6H_3\text{-}3,5\text{-}Me_2)_4]$ (**1a**) ........ 11

2.7 Synthesis of $[(Me_5PACP)H][B(C_6H_4\text{-}4\text{-}^nBu)_4]$ (**1b**). ........ 13

2.8 NMR and IR Spectra of $[(Me_5PACP)H][B(C_6H_4\text{-}4\text{-}^nBu)_4]$ (**1b**). ........ 14

2.9 Molecular Structure of $[(Me_5PACP)H][B(C_6H_4\text{-}4\text{-}^nBu)_4]$ (**1b**). ........ 16

2.10 Synthesis of $[(Me_5PACP)Ca(CH_2Ph)][B(C_6H_3\text{-}3,5\text{-}Me_2)_4]$ (**2a**). ........ 17

2.11 NMR Spectra of $[(Me_5PACP)Ca(CH_2Ph)][B(C_6H_3\text{-}3,5\text{-}Me_2)_4]$ (**2a**). ........ 18

2.12 Synthesis of $[(Me_5PACP)Ca(CH_2Ph)][B(C_6H_4\text{-}4\text{-}^nBu)_4]$ (**2b**) ........ 20

2.13 NMR Spectra of $[(Me_5PACP)Ca(CH_2Ph)][B(C_6H_4\text{-}4\text{-}^nBu)_4]$ (**2b**) ........ 21

2.14 Synthesis of $[(Me_5PACP)Ca(\mu\text{-}H)]_2[B(C_6H_3\text{-}3,5\text{-}Me_2)_4]_2$ (**3a**). ........ 23

2.15 Synthesis of $[(Me_5PACP)_2Ca_2(\mu\text{-}H)_2][B(C_6H_4\text{-}4\text{-}^nBu)_4]_2$ and $[(Me_5PACP)_2Ca_2(\mu\text{-}D)_2]$-$[B(C_6H_4\text{-}4\text{-}^nBu)_4]_2$. (**3b/ 3b-*d*$_2$**). ........ 24

2.16 NMR Spectra of $[(Me_5PACP)_2Ca_2(\mu\text{-}H)_2][B(C_6H_4\text{-}4\text{-}^nBu)_4]_2$ and $[(Me_5PACP)_2Ca_2(\mu\text{-}D)_2][B(C_6H_4\text{-}4\text{-}^nBu)_4]_2$ (**3b/ 3b-*d*$_2$**). ........ 25

2.17 Molecular Structure of $[(Me_5PACP)_2Ca_2(\mu\text{-}H)_2][B(C_6H_4\text{-}4\text{-}^nBu)_4]_2$ (**3b**). ........ 29

2.18 Synthesis of $[(Me_4TACD)Ca(CH_2Ph)][B(C_6H_4\text{-}4\text{-}^nBu)_4]$ (**5**). ........ 30

2.19 NMR spectra of $[(Me_4TACD)Ca(CH_2Ph)][B(C_6H_4\text{-}4\text{-}^nBu)_4]$ (**5**). ........ 31

2.20 Synthesis of [(Me$_4$TACD)$_2$Ca$_2$(μ-H)$_2$(thf)][B(C$_6$H$_4$-4-$^n$Bu)$_4$]$_2$ (**6**). ....33
2.21 NMR Spectra of [(Me$_4$TACD)$_2$Ca$_2$(μ-H)$_2$(thf)][B(C$_6$H$_4$-4-$^n$Bu)$_4$]$_2$ (**6**). ....34
2.22 Molecular Structure of [(Me$_4$TACD)$_2$Ca$_2$(μ-H)$_2$(thf)][B(C$_6$H$_4$-4-$^n$Bu)$_4$]$_2$ (**6**). ....35
3 Reactivity Studies and Kinetic Investigations ....36
3.1 Stoichiometric Reactions ....36
3.2 Hydrogenation and Hydrosilylation Catalysis ....40
3.3 Kinetic Investigations ....41
4 Crystal Structure Determinations ....51
5 Computational Details ....54
6 References ....64

# 1 General Remarks

All operations were performed under an inert atmosphere of dry argon using standard Schlenk line or glovebox techniques. THF-$d_8$, THF, benzene and n-pentane were distilled under argon from sodium/benzophenone ketyl prior to use. Hydrogen (99.999%) and deuterium (99.8%) were purchased from Praxair-Westfalen AG. Commercially available reagents were dried over $CaH_2$ and distilled, or in case of solids, sublimed before use. $^{1}H$, $^{2}H$, $^{11}B\{^{1}H\}$, $^{13}C\{^{1}H\}$, and $^{29}Si\{^{1}H\}$ spectra were recorded on a Bruker Avance II 400 or a Bruker Avance III HD 400 spectrometer at 25 °C in J. Young-type NMR tubes. Chemical shifts for $^{1}H$, $^{2}H$ and $^{13}C\{^{1}H\}$ spectra were referenced internally using the residual solvent resonance and are reported relative to tetramethylsilane. $^{11}B\{^{1}H\}$ and $^{29}Si\{^{1}H\}$ were referenced externally to $BF_3(OEt_2)$ and $SiMe_4$. The resonances in the $^{1}H$ and $^{13}C\{^{1}H\}$ NMR spectra were assigned on the basis of two-dimensional NMR experiments (COSY, HSQC, HMBC). IR spectra were measured as KBr pellets using a Nicolet AVATAR 360 FT-IR spectrometer. Elemental analyses were performed on an Elementar Vario EL instrument. In several instances, the carbon values were not satisfactory, possibly due to incomplete combustion as the result of carbonate or carbide formation as well as high air- and moisture sensitivity.[S1] $[Ca(CH_2Ph)_2]$[S2], 1,4,7,10-tetramethyl-1,4,7,10-tetraazacyclododecane ($Me_4TACD$)[S3], 1,4,7,10,13-pentamethyl-1,4,7,10,13-penta-azacyclopentadecane ($Me_5PACP$)[S4] and $[NEt_3H][B(C_6H_3\text{-}3,5\text{-}Me_2)_4]$[S5] were synthesized according to the literature.

# 2 Synthetic Procedures and Characterizations

## 2.1 Synthesis of $[NEt_3H][B(C_6H_4\text{-}4\text{-}^nBu)_4]$.

In a Schlenk-flask, magnesium turnings (4.50 g, 0.19 mol) were suspended in THF (50 mL). 1,2-Dibromoethane (0.1 mL, 1.16 mmol) in THF (20 mL) was added and the mixture stirred for 5 min. 1-Bromo-4-butylbenzene (25.0 g, 0.12 mol) in THF (150 mL) was then added dropwise over the course of 30 min (exothermic formation of the Grignard-species started immediately after addition of 1-bromo-4-butylbenzene). The reaction mixture was than stirred for 16 h after which $Na[BF_4]$ (2.80 g, 25.5 mmol) was added and the mixture stirred for further 24 h. Aqueous solution of $Na_2CO_3$ was added and the mixture stirred for 30 min, extracted with a mixture of acetonitrile and diethyl ether (3 x 100mL/50 mL) and the combined organic layers dried over $MgSO_4$. After filtration, the solvents were removed under reduced pressure and the obtained off-white powder was dissolved in a mixture of methanol and water (100 mL/50 mL). To this mixture, a solution of $[NEt_3H]Cl$ (5.00 g, 36.3 mmol) in water (100 mL) was added dropwise and the resulting suspension stirred for 10 min. The suspension was filtered, the solid washed with methanol (50 mL) and water (3 x 100 mL) and dried under reduced pressure to give $[NEt_3H][B(C_6H_4\text{-}4\text{-}^nBu)_4]$ as a colorless powder (11.1 g, 17.2 mmol); yield: 67%.

$^1H$ NMR (400 MHz, $CD_3CN$, 25 °C): δ = 0.92 (t, $^3J_{HH}$ = 7.3 Hz, 12H, Ar-$CH_2CH_2CH_2C\mathit{H}_3$), 1.22 (t, $^3J_{HH}$ = 7.3 Hz, 9H, $NCH_2C\mathit{H}_3$), 1.29 − 1.39 (m, 8H, Ar-$CH_2CH_2C\mathit{H}_2CH_3$), 1.49 − 1.59 (m, 8H, Ar-$CH_2C\mathit{H}_2CH_2CH_3$), 2.47 (t, $^3J_{HH}$ = 7.8 Hz, 8H, Ar-$C\mathit{H}_2CH_2CH_2CH_3$), 3.11 (q, $^3J_{HH}$ = 7.3 Hz, 6H, $NC\mathit{H}_2CH_3$), 6.82 (d, $^3J_{HH}$ = 7.7 Hz, 8H, *meta*-$C_6H_4$), 7.17 (dq, $^3J_{HH}$ = 7.7 Hz, $^3J_{BH}$ = 2.7 Hz, 8H, *ortho*-$C_6H_4$) ppm.

$^{13}C\{^1H\}$ NMR (101 MHz, $CD_3CN$, 25 °C): δ = 9.2 (s, $NCH_2\mathit{C}H_3$), 14.3 (s, Ar-$CH_2CH_2CH_2\mathit{C}H_3$), 23.4 (s, Ar-$CH_2CH_2\mathit{C}H_2CH_3$), 35.1 (s, Ar-$CH_2\mathit{C}H_2CH_2CH_3$), 36.1 (s, Ar-$\mathit{C}H_2CH_2CH_2CH_3$), 48.1 (s, $N\mathit{C}H_2CH_3$), 126.7 (q, $^3J_{BC}$ = 2.8 Hz, *meta*-$C_6H_4$), 136.4 (s, *para*-$C_6H_4$), 136.6 (q, $^2J_{BC}$ = 1.4 Hz, *ortho*-$C_6H_4$), 162.0 (q, $^1J_{BC}$ = 49.6 Hz, *ipso*-$C_6H_4$) ppm.

$^{11}B\{^1H\}$ NMR (128 MHz, $CD_3CN$, 25°C): δ = −7.14 ppm.

Anal. Calc. for $C_{46}H_{68}BN$ (645.87 g $mol^{-1}$): C, 85.54; H, 10.61; N, 2.17. Found: C, 85.18; H, 10.50; N, 2.53%.

## 2.2 NMR and IR Spectra of $[NEt_3H][B(C_6H_4\text{-}4\text{-}^nBu)_4]$.

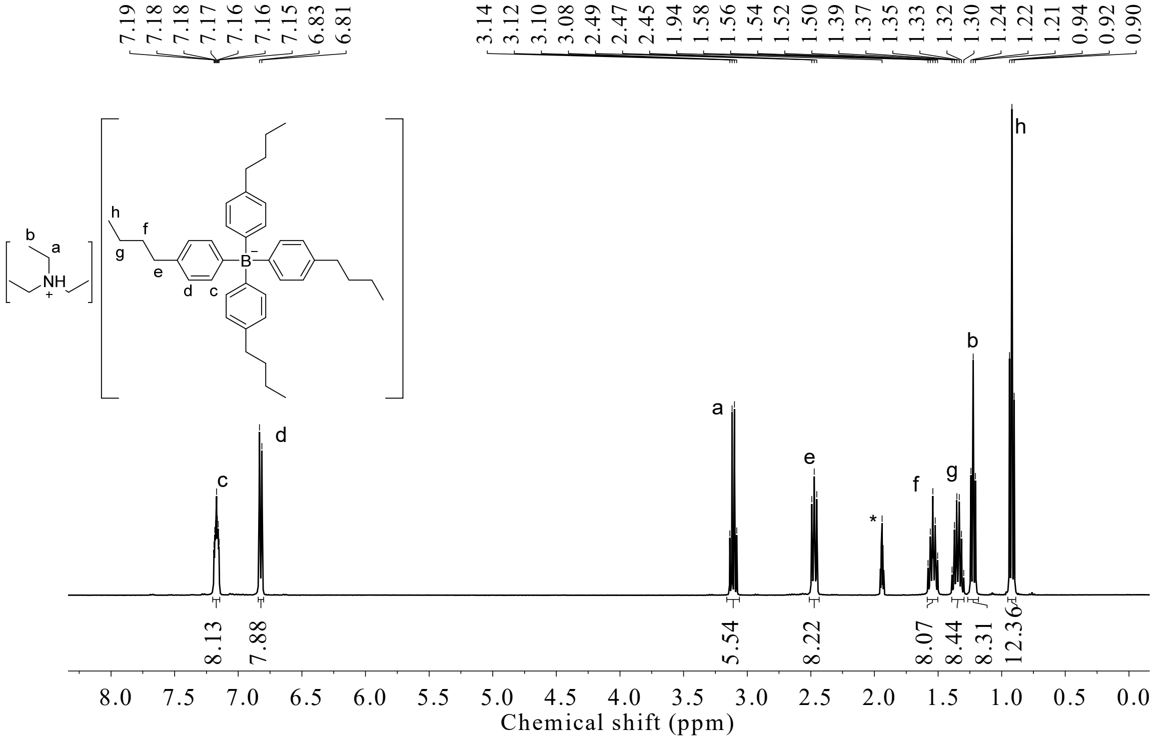


Figure S1: $^1H$ NMR spectrum (400 MHz, $CD_3CN$ (*), 25 °C) of $[NEt_3H][B(C_6H_4\text{-}4\text{-}^nBu)_4]$.

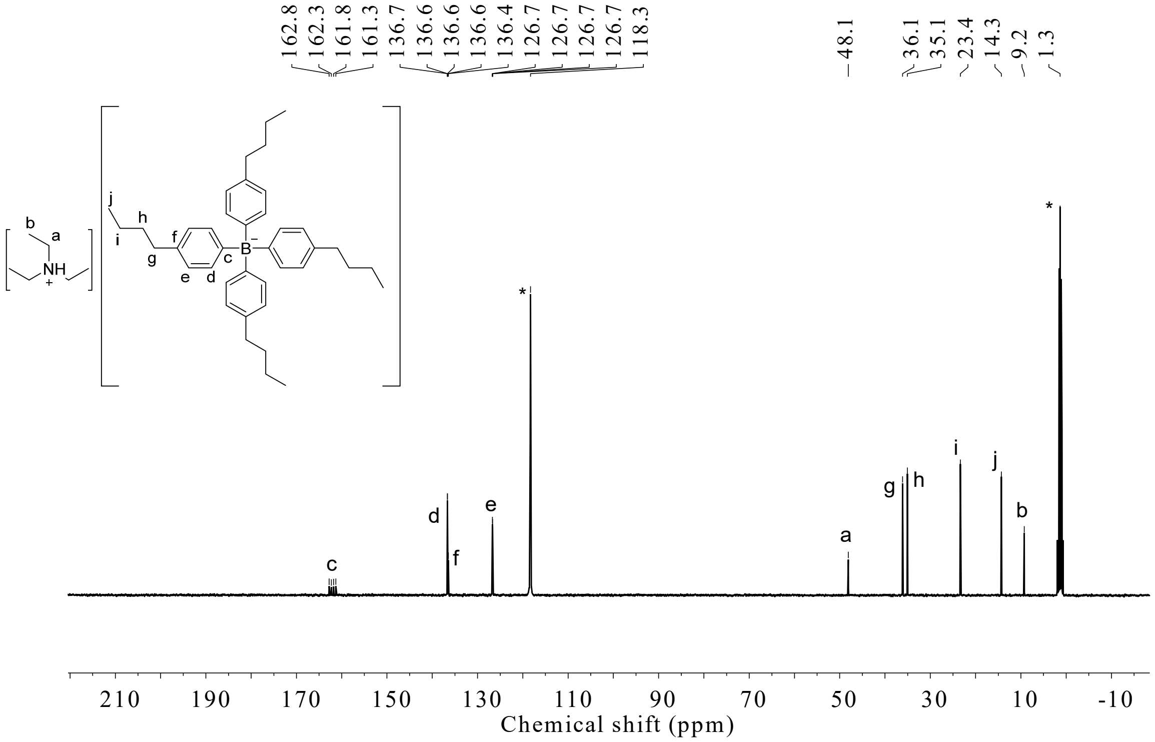


Figure S2: $^{13}C\{^1H\}$ NMR spectrum (101 MHz, $CD_3CN$ (*), 25 °C) of $[NEt_3H][B(C_6H_4\text{-}4\text{-}^nBu)_4]$.

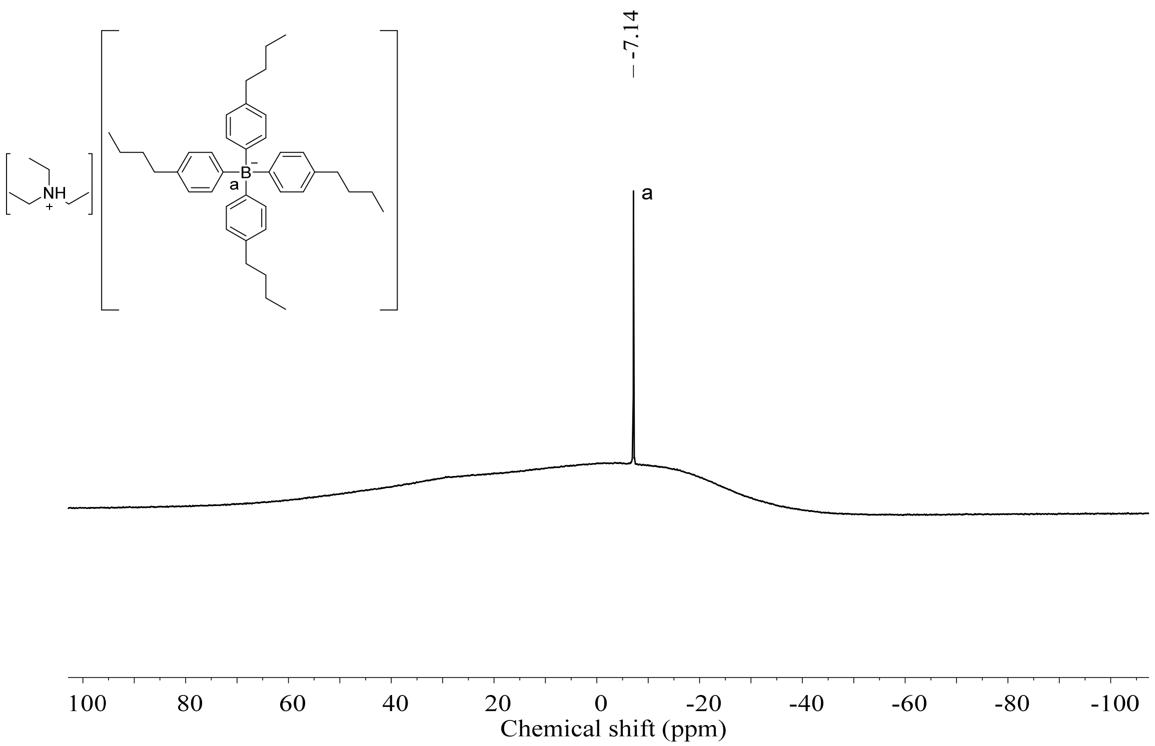


Figure S3: $^{11}B\{^{1}H\}$ NMR spectrum (128 MHz, $CD_3CN$, 25 °C) of $[NEt_3H][B(C_6H_4\text{-}4\text{-}^{n}Bu)_4]$.

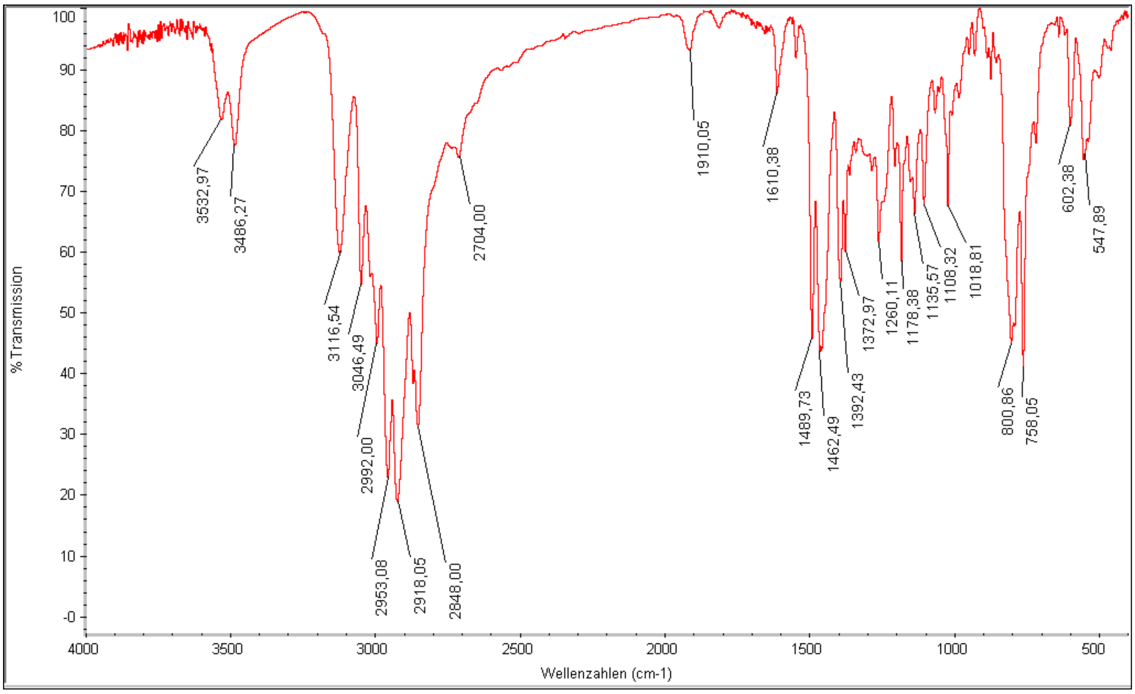


Figure S4: IR spectrum (KBr) of $[NEt_3H][B(C_6H_4\text{-}4\text{-}^{n}Bu)_4]$.

## 2.3 Synthesis of $[(Me_4TACD)H][B(C_6H_4\text{-}4\text{-}^nBu)_4]$.

To a solution of $Me_4TACD$ (251 mg, 1.10 mmol) in benzene (5 mL), was added in portion $[NEt_3H][B(C_6H_4\text{-}4\text{-}^nBu)_4]$ (646 mg, 1.00 mmol) and the resulting reaction mixture stirred for 5 min, filtered and layered with n-pentane (10 mL). After standing for 16 h at room temperature, colorless crystals formed which were isolated, washed with n-pentane (3 x 10 mL) and dried under reduced pressure to give $[(Me_4TACD)H][B(C_6H_4\text{-}4\text{-}^nBu)_4]$ as colorless crystals (640 mg, 0.83 mmol); yield: 83%.

$^1$H NMR (400 MHz, THF-$d_8$, 25 °C): δ = 0.92 (t, $^3J_{HH}$ = 7.3 Hz, 12H, Ar-$CH_2CH_2CH_2C\mathit{H}_3$), 1.31 − 1.41 (m, 8H, Ar-$CH_2CH_2C\mathit{H}_2CH_3$), 1.53 − 1.61 (m, 8H, Ar-$CH_2C\mathit{H}_2CH_2CH_3$), 2.25 (s, 12H, $NC\mathit{H}_3$), 2.41 (s, 16H, $NC\mathit{H}_2$), 2.46 (t, $^3J_{HH}$ = 7.8 Hz, 8H, Ar-$C\mathit{H}_2CH_2CH_2CH_3$), 6.73 (d, $^3J_{HH}$ = 7.7 Hz, 8H, *meta*-$C_6H_4$), 7.20 (dq, $^3J_{HH}$ = 7.7 Hz, $^3J_{BH}$ = 2.7 Hz, 8H, *ortho*-$C_6H_4$), 9.95 (br, 1H, NH) ppm.

$^{13}$C{$^1$H} NMR (101 MHz, THF-$d_8$, 25 °C): δ = 14.7 (s, Ar-$CH_2CH_2CH_2\mathit{C}H_3$), 23.7 (s, Ar-$CH_2CH_2\mathit{C}H_2CH_3$), 35.6 (s, Ar-$CH_2\mathit{C}H_2CH_2CH_3$), 36.9 (s, Ar-$\mathit{C}H_2CH_2CH_2CH_3$), 43.0 (s, $N\mathit{C}H_3$), 54.4 (s, $N\mathit{C}H_2$) 126.2 (q, $^3J_{BC}$ = 2.8 Hz, *meta*-$C_6H_4$), 135.3 (s, *para*-$C_6H_4$), 137.3 (q, $^2J_{BC}$ = 1.4 Hz, *ortho*-$C_6H_4$), 162.5 (q, $^1J_{BC}$ = 49.6 Hz, *ipso*-$C_6H_4$) ppm.

$^{11}$B{$^1$H} NMR (128 MHz, THF-$d_8$, 25°C): δ = −7.14 ppm.

Anal. Calc. for $C_{52}H_{81}BN_4$ (773.06 g mol$^{-1}$): C, 80.79; H, 10.56; N, 7.25. Found: C, 80.54; H, 10.66; N, 7.48%.

## 2.4 NMR Spectra of $[(Me_4TACD)H][B(C_6H_4\text{-}4\text{-}^nBu)_4]$.

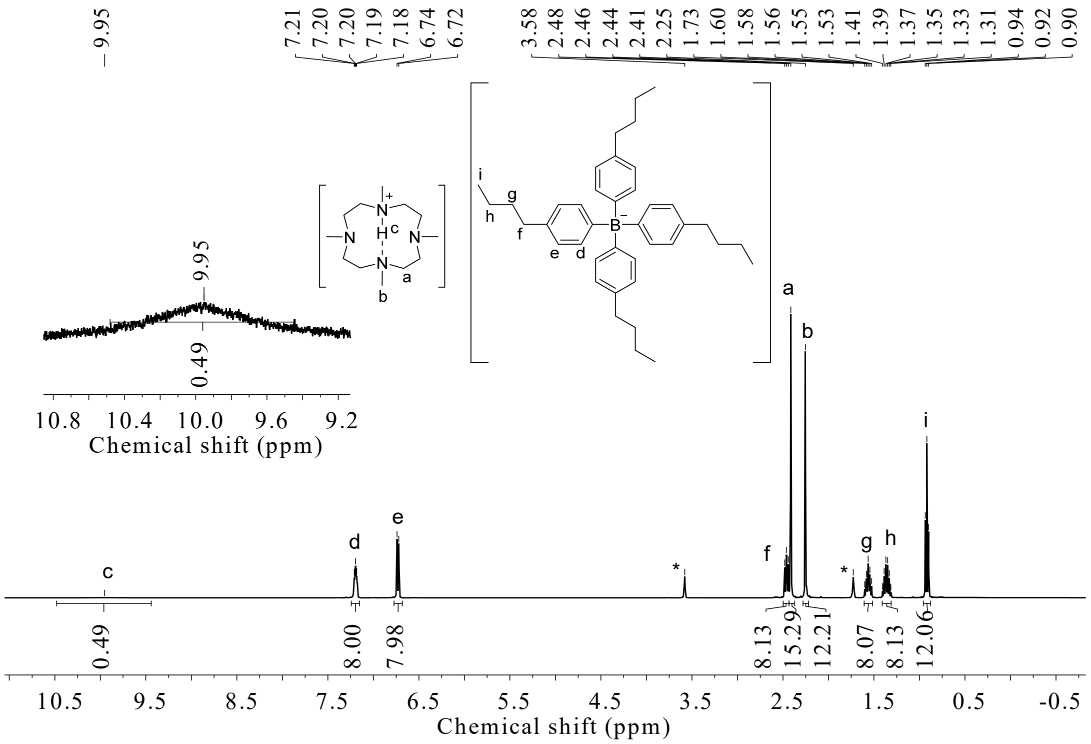


Figure S5: $^{1}$H NMR spectrum (400 MHz, THF-$d_8$ (*), 25 °C) of $[(Me_4TACD)H][B(C_6H_4\text{-}4\text{-}^nBu)_4]$.

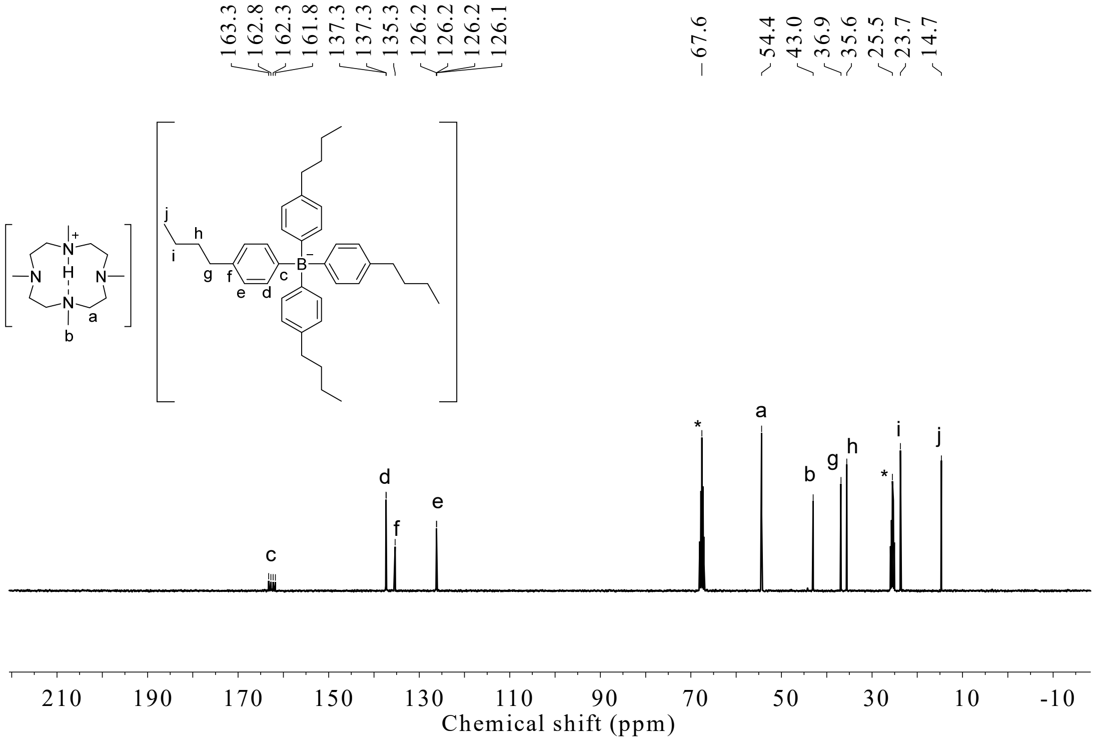


Figure S6: $^{13}C\{^1H\}$ NMR spectrum (101 MHz, THF-$d_8$ (*), 25 °C) of $[(Me_4TACD)H][B(C_6H_4\text{-}4\text{-}^nBu)_4]$.

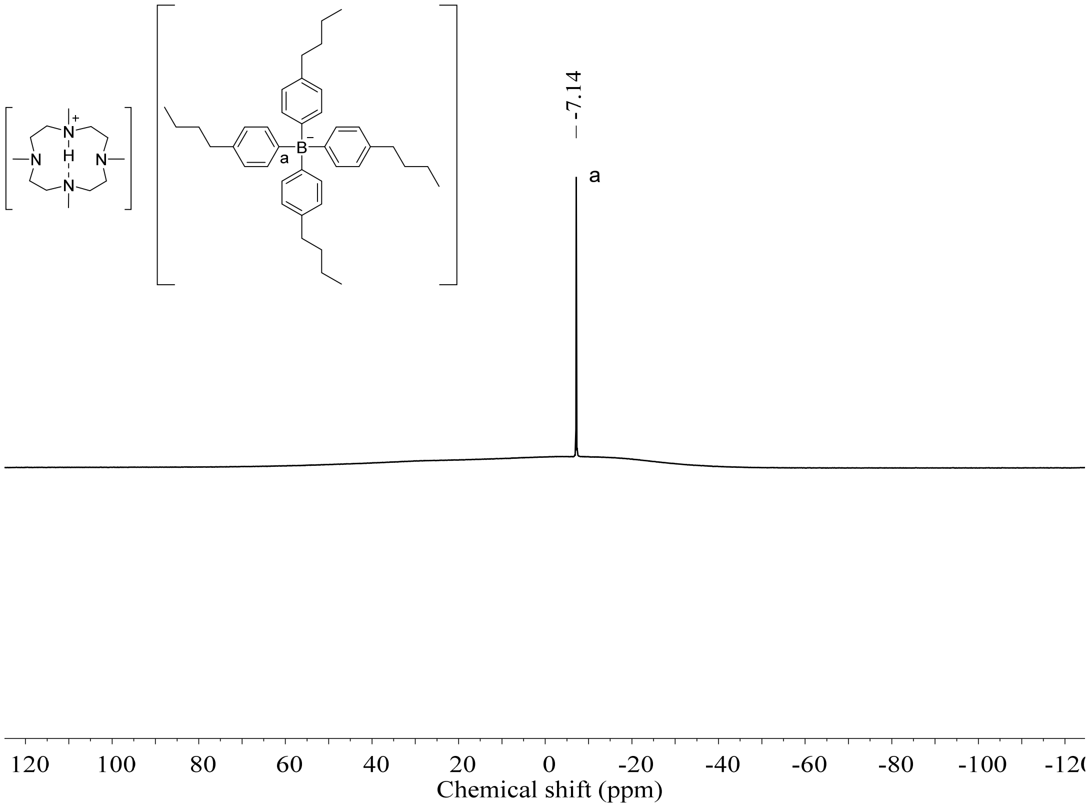


Figure S7: $^{11}B\{^{1}H\}$ NMR spectrum (128 MHz, THF-$d_8$, 25 °C) of $[(Me_4TACD)H][B(C_6H_4\text{-}4\text{-}^nBu)_4]$.

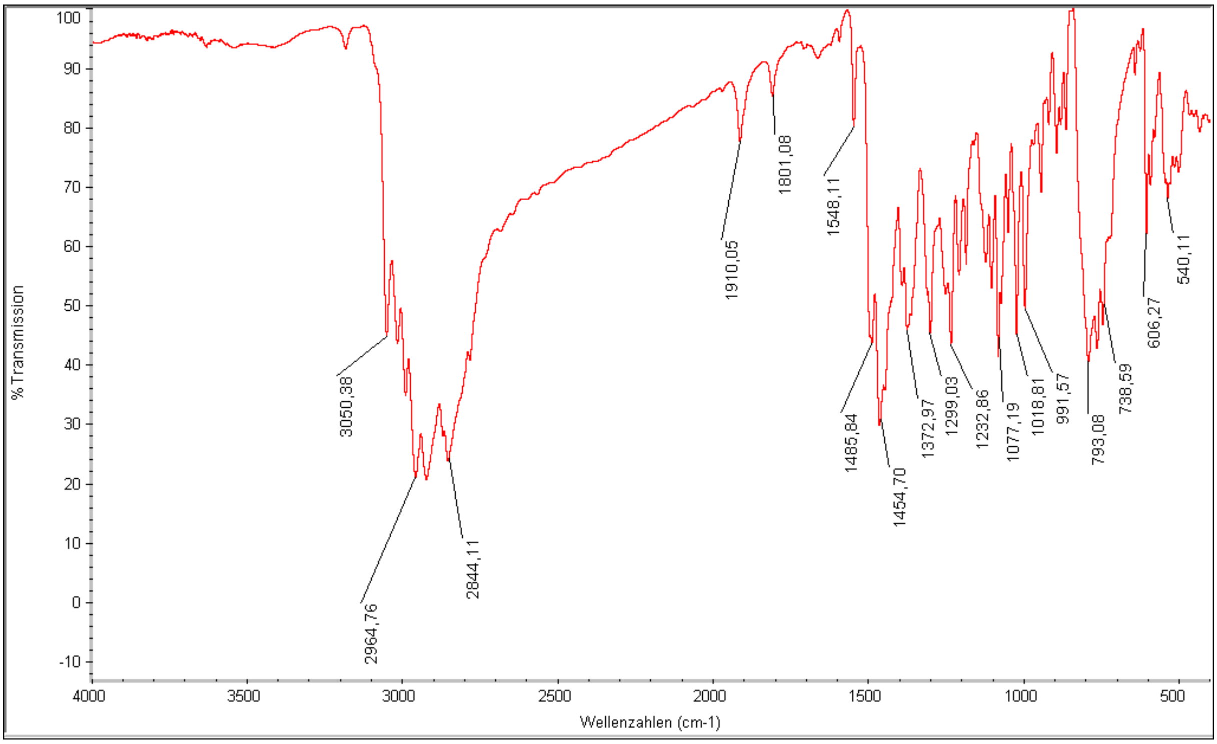


Figure S8: IR spectrum (KBr) of $[NEt_3H][B(C_6H_4\text{-}4\text{-}^nBu)_4]$.

## 2.5 Synthesis of [(Me$_5$PACP)H][B(C$_6$H$_3$-3,5-Me$_2$)$_4$] (**1a**).

To a stirred suspension of [NEt$_3$H][B(C$_6$H$_3$-3,5-Me$_2$)$_4$] (534 mg, 1.00 mmol) in Et$_2$O (10 mL), was added dropwise Me$_5$PACP (300 mg, 1.05 mmol) at room temperature. After stirring for 1 h, a colorless precipitate formed. The solid was isolated by centrifugation, washed with n-pentane (3 x 2 mL) and dried under reduced pressure to give [(Me$_5$PACP)H][B(C$_6$H$_3$-3,5-Me$_2$)$_4$] as a colorless solid (682 mg, 0.95 mmol); yield : 95%.

$^1$H NMR (400 MHz, THF-*d*$_8$ , 25 °C): δ = 2.11 (s, 24 H, Ar-C*H*$_3$), 2.17 (s, 15 H, NC*H*$_3$), 2.27 (s, 20 H, NC*H*$_2$), 6.37 − 6.42 (m, 4 H, *para*-C$_6$H$_3$), 7.01 − 7.06 (m, 8 H, *ortho*-C$_6$H$_3$), 8.34 (br, 1 H, N*H*) ppm.

$^{13}$C{$^1$H} NMR (101 MHz, THF-*d*$_8$, 25 °C): δ = 22.4 (s, Ar-*C*H$_3$), 43.4 (s, N*C*H$_3$), 54.5 (s, N*C*H$_2$), 123.9 (*para*-C$_6$H$_3$), 133.4 (q, $^3J_{BC}$ = 2.7 Hz, *meta*-C$_6$H$_3$), 135.6 (*ortho*-C$_6$H$_3$), 165.9 (q, $^1J_{BC}$ = 49.1 Hz, *ipso*- C$_6$H$_3$) ppm.

$^{11}$B{$^1$H} NMR (128 MHz, THF-*d*$_8$, 25°C): δ = −6.96 ppm.

Anal. calc. for C$_{47}$H$_{72}$BN$_5$ (717.94 g mol$^{-1}$): C, 78.63; H, 10.11; N, 9.76. Found: C, 78.51; H, 9.91; N, 9.99%.

## 2.6 NMR Spectra of [(Me$_5$PACP)H][B(C$_6$H$_3$-3,5-Me$_2$)$_4$] (**1a**).

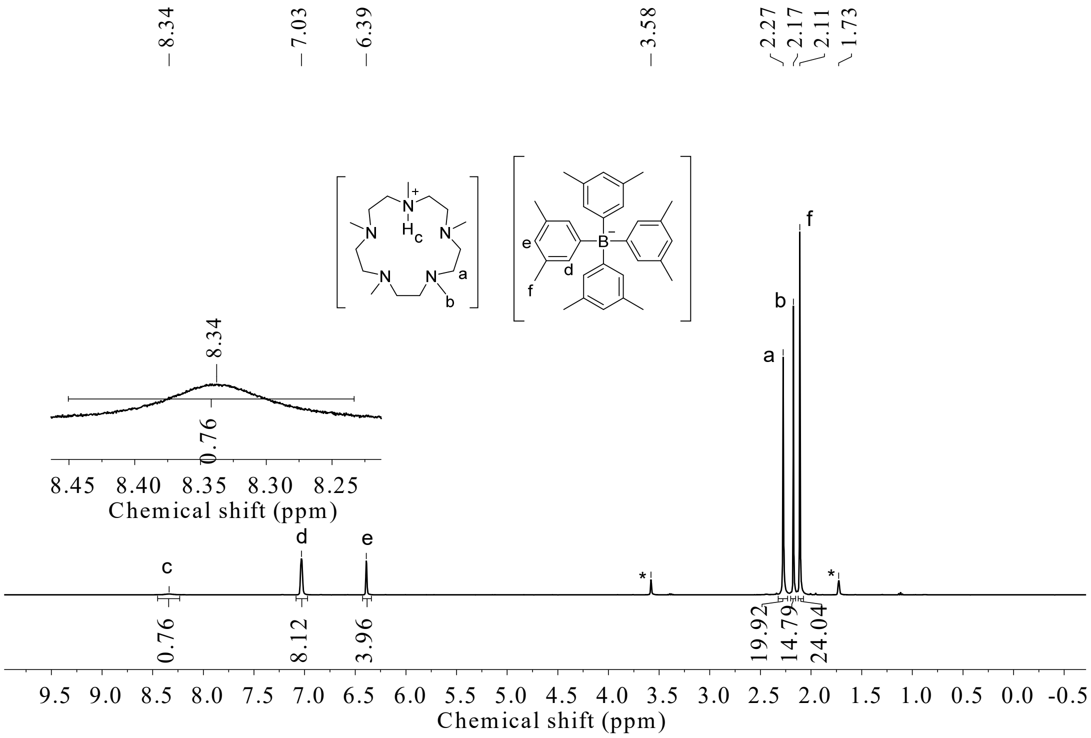


Figure S9: $^1$H NMR spectrum (400 MHz, THF-$d_8$ (*), 25 °C) of [(Me$_5$PACP)H][B(C$_6$H$_3$-3,5-Me$_2$)$_4$].

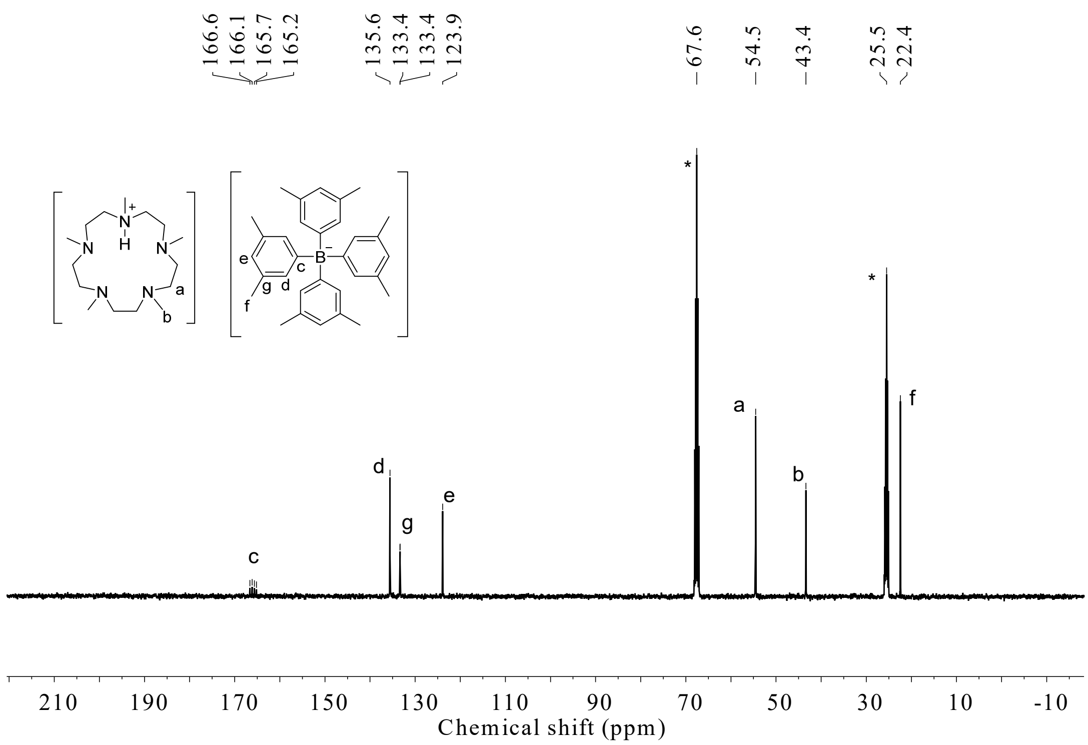


Figure S10: $^{13}$C{$^1$H} NMR spectrum (101 MHz, THF-$d_8$ (*), 25 °C) of [(Me$_5$PACP)H][B(C$_6$H$_3$-3,5-Me$_2$)$_4$].

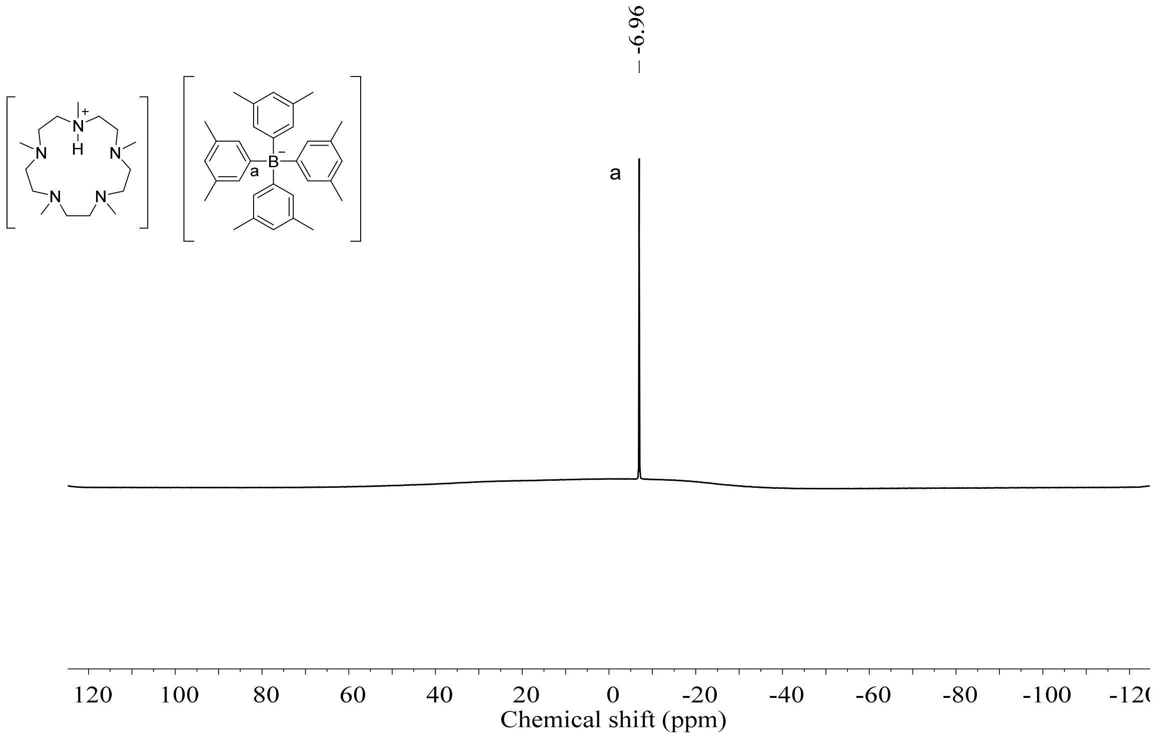


Figure S11: $^{11}B\{^{1}H\}$ NMR spectrum (128 MHz, THF-$d_8$, 25 °C) of [(Me$_5$PACP)H][B(C$_6$H$_3$-3,5-Me$_2$)$_4$].

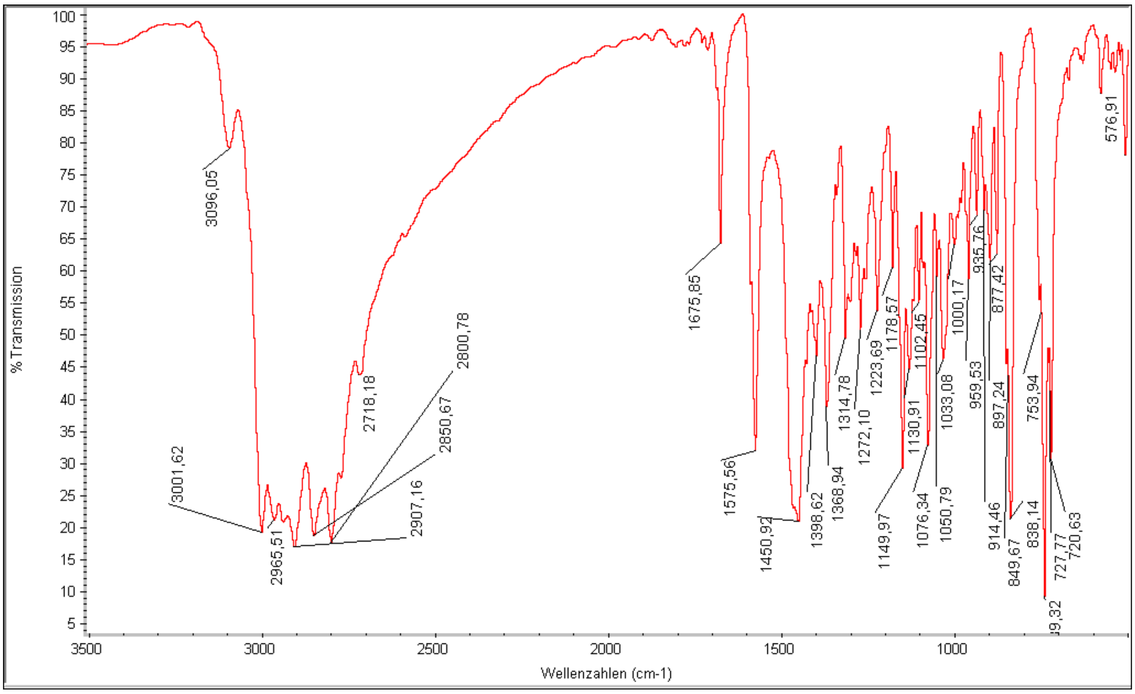


Figure S12: IR spectrum (KBr) of [(Me$_5$PACP)H][B(C$_6$H$_3$-3,5-Me$_2$)$_4$].

## 2.7 Synthesis of [($Me_5$PACP)H][B($C_6H_4$-4-$^{n}$Bu)$_4$] (**1b**).

To a solution of $Me_5$PACP (343 mg, 1.20 mmol) in benzene (5 mL), was added in portions [$NEt_3$H][B($C_6H_4$-4-$^{n}$Bu)$_4$] (646 mg, 1.00 mmol) and the resulting reaction mixture stirred for 5 min, filtered and layered with n-pentane (10 mL). After standing for 16 h at room temperature, colorless crystals formed which were isolated, washed with n-pentane (3 x 10 mL) and dried under reduced pressure to give [($Me_5$PACP)H][B($C_6H_4$-4-$^{n}$Bu)$_4$] as colorless crystals (751 mg, 0.90 mmol); yield: 90%.

$^{1}$H NMR (400 MHz, THF-$d_8$, 25 °C): δ = 0.92 (t, $^{3}J_{HH}$ = 7.3 Hz, 12H, Ar-$CH_2CH_2CH_2C\mathit{H}_3$), 1.31 − 1.41 (m, 8H, Ar-$CH_2CH_2C\mathit{H}_2CH_3$), 1.53 − 1.60 (m, 8H, Ar-$CH_2C\mathit{H}_2CH_2CH_3$), 2.23 (s, 15H, N$C\mathit{H}_3$), 2.39 (s, 20H, N$C\mathit{H}_2$), 2.46 (t, $^{3}J_{HH}$ = 7.8 Hz, 8H, Ar-$C\mathit{H}_2CH_2CH_2CH_3$), 6.73 (d, $^{3}J_{HH}$ = 7.7 Hz, 8H, *meta*-$C_6H_4$), 7.19 (dq, $^{3}J_{HH}$ = 7.7 Hz, $^{3}J_{BH}$ = 2.7 Hz, 8H, *ortho*-$C_6H_4$), 8.34 (br, 1H, NH) ppm.

$^{13}$C{$^{1}$H} NMR (101 MHz, THF-$d_8$, 25 °C): δ = 14.7 (s, Ar-$CH_2CH_2CH_2\mathit{C}H_3$), 23.7 (s, Ar-$CH_2CH_2\mathit{C}H_2CH_3$), 35.5 (s, Ar-$CH_2\mathit{C}H_2CH_2CH_3$), 36.9 (s, Ar-$\mathit{C}H_2CH_2CH_2CH_3$), 43.5 (s, N$\mathit{C}H_3$), 54.7 (s, N$\mathit{C}H_2$) 126.2 (q, $^{3}J_{BC}$ = 2.8 Hz, *meta*-$C_6H_4$), 135.4 (s, *para*-$C_6H_4$), 137.3 (q, $^{2}J_{BC}$ = 1.4 Hz, *ortho*-$C_6H_4$), 162.5 (q, $^{1}J_{BC}$ = 49.6 Hz, *ipso*-$C_6H_4$) ppm.

$^{11}$B{$^{1}$H} NMR (128 MHz, THF-$d_8$, 25°C): δ = −7.17 ppm.

Anal. Calc. for $C_{55}H_{88}BN_5$ (830.15g mol$^{-1}$): C, 79.58; H, 10.69; N, 8.44. Found: C, 79.40; H, 10.58; N, 8.68%.

## 2.8 NMR and IR Spectra of [(Me$_5$PACP)H][B(C$_6$H$_4$-4-$^n$Bu)$_4$] (**1b**).

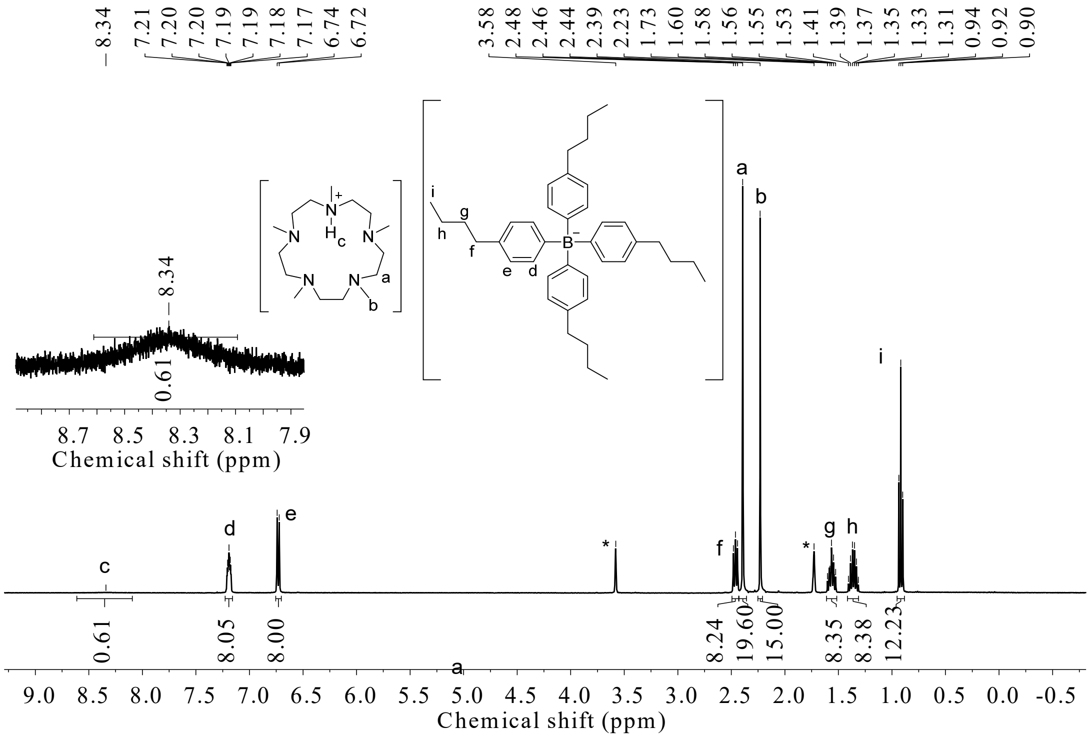


Figure S13: $^1$H NMR spectrum (400 MHz, THF-$d_8$ (*), 25 °C) of [(Me$_5$PACP)H][B(C$_6$H$_4$-4-$^n$Bu)$_4$].

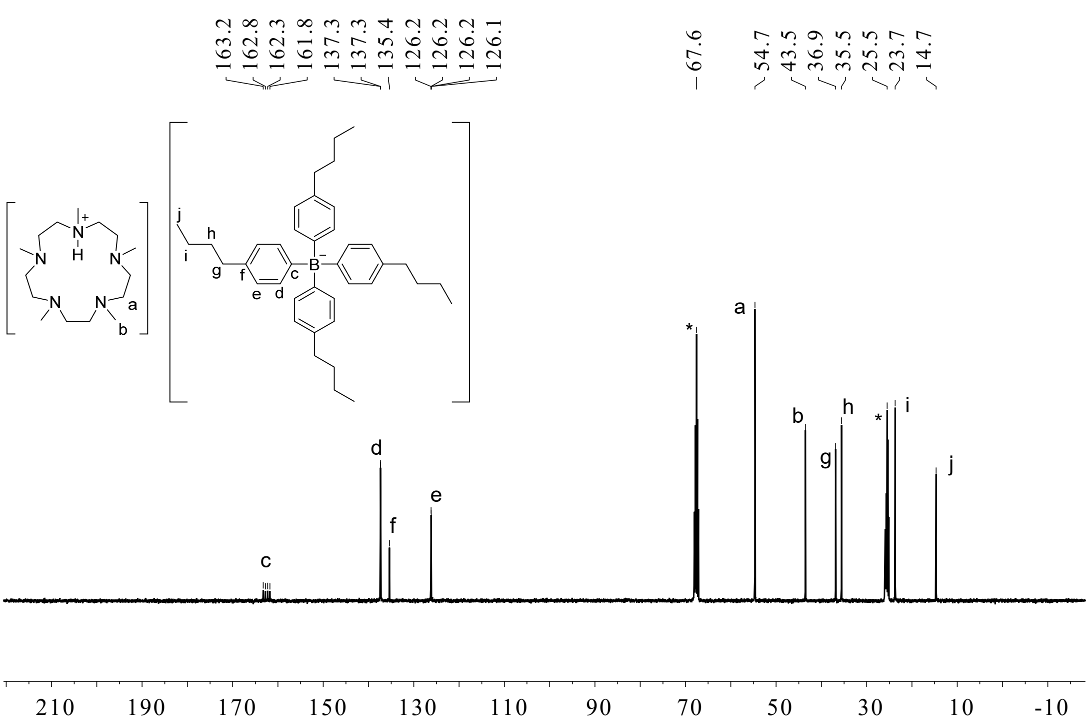


Figure S14: $^{13}$C{$^1$H} NMR spectrum (101 MHz, THF-$d_8$ (*), 25 °C) of [(Me$_5$PACP)H][B(C$_6$H$_4$-4-$^n$Bu)$_4$].

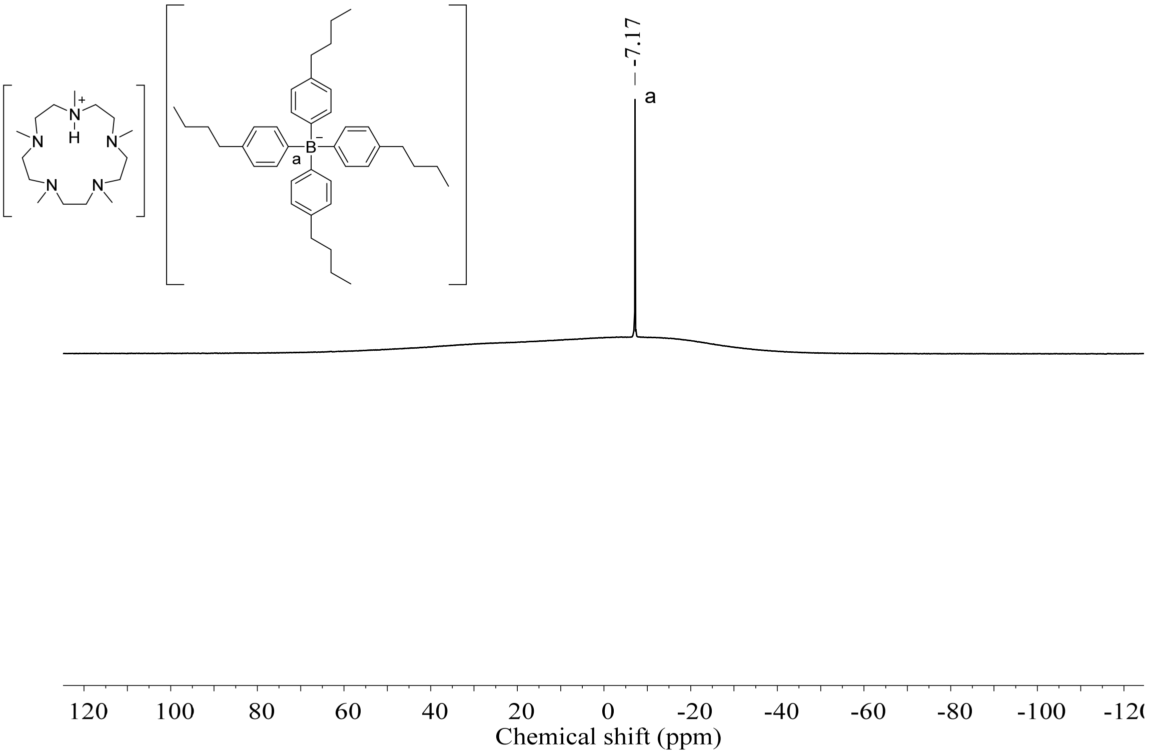


Figure S15: $^{11}B\{^1H\}$ NMR spectrum (128 MHz, THF-$d_8$, 25 °C) of [(Me$_5$PACP)H][B(C$_6$H$_4$-4-$^n$Bu)$_4$].

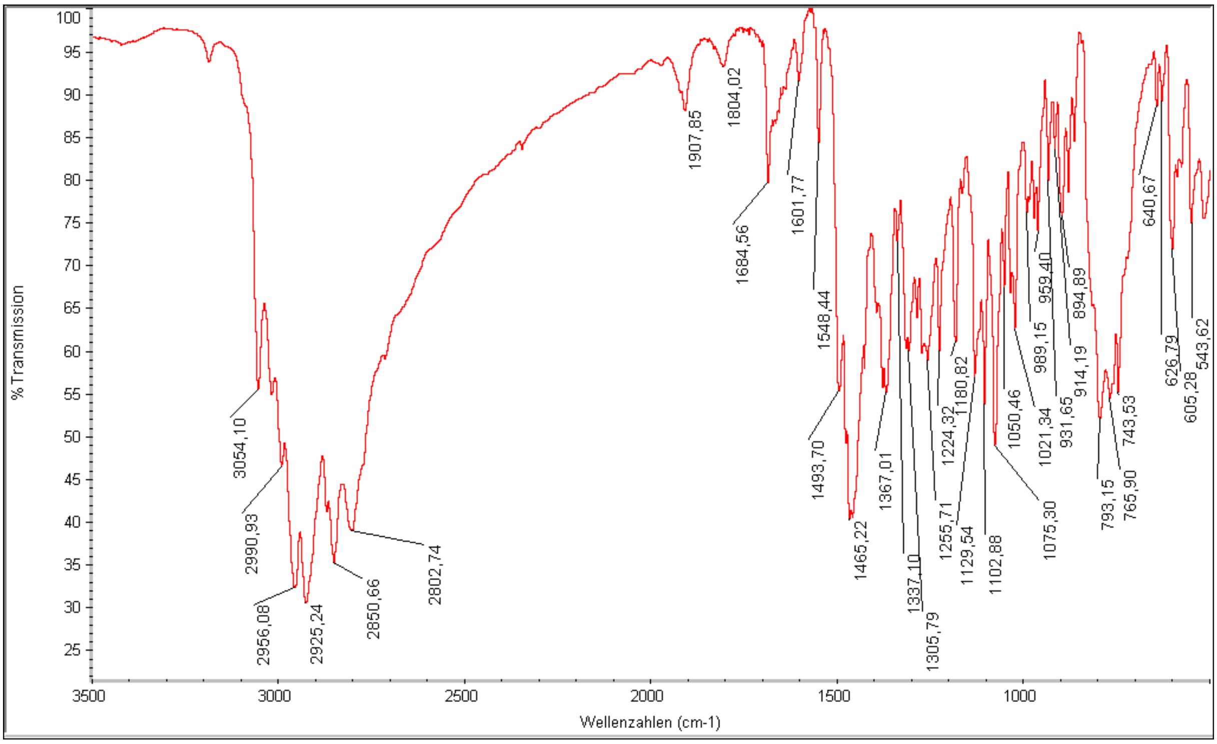


Figure S16: IR spectrum (KBr) of [(Me$_5$PACP)H][B(C$_6$H$_4$-4-$^n$Bu)$_4$].

## 2.9 Molecular Structure of [(Me$_5$PACP)H][B(C$_6$H$_4$-4-$^n$Bu)$_4$] (**1b**).

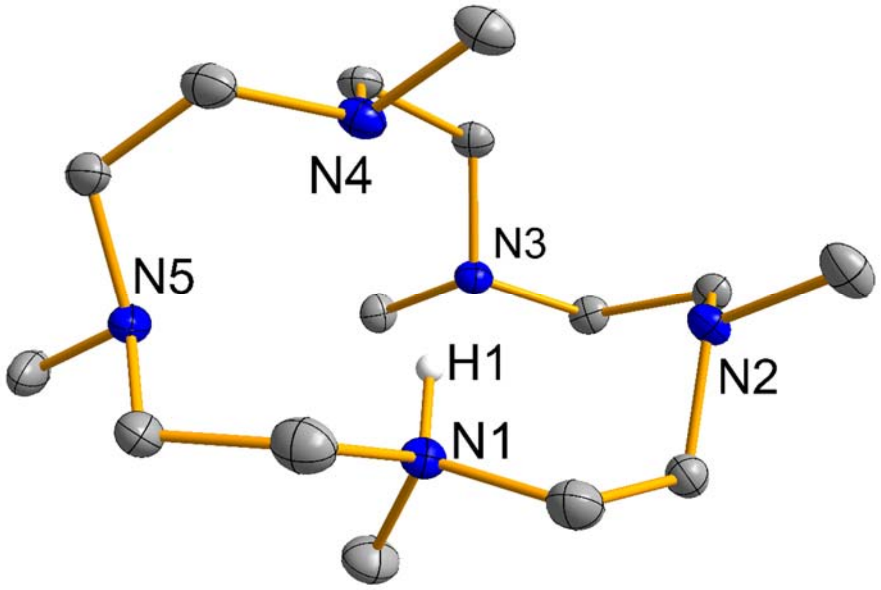


Figure S17: Molecular structure of **1b**. Displacement ellipsoids are shown with 30% probability. The borate anion and all hydrogen atoms apart from N-H are omitted for clarity.

## 2.10 Synthesis of [($Me_5$PACP)Ca($CH_2$Ph)][B($C_6H_3$-3,5-$Me_2$)$_4$] (**2a**).

To a solution of [Ca($CH_2$Ph)$_2$] (22 mg, 0.1 mmol) in THF (3 mL), a solution of [($Me_5$PACP)H][B($C_6H_3$-3,5-$Me_2$)$_4$] (72 mg, 0.1 mmol) in THF (3 mL) was added dropwise at room temperature. The yellow solution was reduced to ca. 3 mL, filtered, layered with n-pentane (2 mL), and stored at –30 °C. After 16 h, yellow crystals formed. The supernatant was decanted off and the crystals were washed with n-pentane (3 x 2 mL). After removing all volatiles *in vacuo*, [($Me_5$PACP)Ca($CH_2$Ph)][B($C_6H_3$-3,5-$Me_2$)$_4$] was isolated as yellow microcrystals (60 mg, 71 µmol); yield: 71%.

$^1$H NMR (400 MHz, THF-$d_8$, 25 °C): δ = 1.48 (s, 2H, C$H_2$Ph), 2.00 − 2.26 (m, 41H, NC$H_2$ + NC$H_3$ + ArC$H_3$), 2.33 − 2.59 (m, 12H, NC$H_2$ + NC$H_3$), 2.65 − 2.79 (m, 6H, NC$H_2$), 6.00 − 6.10 (m, 1 H, *para*-Ph), 6.33 − 6.40 (m, 4 H, *para*-$C_6H_3$), 6.40 − 6.49 (m, 2 H, *ortho*-Ph), 6.62 − 6.72 (m, 2H, meta-Ph), 6.94 − 7.02 (m, 8 H, *ortho*-$C_6H_3$) ppm.

$^{13}$C{$^1$H} NMR (101 MHz, THF-$d_8$, 25 °C): δ = 22.5 (s, Ar$CH_3$), 40.0 (N$CH_3$), 41.5 (s, $CH_2$Ph), 42.1 (s, N$CH_3$), 46.6 (s, N$CH_3$), 55.7 (s, N$CH_2$), 56.8 (s, N$CH_2$), 57.2 (s, N$CH_2$, 57.3 (s, N$CH_2$), 57.9 (s, N$CH_2$), 113.2 (s, *para*-Ph), 120.8 (s, *ortho*-Ph), 123.8(*para*-$C_6H_3$), 129.6 (s, *meta*-Ph), 133.1 (q, $^3J_{BC}$ = 2.7 Hz, *meta*-$C_6H_3$), 135.7 (*ortho*-$C_6H_3$), 159.1 (s, *ipso*-Ph), 165.8 (q, $^1J_{BC}$ = 49.3 Hz, *ipso*-$C_6H_3$) ppm.

$^{11}$B{$^1$H} NMR (128 MHz, THF-$d_8$, 25 °C): δ = –6.97 ppm.

Anal. calc. for $C_{54}H_{78}BN_5Ca$ (847.14 g mol$^{-1}$): C, 76.47; H, 9.27; N, 8.26. Found: C, 74.98; H, 9.27; N, 8.27%.

## 2.11 NMR Spectra of [(Me5PACP)Ca(CH2Ph)][B(C6H3-3,5-Me2)4] (**2a**).

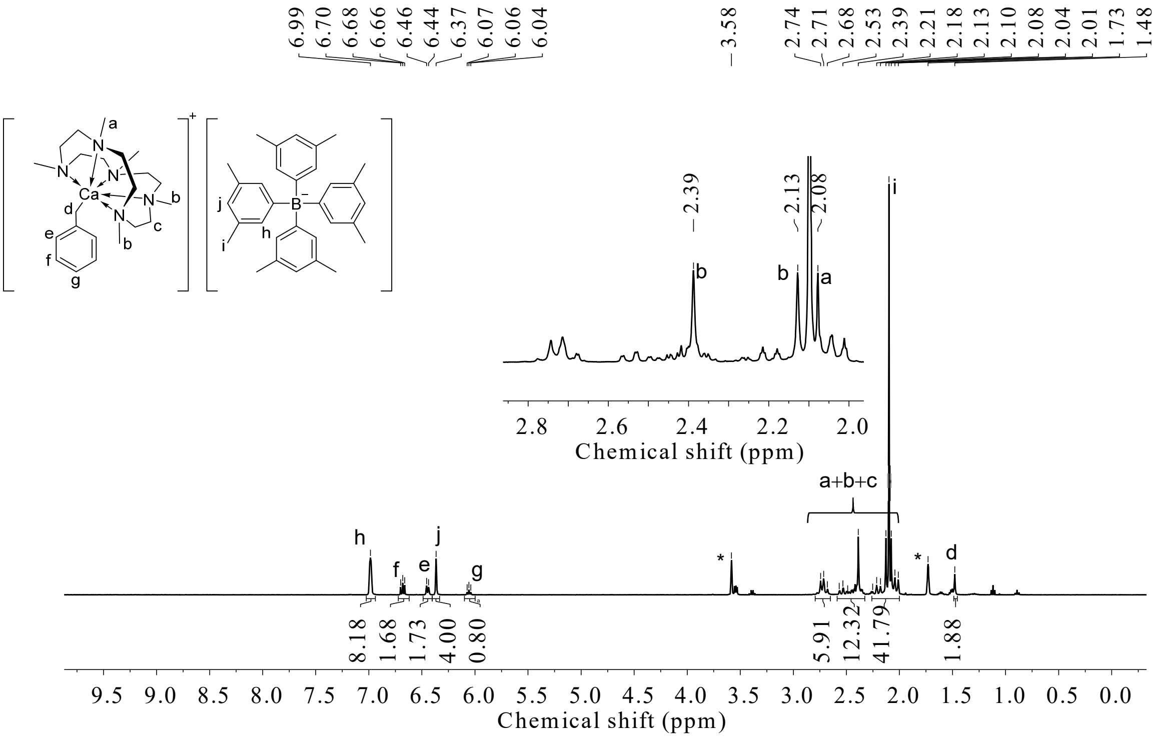


Figure S18: $^{1}$H NMR spectrum (400 MHz, THF-*d8* (*), 25 °C) of [(Me5PACP)Ca(CH2Ph)][B(C6H3-3,5-Me2)4].

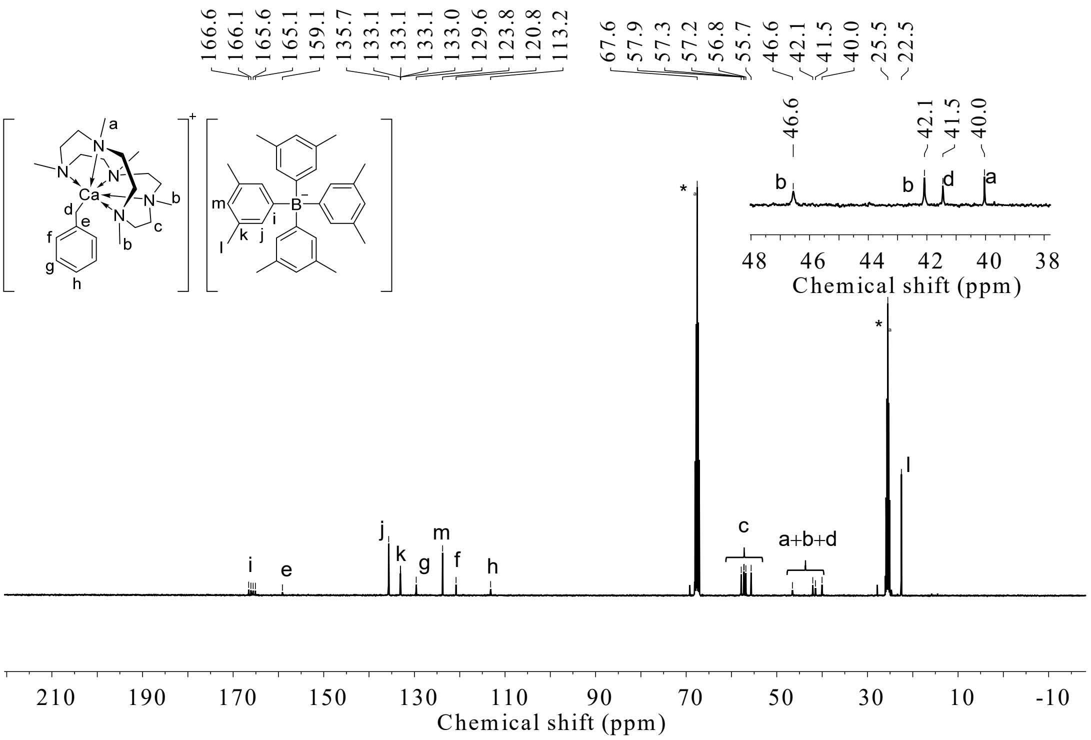


Figure S19: $^{13}$C{$^{1}$H} NMR spectrum (101 MHz, THF-*d8* (*), 25 °C) of [(Me5PACP)Ca(CH2Ph)][B(C6H3-3,5-Me2)4].

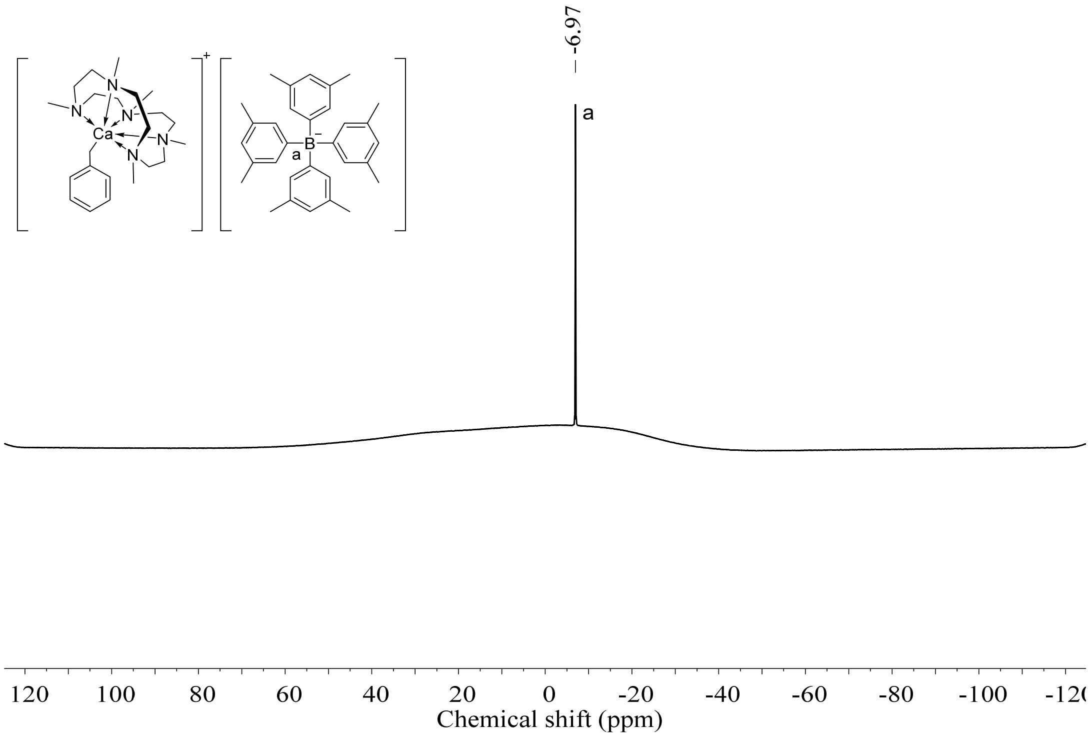


Figure S20: $^{11}B\{^{1}H\}$ NMR spectrum (128 MHz, THF-$d_8$, 25 °C) of [(Me$_5$PACP)Ca(CH$_2$Ph)][B(C$_6$H$_3$-3,5-Me$_2$)$_4$].

## 2.12 Synthesis of $[(Me_5PACP)Ca(CH_2Ph)][B(C_6H_4\text{-}4\text{-}^nBu)_4]$ (**2b**).

To a solution of $[Ca(CH_2Ph)_2]$ (28 mg, 0.12 mmol) in THF (1.5 mL), a solution of $[(Me_5PACP)H][B(C_6H_4\text{-}4\text{-}^nBu)_4]$ (104 mg, 0.12 mmol) in THF (1.5 mL) was added dropwise. The resulting light yellow solution was stirred for 5 min, filtered, layered with n-pentane (3 mL) and stored at −40°C for 16 h. A light yellow waxy solid precipitated, dried and triturated with n-pentane (2 mL) for 30 min. The resulting light yellow powder was washed with n-pentane and dried under reduced pressure to give $[(Me_5PACP)Ca(CH_2Ph)][B(C_6H_4\text{-}4\text{-}^nBu)_4]$ as a light yellow powder (65 mg, 0.07 mmol); yield: 54%.

$^1$H NMR (400 MHz, THF-$d_8$, 25 °C): δ = 0.92 (t, $^3J_{HH}$ = 7.3 Hz, 12H, Ar-$CH_2CH_2CH_2CH_3$), 1.31 − 1.40 (m, 8H, Ar-$CH_2CH_2CH_2CH_3$), 1.49 (s, 2H, $CH_2$Ph), 1.52 − 1.60 (m, 8H, Ar-$CH_2CH_2CH_2CH_3$), 2.04 − 2.20 (m, 13H, N$CH_2$ + N$CH_3$ ), 2.21 − 2.31 (m, 4H, N$CH_2$), 2.35 − 2.53 (m, 18H, Ar-$CH_2CH_2CH_2CH_3$ + N$CH_2$ + N$CH_3$), 2.53 − 2.90 (m, 8H, N$CH_2$), 6.03 – 6.07 (m, 1H, *para*-Ph), 6.44 – 6.46 (m, 2H, *ortho*-Ph), 6.66 – 6.68 (m, 2H, *meta*-Ph), 6.72 (d, $^3J_{HH}$ = 7.7 Hz, 8H, *meta*-$C_6H_4$), 7.17 (dq, $^3J_{HH}$ = 7.7 Hz, $^3J_{BH}$ = 2.7 Hz, 8H, *ortho*-$C_6H_4$) ppm.

$^{13}$C{$^1$H} NMR (101 MHz, THF-$d_8$, 25 °C): δ = 14.7 (s, Ar-$CH_2CH_2CH_2CH_3$), 23.7 (s, Ar-$CH_2CH_2CH_2CH_3$), 35.5 (s, Ar-$CH_2CH_2CH_2CH_3$), 36.9 (s, Ar-$CH_2CH_2CH_2CH_3$), 40.2 (s, N$CH_3$), 41.5 (s, $CH_2$Ph), 42.1 (s, N$CH_3$), 46.5 (s, N$CH_3$), 55.7 (s, N$CH_2$), 56.8 (s, N$CH_2$), 57.2 (s, N$CH_2$, 57.2 (s, N$CH_2$), 57.9 (s, N$CH_2$), 113.1 (s, *para*-Ph), 120.8 (s, *ortho*-Ph), 126.1 (q, $^3J_{BC}$ = 2.8 Hz, *meta*-$C_6H_4$), 129.6 (s, *meta*-Ph), 135.2 (s, *para*-$C_6H_4$), 137.4 (q, $^2J_{BC}$ = 1.4 Hz, *ortho*-$C_6H_4$), 159.1 (s, *ipso*-Ph), 162.6 (q, $^1J_{BC}$ = 49.6 Hz, *ipso*-$C_6H_4$) ppm.

$^{11}$B{$^1$H} NMR (128 MHz, THF-$d_8$, 25°C): δ = −7.13 ppm.

Anal. Calc. for $C_{62}H_{94}BCaN_5$ (960.36g mol$^{-1}$): C, 77.54; H, 9.87; N, 7.29. Found: C, 77.03; H, 10.06; N, 7.24%.

## 2.13 NMR Spectra of [(Me$_5$PACP)Ca(CH$_2$Ph)][B(C$_6$H$_4$-4-$^n$Bu)$_4$] (**2b**).

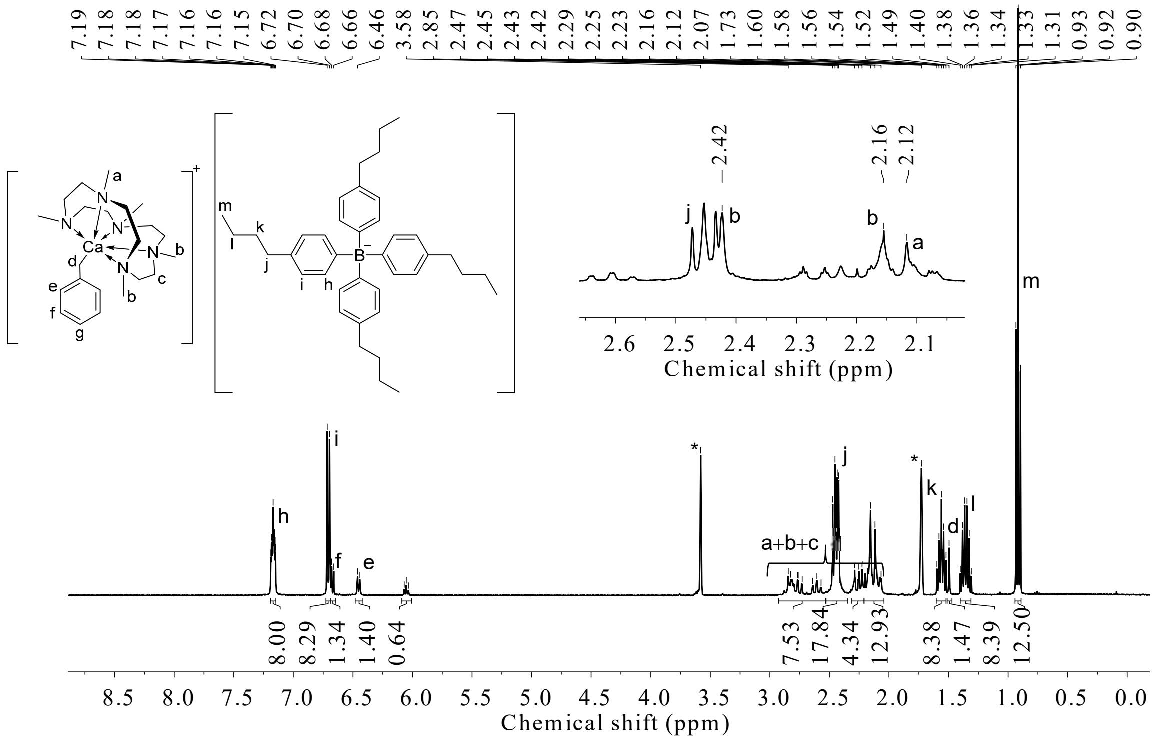


Figure S21: $^1$H NMR spectrum (400 MHz, THF-$d_8$ (*), 25 °C) of [(Me$_5$PACP)Ca(CH$_2$Ph)][B(C$_6$H$_4$-4-$^n$Bu)$_4$].

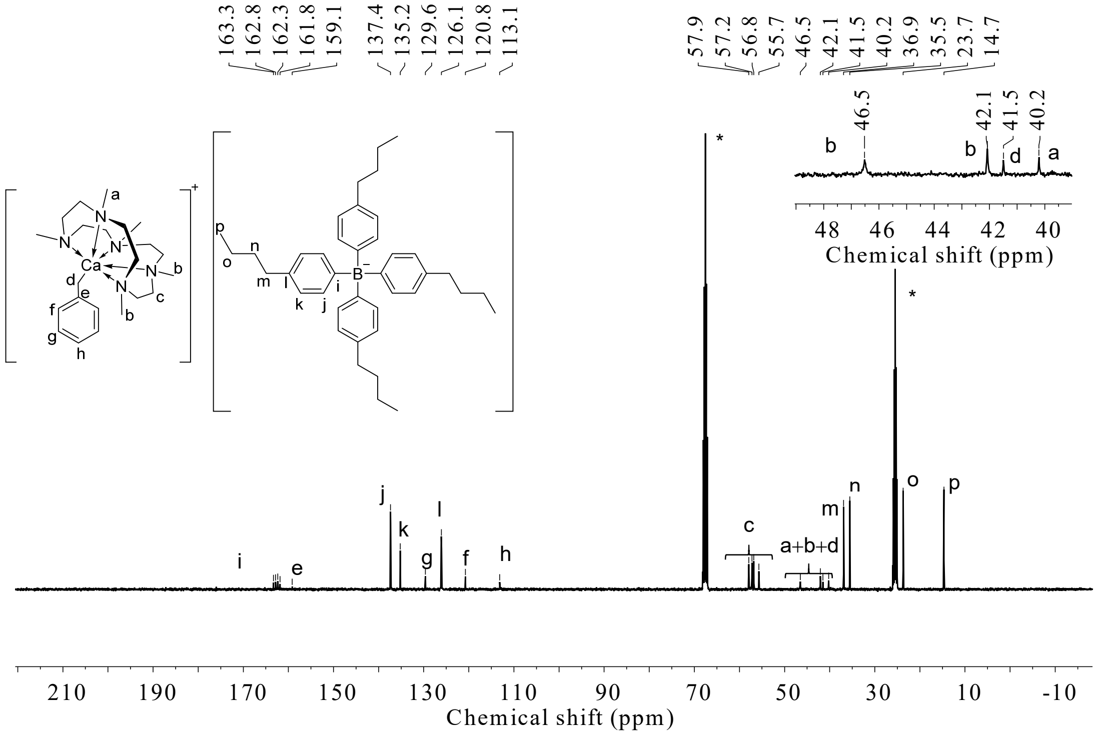


Figure S22: $^{13}$C{$^1$H} NMR spectrum (101 MHz, THF-$d_8$ (*), 25 °C) of [(Me$_5$PACP)Ca(CH$_2$Ph)][B(C$_6$H$_4$-4-$^n$Bu)$_4$.

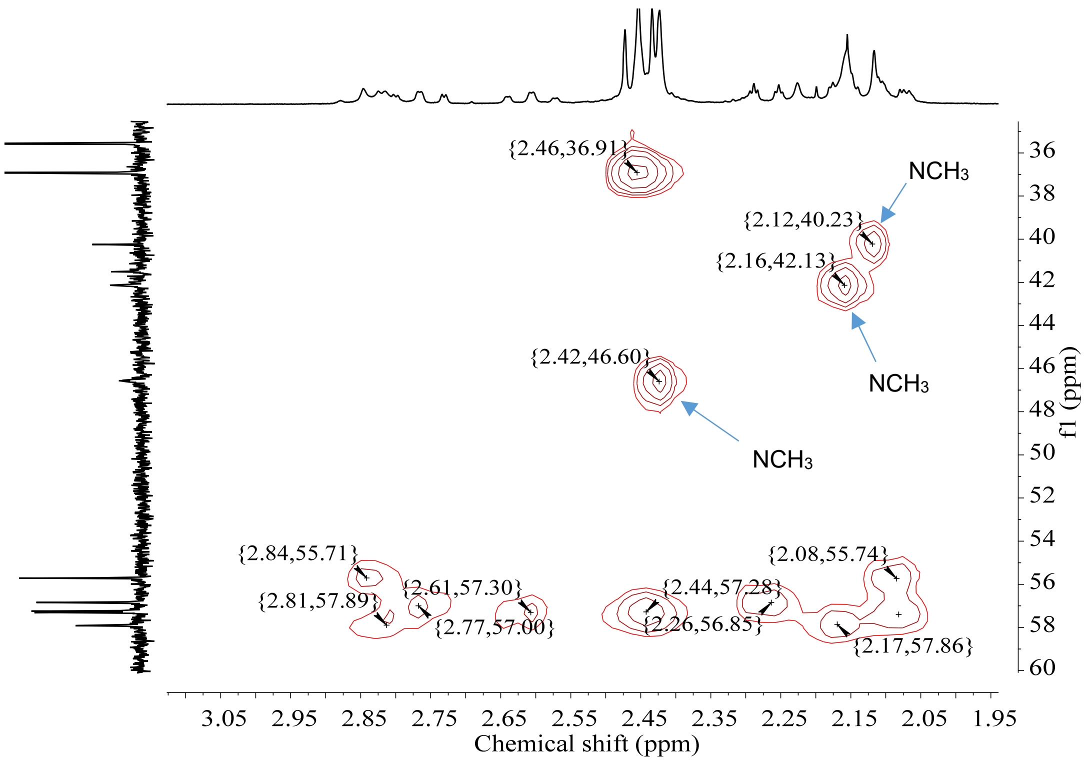


Figure S23: HSQC Spectrum of the ligand region of [(Me$_5$PACP)Ca(CH$_2$Ph)][B(C$_6$H$_4$-4-$^n$Bu)$_4$]. The three distinct resonances for the NCH$_3$ groups are highlighted.

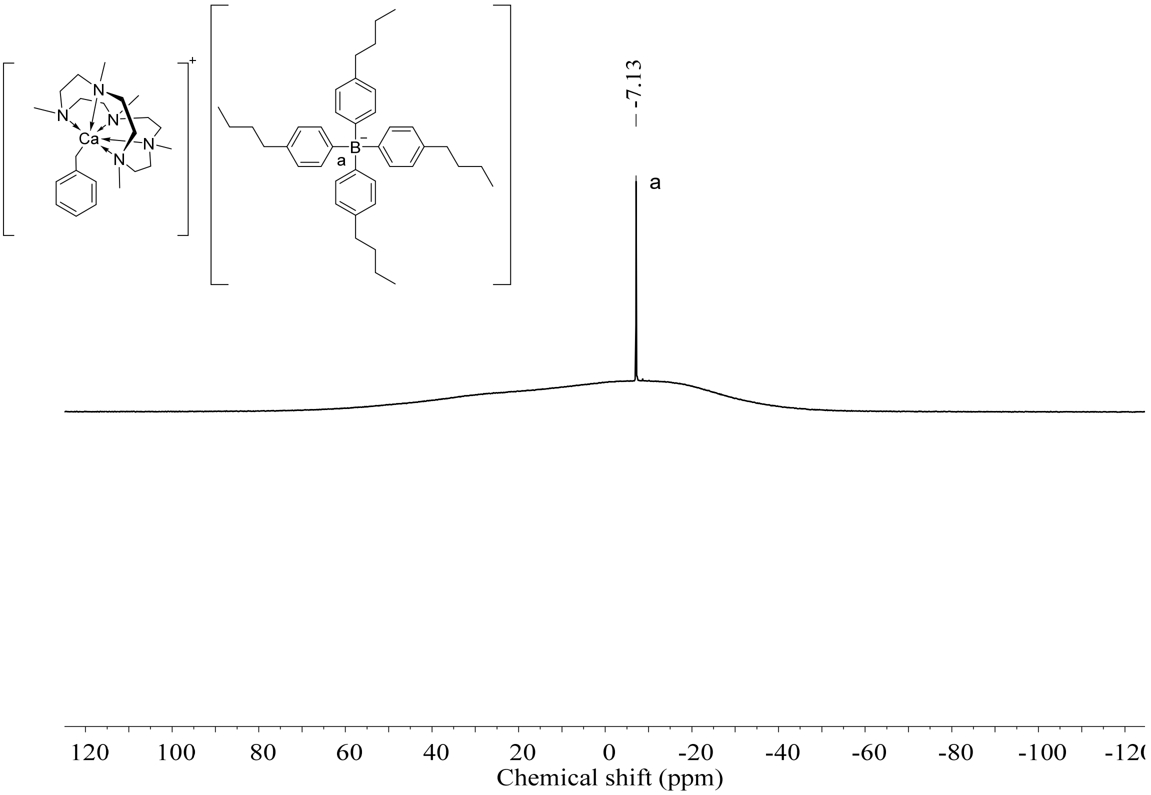


Figure S24: $^{11}B\{^1H\}$ NMR spectrum (128 MHz, THF-$d_8$, 25 °C) of [(Me$_5$PACP)Ca(CH$_2$Ph)][B(C$_6$H$_4$-4-$^n$Bu)$_4$].

## 2.14 Synthesis of $[(Me_5PACP)Ca(\mu\text{-}H)]_2[B(C_6H_3\text{-}3,5\text{-}Me_2)_4]_2$ (**3a**).

In a glass autoclave, a filtered solution of $[(Me_5PACP)Ca(CH_2Ph)][B(C_6H_3\text{-}3,5\text{-}Me_2)_4]$ (30 mg, 35 μmol) in THF (2 mL) was degassed and pressurized with $H_2$. Colorless crystals formed within 16 h. The mother liquor was decanted off, the colorless crystals were washed with cold THF (2 mL) and n-pentane (2 x 2 mL) and dried under vacuum to give $[(Me_5PACP)Ca(\mu\text{-}H)]_2[B(C_6H_3\text{-}3,5\text{-}Me_2)_4]_2$ (19 mg, 11 μmol); yield: 63%. Insolubility in aliphatic and aromatic hydrocarbons as well as in ethereal solvents precluded recording of NMR spectra. Crystals isolated were suitable for X-ray diffraction analysis.

Anal. calc. for $C_{94}H_{144}B_2N_{10}Ca_2$ (1516.03 g $mol^{-1}$): C, 74.47; H, 9.57; N, 9.24. Found: C, 74.21; H, 9.53; N, 8.16%.

## 2.15 Synthesis of $[(Me_5PACP)_2Ca_2(\mu\text{-}H)_2][B(C_6H_4\text{-}4\text{-}^nBu)_4]_2$ and $[(Me_5PACP)_2Ca_2(\mu\text{-}D)_2][B(C_6H_4\text{-}4\text{-}^nBu)_4]_2$. (**3b/ 3b-*d*₂**).

Method A: $[Ca(CH_2Ph)_2]$ (44 mg, 0.20 mmol) and $[(Me_5PACP)H][B(C_6H_4\text{-}4\text{-}^nBu)_4]$ (166 mg, 0.20 mmol) were combined in benzene (5 mL) and stirred for 5 min. The resulting suspension was degassed, charged with $H_2$ (1 bar) and stirred for 16 h during which a colorless precipitate formed. The precipitate was isolated, washed with n-pentane (3 x 2 mL) and recrystallized from THF/n-pentane to give colorless crystals. The crystals were isolated, washed with n-pentane (3 x 2 mL) and carefully dried under reduced pressure to give $[(Me_5PACP)_2Ca_2(\mu\text{-}H)_2][B(C_6H_4\text{-}4\text{-}^nBu)_4]_2$ as colorless crystals (139 mg, 80 µmol); yield: 80%.

$[(Me_5PACP)_2Ca_2(\mu\text{-}D)_2][B(C_6H_4\text{-}4\text{-}^nBu)_4]_2$ was synthesized according to Method A using $D_2$ (1 bar) on a 0.1 mmol scale and isolated as colorless crystals (70 mg, 37 µmol); yield: 80%.

Method B: $[Ca(CH_2Ph)_2]$ (44 mg, 0.20 mmol) and $[(Me_5PACP)H][B(C_6H_4\text{-}4\text{-}^nBu)_4]$ (166 mg, 0.20 mmol) were combined in benzene (5 mL) and stirred for 5 min. Octylsilane (62 mg, 0.43 mmol) was added dropwise and the reaction mixture stirred for 2 h during which a colorless precipitate formed. The precipitate was isolated, washed with n-pentane (3 x 2 mL) and recrystallized from THF/n-pentane to give colorless crystals. The crystals were isolated, washed with n-pentane (3 x 2 mL) and carefully dried under reduced pressure to give $[(Me_5PACP)_2Ca_2(\mu\text{-}H)_2][B(C_6H_4\text{-}4\text{-}^nBu)_4]_2$ as colorless crystals (143 mg, 82 µmol); yield: 82%.

Crystals grown from THF/n-pentane were suitable for X-ray diffraction analysis.

$^1H$ NMR (400 MHz, THF-$d_8$, 25 °C): δ = 0.92 (t, $^3J_{HH}$ = 7.3 Hz, 12H, Ar-$CH_2CH_2CH_2CH_3$), 1.31 − 1.40 (m, 8H, Ar-$CH_2CH_2CH_2CH_3$), 1.52 − 1.60 (m, 8H, Ar-$CH_2CH_2CH_2CH_3$), 1.92 − 2.33 (m, 34H, N$CH_2$ + N$CH_3$ ), 2.37 − 2.54 (m, 36H, Ar-$CH_2CH_2CH_2CH_3$ + N$CH_2$ + N$CH_3$), 2.56 − 2.98 (m, 16H, N$CH_2$), 4.63 (s, 2H, Ca*H*), 6.72 (d, $^3J_{HH}$ = 7.7 Hz, 8H, *meta*-$C_6H_4$), 7.17 (dq, $^3J_{HH}$ = 7.7 Hz, $^3J_{BH}$ = 2.7 Hz, 8H, *ortho*-$C_6H_4$) ppm.

$^2H$ NMR (61 MHz, THF-$d_8$, 25 °C): δ = 4.72 (s, Ca*D*) ppm.

$^{13}C\{^1H\}$ NMR (101 MHz, THF-$d_8$, 25 °C): δ = 14.6 (s, Ar-$CH_2CH_2CH_2CH_3$), 23.6 (s, Ar-$CH_2CH_2CH_2CH_3$), 35.4 (s, Ar-$CH_2CH_2CH_2CH_3$), 36.8 (s, Ar-$CH_2CH_2CH_2CH_3$), 41.0 (s, N$CH_3$), 43.6 (s, N$CH_3$), 48.4 (s, N$CH_3$), 55.5 (s, N$CH_2$), 56.8 (s, N$CH_2$), 57.5 (s, N$CH_2$, 57.6 (s, N$CH_2$), 58.3 (s, N$CH_2$), 125.9 (q, $^3J_{BC}$ = 2.8 Hz, *meta*-$C_6H_4$), 134.9 (s, *para*-$C_6H_4$), 137.3 (q, $^2J_{BC}$ = 1.4 Hz, *ortho*-$C_6H_4$), 162.6 (q, $^1J_{BC}$ = 49.6 Hz, *ipso*-$C_6H_4$) ppm.

$^{11}B\{^1H\}$ NMR (128 MHz, THF-$d_8$, 25°C): δ = −7.13 ppm.

Anal. Calc. for $C_{110}H_{176}B_2Ca_2N_{10}$ (1740.46g $mol^{-1}$): C, 75.91; H, 10.19; N, 8.05. Found: C, 74.82; H, 9.99; N, 7.87%.

## 2.16 NMR Spectra of $[(Me_5PACP)_2Ca_2(\mu\text{-}H)_2][B(C_6H_4\text{-}4\text{-}^nBu)_4]_2$ and $[(Me_5PACP)_2Ca_2(\mu\text{-}D)_2][B(C_6H_4\text{-}4\text{-}^nBu)_4]_2$ (**3b/ 3b-*d*₂**)

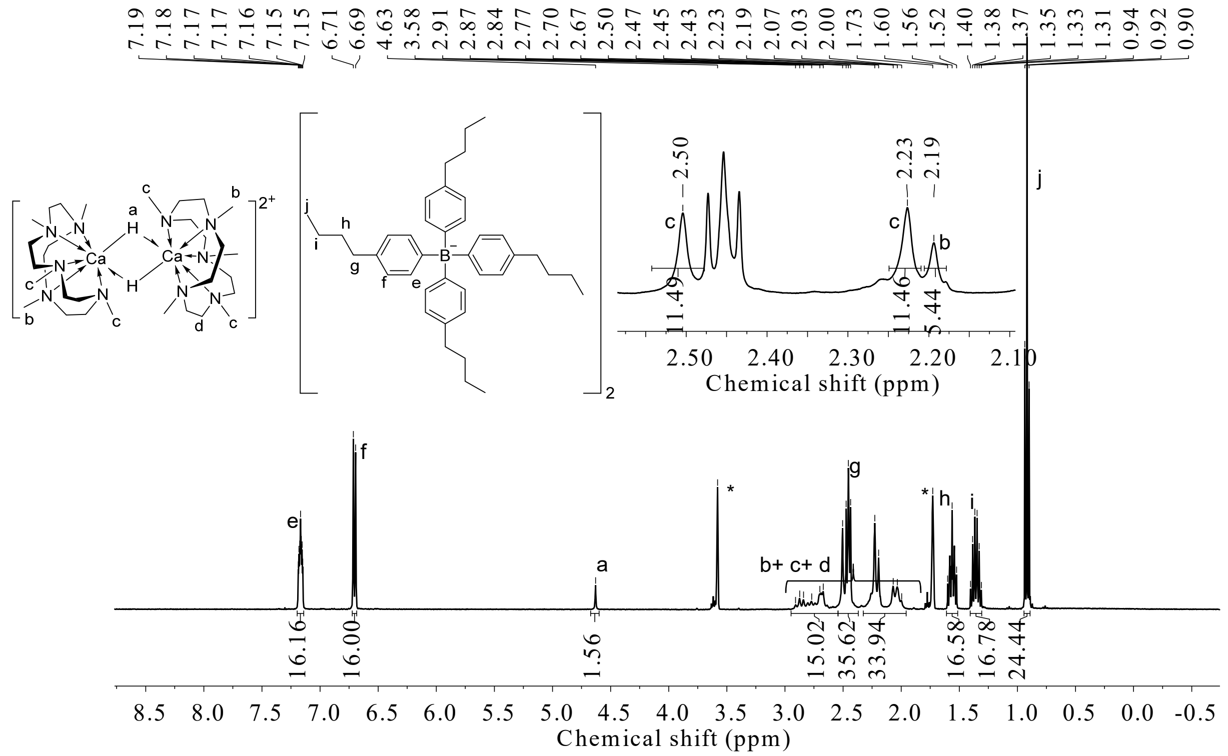


Figure S25: $^1$H NMR spectrum (400 MHz, THF-$d_8$ (*), 25 °C) of $[(Me_5PACP)_2Ca_2(\mu\text{-}H)_2][B(C_6H_4\text{-}4\text{-}^nBu)_4]_2$.

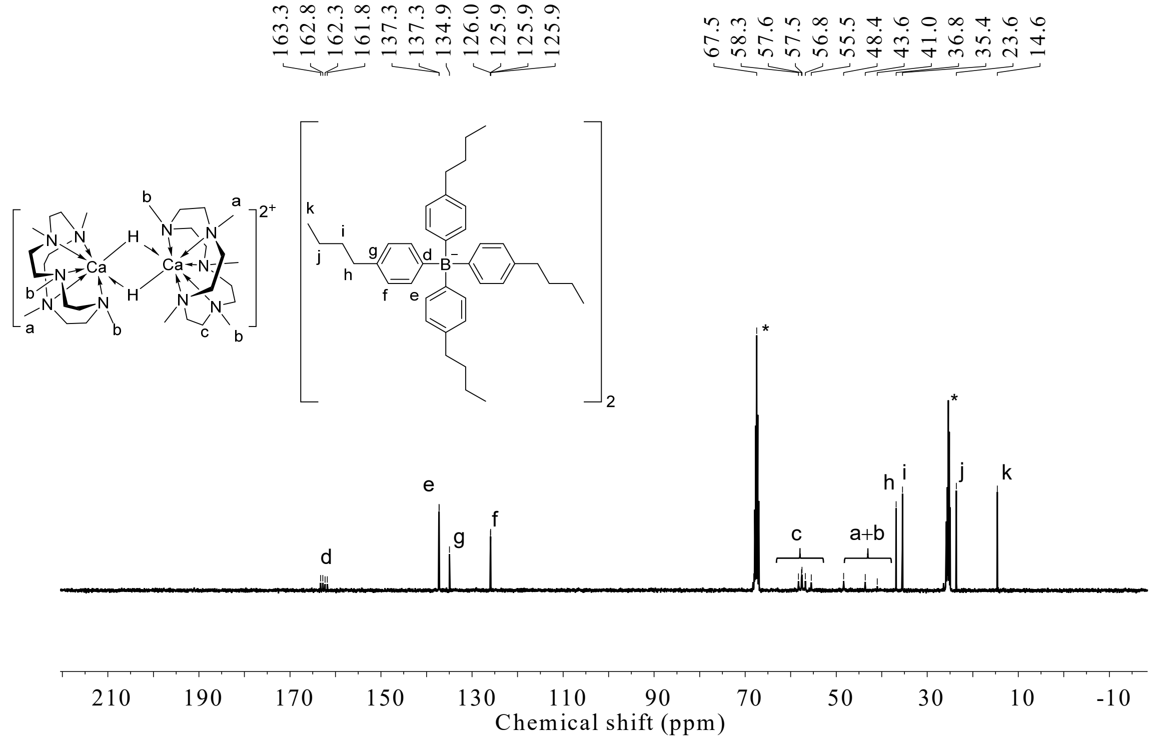


Figure S26: $^{13}C\{^1H\}$ NMR spectrum (101 MHz, THF-$d_8$ (*), 25 °C) of $[(Me_5PACP)_2Ca_2(\mu\text{-}H)_2][B(C_6H_4\text{-}4\text{-}^nBu)_4]_2$.

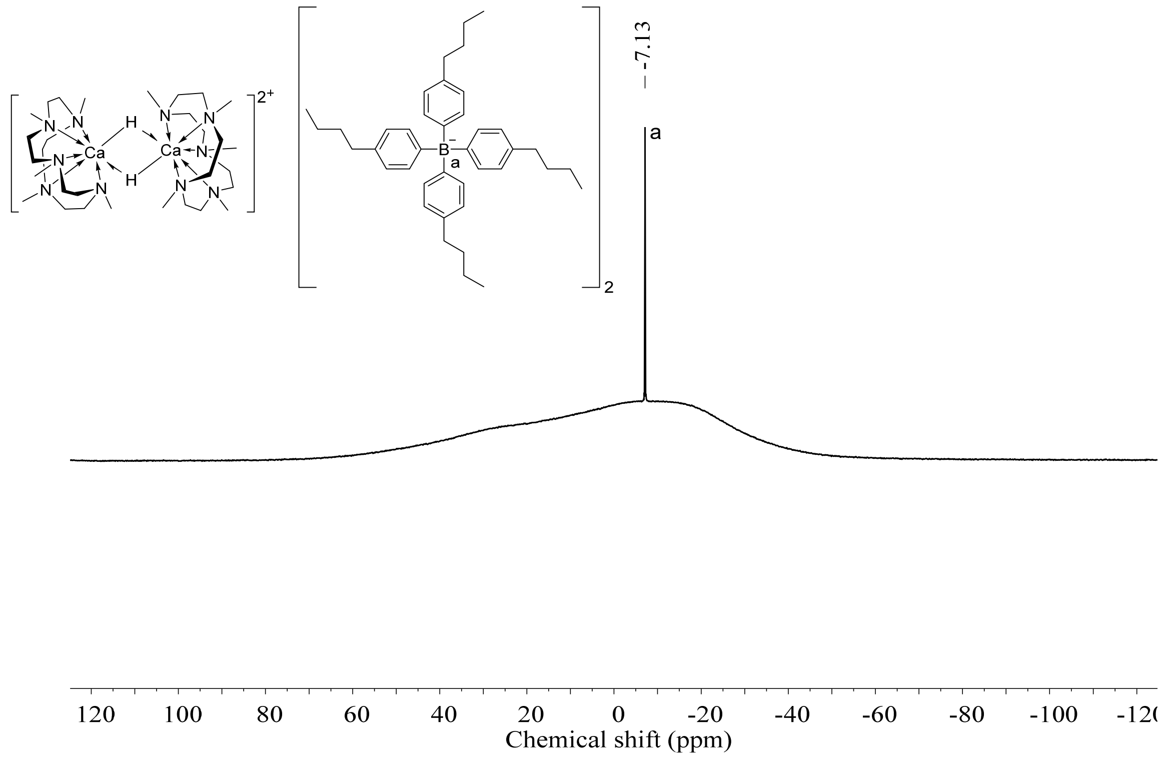


Figure S27: $^{11}B\{^{1}H\}$ NMR spectrum (128 MHz, THF-$d_8$, 25 °C) of $[(Me_5PACP)_2Ca_2(\mu\text{-}H)_2][B(C_6H_4\text{-}4\text{-}^{n}Bu)_4]_2$.

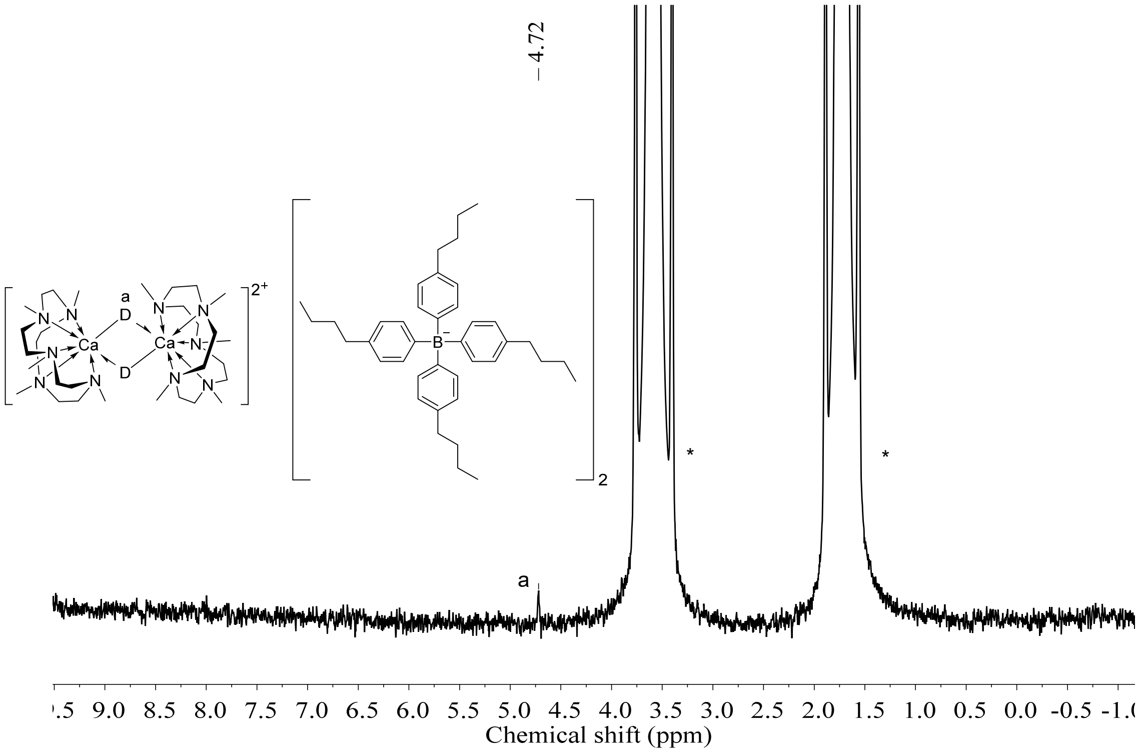


Figure S28: $^{2}H$ NMR spectrum (61 MHz, THF-$d_8$ (*), 25 °C) of $[(Me_5PACP)_2Ca_2(\mu\text{-}D)_2][B(C_6H_4\text{-}4\text{-}^{n}Bu)_4]_2$.

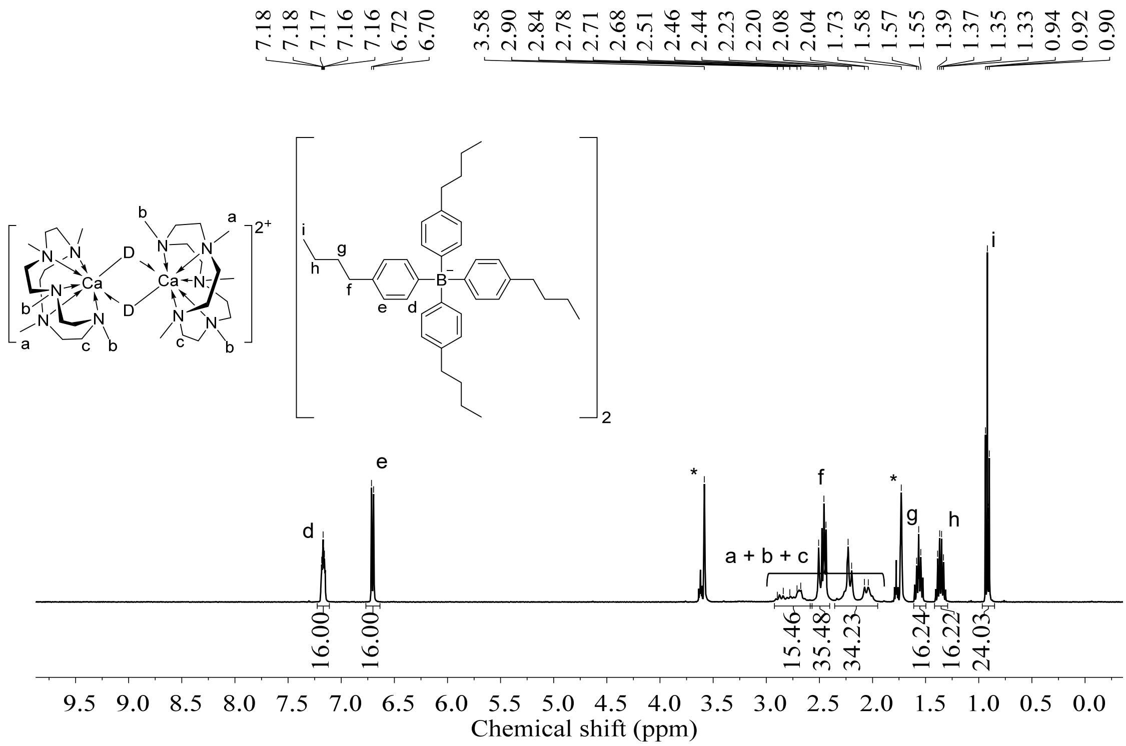


Figure S29: $^{1}H$ NMR spectrum (400 MHz, THF-$d_8$ (*), 25 °C) of $[(Me_5PACP)_2Ca_2(\mu\text{-}D)_2][B(C_6H_4\text{-}4\text{-}^nBu)_4]_2$.

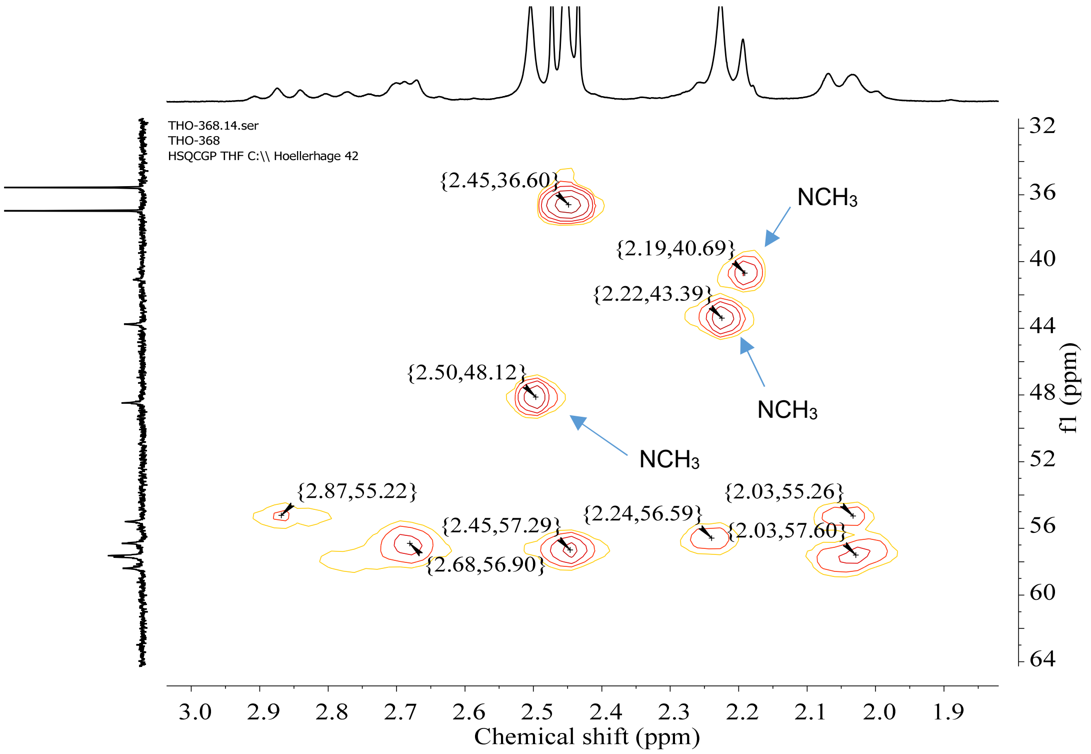


Figure S30: HSQC Spectrum of the ligand region of $[(Me_5PACP)_2Ca_2(\mu\text{-}H)_2][B(C_6H_4\text{-}4\text{-}^nBu)_4]_2$. The three distinct resonances for the $NCH_3$ groups are highlighted.

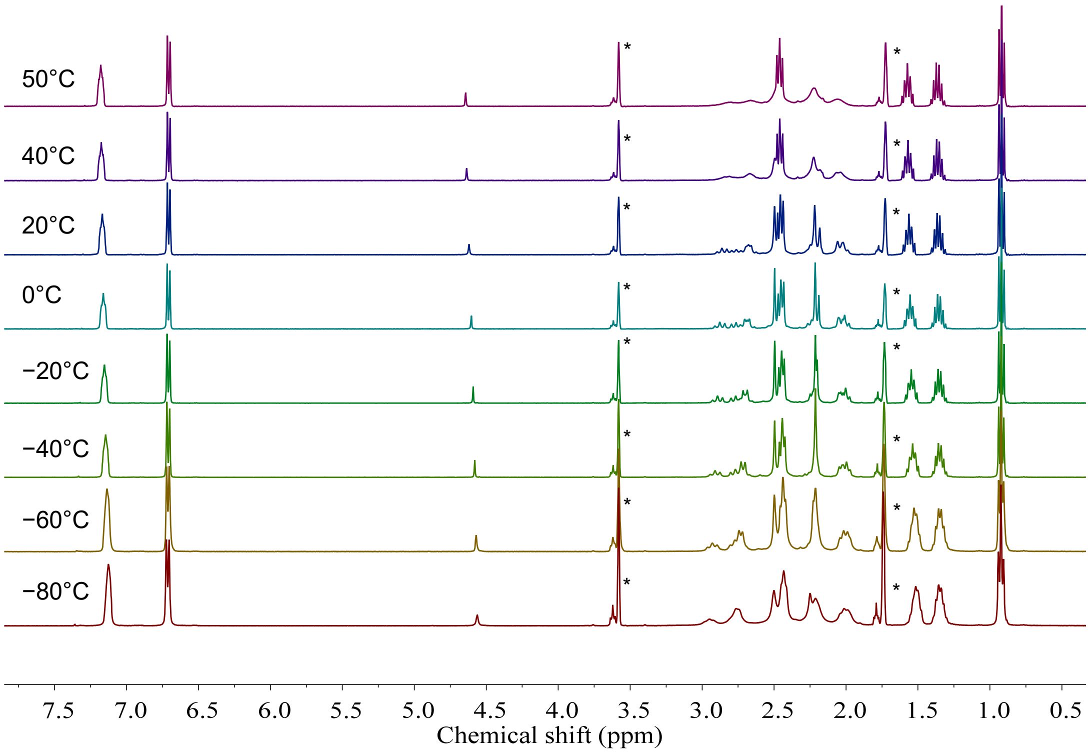


Figure S31: Varible-temperature $^{1}H$ NMR spectra of **3b** in THF-$d_8$ (*).

## 2.17 Molecular Structure of $[(Me_5PACP)_2Ca_2(\mu\text{-}H)_2][B(C_6H_4\text{-}4\text{-}^nBu)_4]_2$ (**3b**).

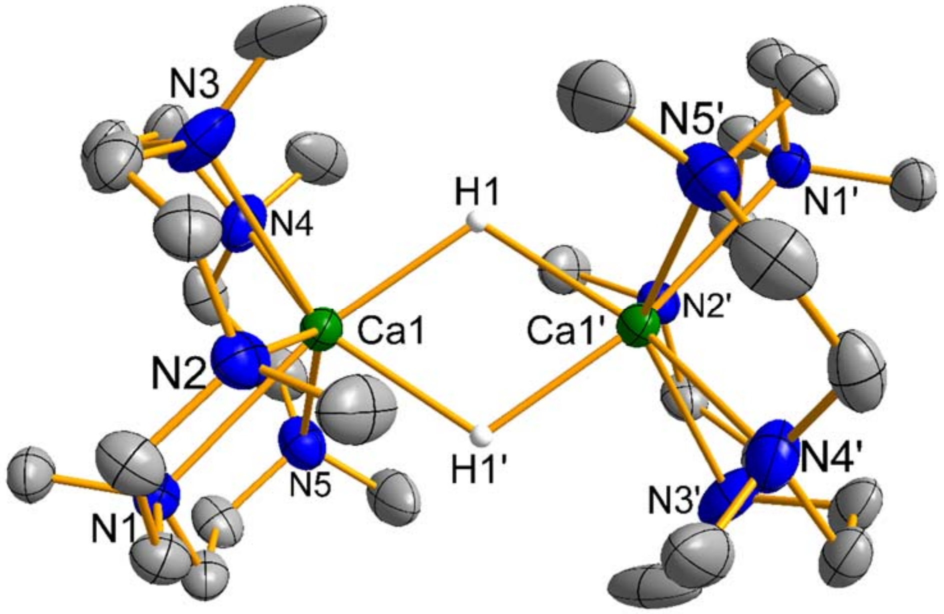


Figure S32: Molecular structure of the dication of **3b**. Thermal ellipsoids are set at 30% probability; the anions and the hydrogen atoms except for the hydride ligands are omitted for clarity. Selected interatomic distances [Å]: Ca1···Ca1' 3.7178(10), Ca1–H1 2.24(2), Ca1–H1' 2.27(2), Ca1–N1 2.689(2), Ca1–N2 2.694(2), Ca1–N3 2.633(3), Ca1–N4 2.619(3), Ca1–N5 2.754(2) Å.

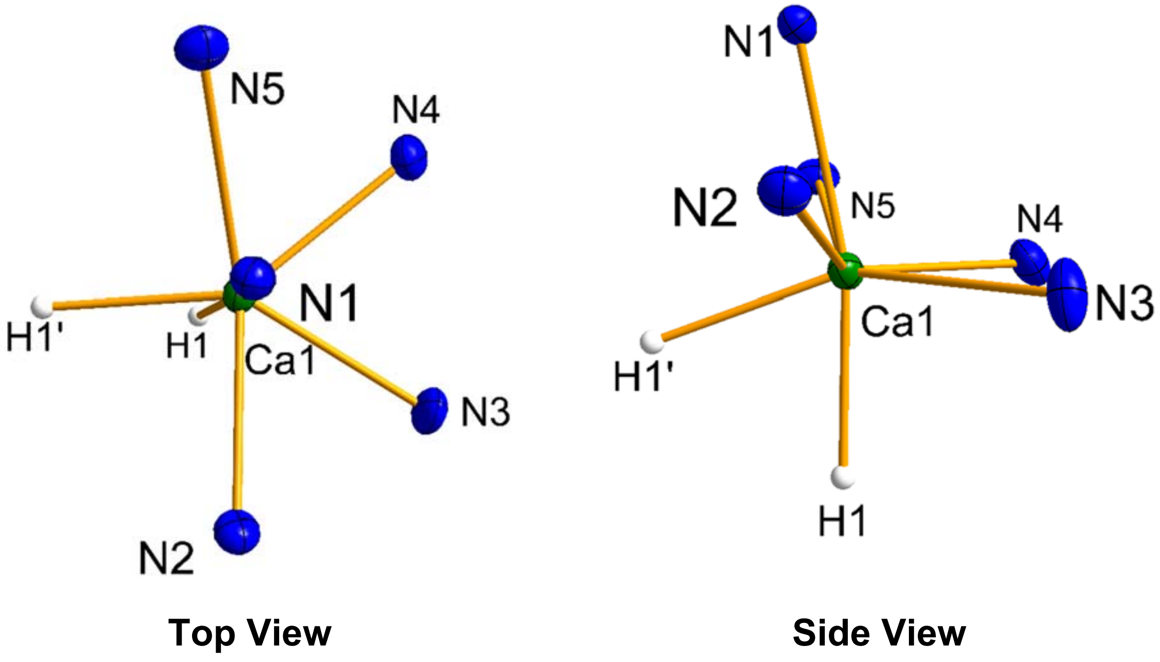


Figure S33: Coordination sphere of calcium in **3b**. Left: Top view of the pentagonal bipyramid with H1 and N1 in apical positions. Right: Side view of the pentagonal bipyramid. H1' and N2 to N5 lie in the plane of the (distorted) pentagonal bipyramid.

## 2.18 Synthesis of [($Me_4$TACD)Ca($CH_2$Ph)][B($C_6H_4$-4-$^n$Bu)$_4$] (**5**).

To a solution of [Ca($CH_2$Ph)$_2$] (33 mg, 0.15 mmol) in THF (1.5 mL) was added dropwise a solution of [($Me_4$TACD)H][B($C_6H_4$-4-$^n$Bu)$_4$] (116 mg, 0.15 mmol) in THF (1.5 mL). The resulting orange reaction mixture was stirred for 5 min, filtered, layered with n-pentane (3 mL) and stored at −40 °C for 16 h. A red waxy solid precipitated, was isolated by decanting off the supernatant, and dried. The solid was triturated with $Et_2O$ (1 mL) for 30 min and the resulting red powder was washed with n-pentane (2 x 2 mL) and dried under reduced pressure to give [($Me_4$TACD)Ca($CH_2$Ph)][B($C_6H_4$-4-$^n$Bu)$_4$] as a red powder (75 mg, 0.07 mmol); yield: 55%.

$^1$H NMR (400 MHz, THF-$d_8$, 25 °C): δ = 0.92 (t, $^3J_{HH}$ = 7.3 Hz, 12H, Ar-$CH_2CH_2CH_2C\mathit{H}_3$), 1.31 − 1.41 (m, 8H, Ar-$CH_2CH_2C\mathit{H}_2CH_3$), 1.53 − 1.64 (m, 8H, Ar-$CH_2C\mathit{H}_2CH_2CH_3$), 1.64 (s, 2H, $C\mathit{H}_2$Ph), 2.10 − 2.42 (br, 8H, $NC\mathit{H}_2$), 2.29 (s, 12H, $NC\mathit{H}_3$), 2.46 (t, $^3J_{HH}$ = 7.8 Hz, 8H, Ar-$C\mathit{H}_2CH_2CH_2CH_3$), 2.49 – 2.71 (br, 8H, $NC\mathit{H}_2$), 5.84 – 5.87 (m, 1H, *para*-Ph), 6.21 – 6.23 (m, 2H, *ortho*-Ph), 6.62 – 6.67 (m, 2H, *meta*-Ph), 6.72 (d, $^3J_{HH}$ = 7.7 Hz, 8H, *meta*-$C_6H_4$), 7.17 (dq, $^3J_{HH}$ = 7.7 Hz, $^3J_{BH}$ = 2.7 Hz, 8H, *ortho*-$C_6H_4$) ppm.

$^{13}$C{$^1$H} NMR (101 MHz, THF-$d_8$, 25 °C): δ = 14.7 (s, Ar-$CH_2CH_2CH_2\mathit{C}H_3$), 23.7 (s, Ar-$CH_2CH_2\mathit{C}H_2CH_3$), 35.5 (s, Ar-$CH_2\mathit{C}H_2CH_2CH_3$), 36.9 (s, Ar-$\mathit{C}H_2CH_2CH_2CH_3$), 45.2 (s, $N\mathit{C}H_3$), 54.5 (s, $N\mathit{C}H_2$), 46.0 (s, $\mathit{C}H_2$Ph), 109.3 (s, *para*-Ph), 118.1 (s, *ortho*-Ph), 126.1 (q, $^3J_{BC}$ = 2.8 Hz, *meta*-$C_6H_4$), 130.9 (s, *meta*-Ph), 135.2 (s, *para*-$C_6H_4$), 137.3 (q, $^2J_{BC}$ = 1.4 Hz, *ortho*-$C_6H_4$), 157.8 (s, *ipso*-Ph), 162.6 (q, $^1J_{BC}$ = 49.6 Hz, *ipso*-$C_6H_4$) ppm.

$^{11}$B{$^1$H} NMR (128 MHz, THF-$d_8$, 25°C): δ = −7.13 ppm.

Anal. Calc. for $C_{59}H_{87}BCaN_4$ (903.26g mol$^{-1}$): C, 78.45; H, 9.71; N, 6.20. Found: C, 77.83; H, 9.66; N, 5.97%.

## 2.19 NMR spectra of $[(Me_4TACD)Ca(CH_2Ph)][B(C_6H_4\text{-}4\text{-}^nBu)_4]$ (**5**).

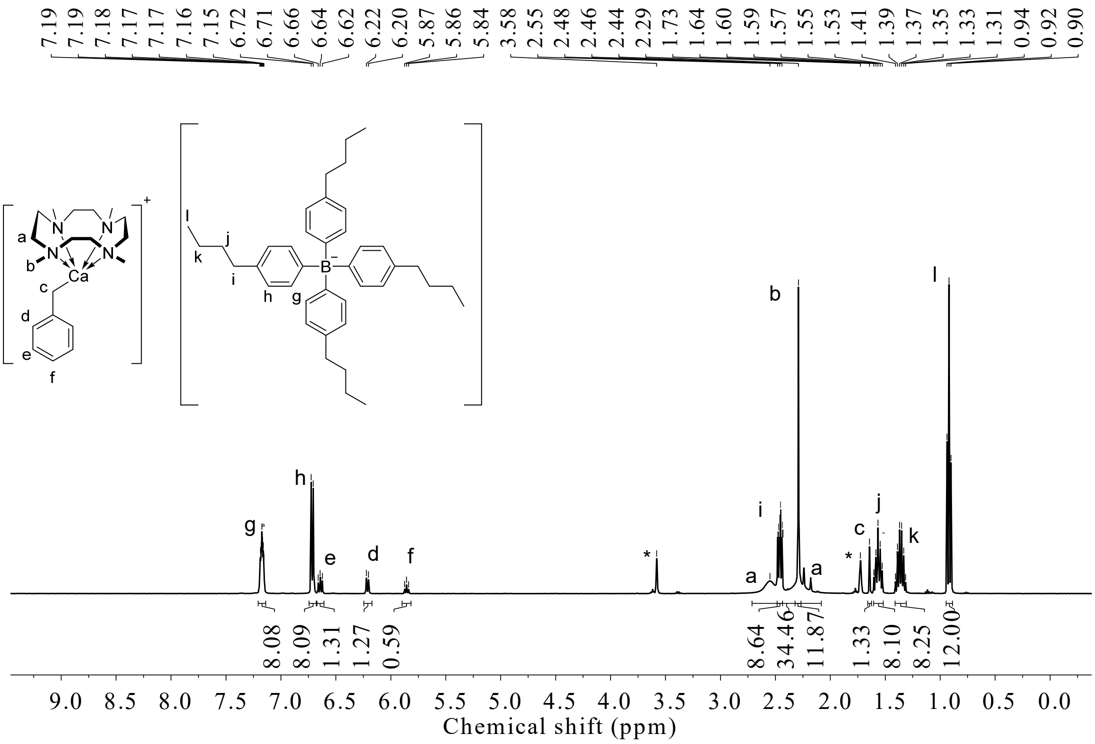


Figure S34: $^1$H NMR spectrum (400 MHz, THF-$d_8$ (*), 25 °C) of $[(Me_4TACD)Ca(CH_2Ph)][B(C_6H_4\text{-}4\text{-}^nBu)_4]$.

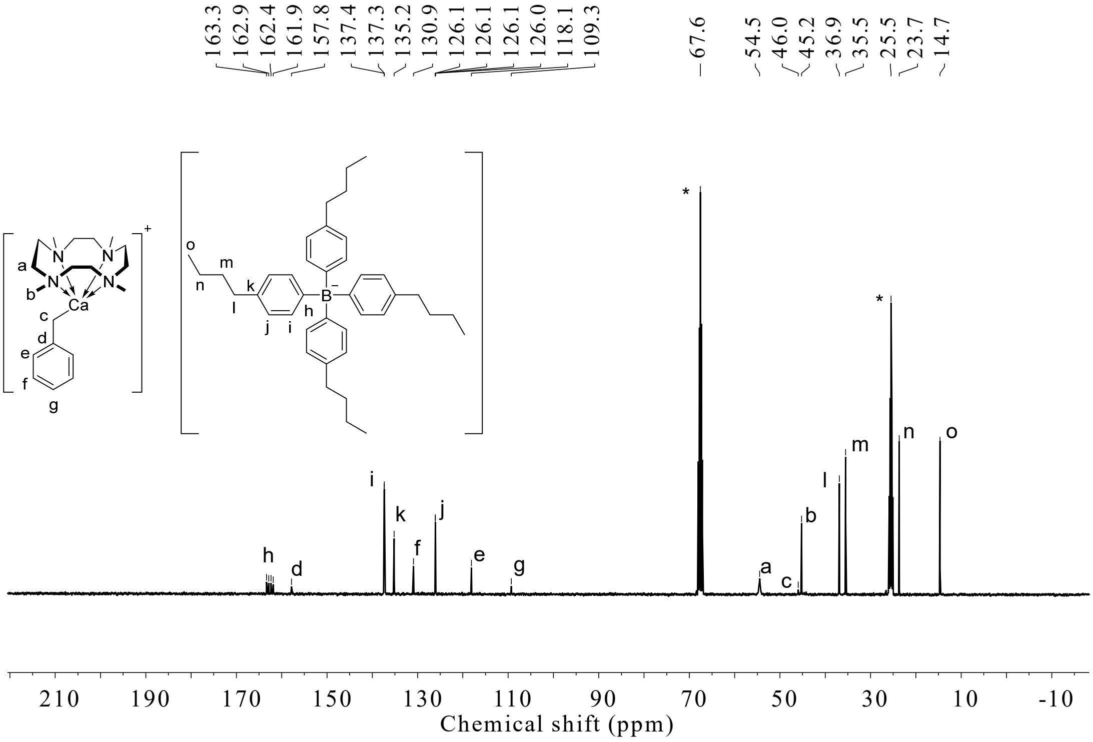


Figure S35: $^{13}C\{^1H\}$ NMR spectrum (101 MHz, THF-$d_8$ (*), 25 °C) of $[(Me_4TACD)Ca(CH_2Ph)][B(C_6H_4\text{-}4\text{-}^nBu)_4]$.

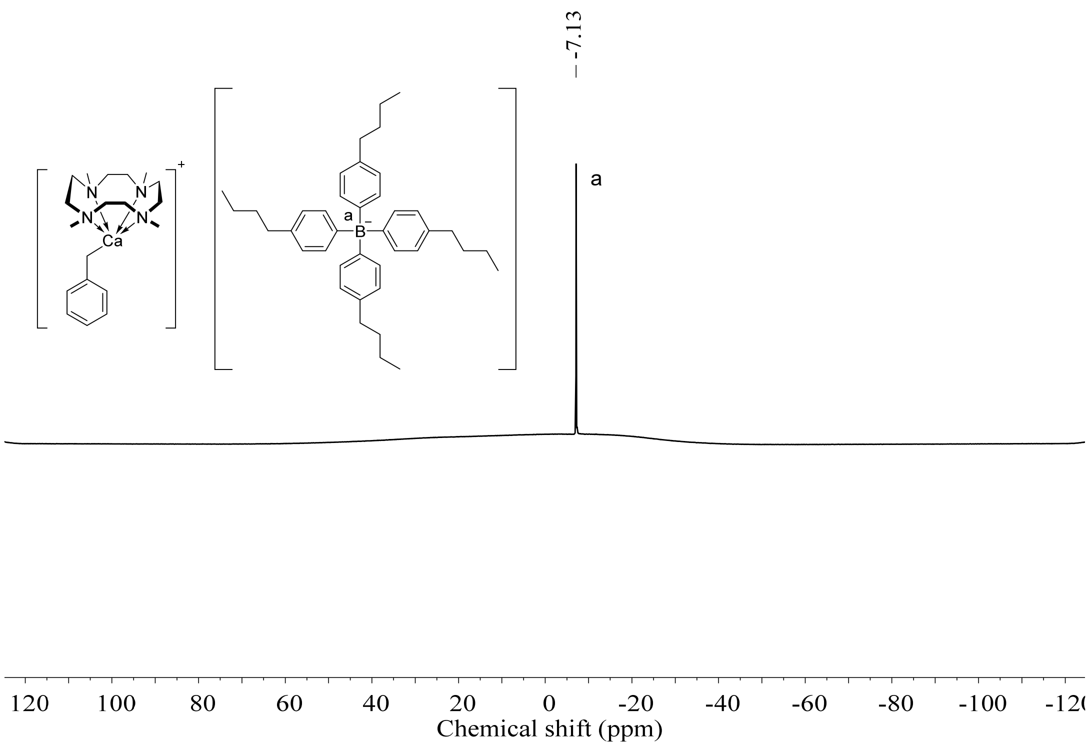


Figure S36: $^{11}B\{^{1}H\}$ NMR spectrum (128 MHz, THF-$d_8$, 25 °C) of $[(Me_4TACD)Ca(CH_2Ph)][B(C_6H_4\text{-}4\text{-}^nBu)_4]$.

## 2.20 Synthesis of $[(Me_4TACD)_2Ca_2(\mu\text{-}H)_2(thf)][B(C_6H_4\text{-}4\text{-}^nBu)_4]_2$ (**6**).

Method A: $[Ca(CH_2Ph)_2]$ (44 mg, 0.20 mmol) and $[(Me_4TACD)H][B(C_6H_4\text{-}4\text{-}^nBu)_4]$ (155 mg, 0.20 mmol) were combined in benzene (4 mL) and stirred for 5 min. The resulting suspension was degassed, charged with $H_2$ (1 bar) and stirred for 16 h during which a colorless precipitate formed. The precipitate was isolated, washed with n-pentane (3 x 2 mL) and recrystallized from THF/n-pentane to give colorless crystals. The crystals were isolated, washed with n-pentane (3 x 2 mL) and carefully dried under reduced pressure. $[(Me_4TACD)_2Ca_2(\mu\text{-}H)_2(thf)][B(C_6H_4\text{-}4\text{-}^nBu)_4]_2$ was isolated as colorless crystals (125 mg, 74 µmol); yield: 74%.

Method B: $[Ca(CH_2Ph)_2]$ (44 mg, 0.20 mmol) and $[(Me_4TACD)H][B(C_6H_4\text{-}4\text{-}^nBu)_4]$ (155 mg, 0.20 mmol) were combined in benzene (4 mL) and stirred for 5 min. n-Octylsilane (60 mg, 0.42 mmol) was added dropwise and the reaction mixture stirred for 2 h during which a colorless precipitate formed. The precipitate was isolated, washed with n-pentane (3 x 2mL) and recrystallized from THF/n-pentane to give colorless crystals. The crystals were isolated, washed with n-pentane (3 x 2 mL) and carefully dried under reduced pressure to give $[(Me_4TACD)_2Ca_2(\mu\text{-}H)_2(thf)][B(C_6H_4\text{-}4\text{-}^nBu)_4]_2$ as colorless crystals (136 mg, 80 µmol); yield: 80%.

Crystals grown from THF/n-pentane were suitable for X-ray diffraction analysis.

$^1H$ NMR (400 MHz, THF-$d_8$, 25 °C): δ = 0.92 (t, $^3J_{HH}$ = 7.3 Hz, 24H, Ar-$CH_2CH_2CH_2CH_3$), 1.30 − 1.41 (m, 8H, Ar-$CH_2CH_2CH_2CH_3$), 1.51 − 1.61 (m, 16H, Ar-$CH_2CH_2CH_2CH_3$), 1.74 – 1.80 (m, 4H, THF), 2.16 − 2.33 (br, 16H, $NCH_2$), 2.37 (s, 24H, $NCH_3$), 2.46 (t, $^3J_{HH}$ = 7.8 Hz, 16H, Ar-$CH_2CH_2CH_2CH_3$), 2.50 − 2.64 (br, 16H, $NCH_2$), 3.59 – 3.65 (m, 4H, THF), 4.51 (s, 2H, Ca*H*), 6.71 (d, $^3J_{HH}$ = 7.7 Hz, 16H, *meta*-$C_6H_4$), 7.17 (m, 16H, *ortho*-$C_6H_4$) ppm.

$^{13}C\{^1H\}$ NMR (101 MHz, THF-$d_8$, 25 °C): δ = 14.6 (s, Ar-$CH_2CH_2CH_2CH_3$), 23.7 (s, Ar-$CH_2CH_2CH_2CH_3$), 35.4 (s, Ar-$CH_2CH_2CH_2CH_3$), 36.8 (s, Ar-$CH_2CH_2CH_2CH_3$), 45.1 (s, $NCH_3$), 54.4 (s, $NCH_2$), 125.9 (q, $^3J_{BC}$ = 2.8 Hz, *meta*-$C_6H_4$), 135.0 (s, *para*-$C_6H_4$), 137.2 (q, $^2J_{BC}$ = 1.4 Hz, *ortho*-$C_6H_4$), 162.6 (q, $^1J_{BC}$ = 49.6 Hz, *ipso*-$C_6H_4$) ppm.

$^{11}B\{^1H\}$ NMR (128 MHz, THF-$d_8$, 25°C): δ = −7.13 ppm.

Anal. Calc. for $C_{108}H_{170}B_2Ca_2N_8O$ (1698.38g $mol^{-1}$): C, 76.38; H, 10.09; N, 6.60. Found: C, 75.64; H, 9.88; N, 6.67%.

## 2.21 NMR Spectra of $[(Me_4TACD)_2Ca_2(\mu\text{-}H)_2(thf)][B(C_6H_4\text{-}4\text{-}^nBu)_4]_2$ (**6**).

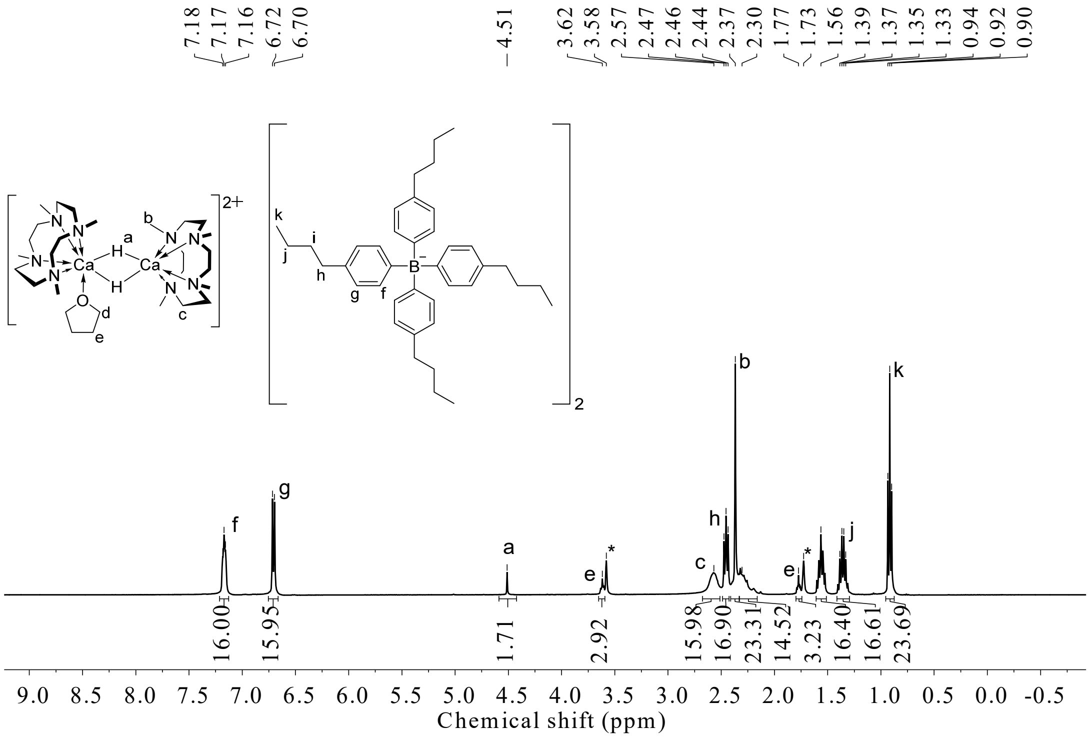


Figure S37: $^1H$ NMR spectrum (400 MHz, THF-$d_8$ (*), 25 °C) of $[(Me_4TACD)_2Ca_2(\mu\text{-}H)_2(thf)][B(C_6H_4\text{-}4\text{-}^nBu)_4]_2$.

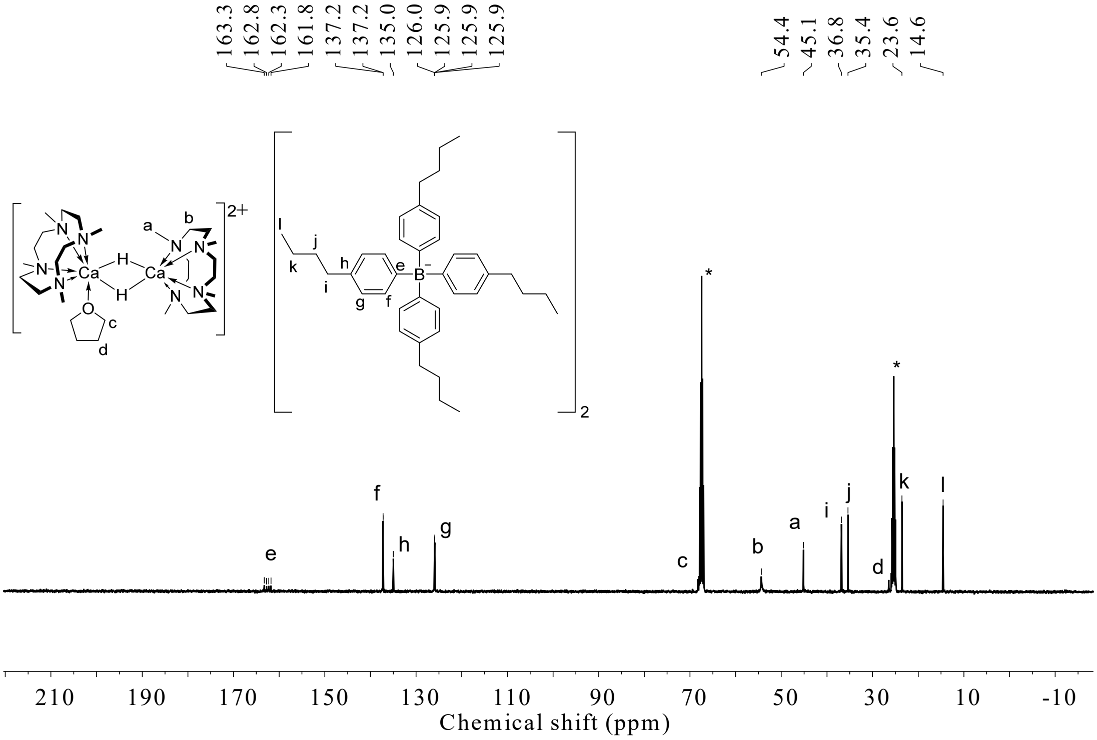


Figure S38: $^{13}C\{^1H\}$ NMR spectrum (101 MHz, THF-$d_8$ (*), 25 °C) of $[(Me_4TACD)_2Ca_2(\mu\text{-}H)_2(thf)][B(C_6H_4\text{-}4\text{-}^nBu)_4]_2$.

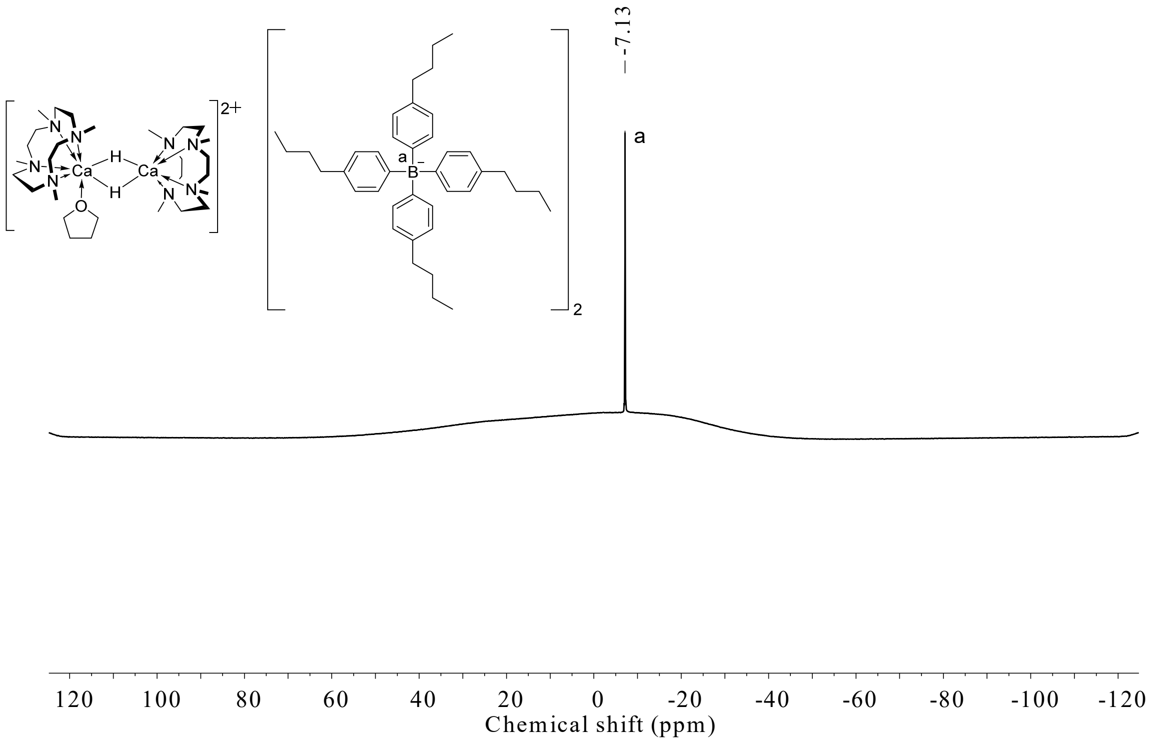


Figure S39: $^{11}B\{^{1}H\}$ NMR spectrum (128 MHz, THF-$d_8$, 25 °C) of $[(Me_4TACD)_2Ca_2(\mu\text{-}H)_2(thf)][B(C_6H_4\text{-}4\text{-}^{n}Bu)_4]_2$.

## 2.22 Molecular Structure of $[(Me_4TACD)_2Ca_2(\mu\text{-}H)_2(thf)][B(C_6H_4\text{-}4\text{-}^{n}Bu)_4]_2$ (**6**).

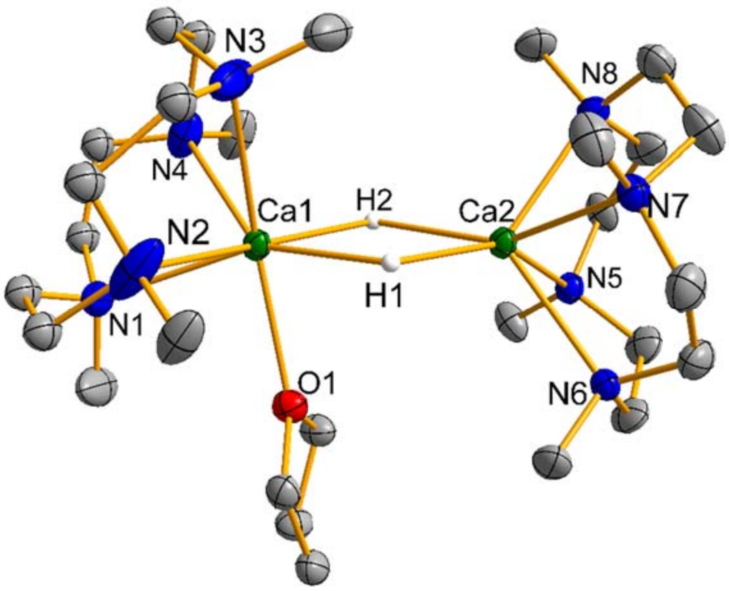


Figure S40: Molecular structure of the cation of **5**. Displacement parameters are set at 30% probability. H atoms apart of Ca-H are omitted for clarity.

# 3 Reactivity Studies and Kinetic Investigations

## 3.1 Stoichiometric Reactions

### 3.1.1 Reaction with $D_2$

In a J. Young-type NMR tube, $[(Me_5PACP)_2Ca_2(\mu\text{-}H)_2][B(C_6H_4\text{-}4\text{-}^nBu)_4]_2$ (5 mg, 3 µmol) was dissolved in 0.5 mL THF-$d_8$. The mixture was degassed applying three “freeze-pump-thaw” cycles and charged with $D_2$ (1 bar). Progress of the reaction was monitored by $^1H$ NMR spectroscopy.

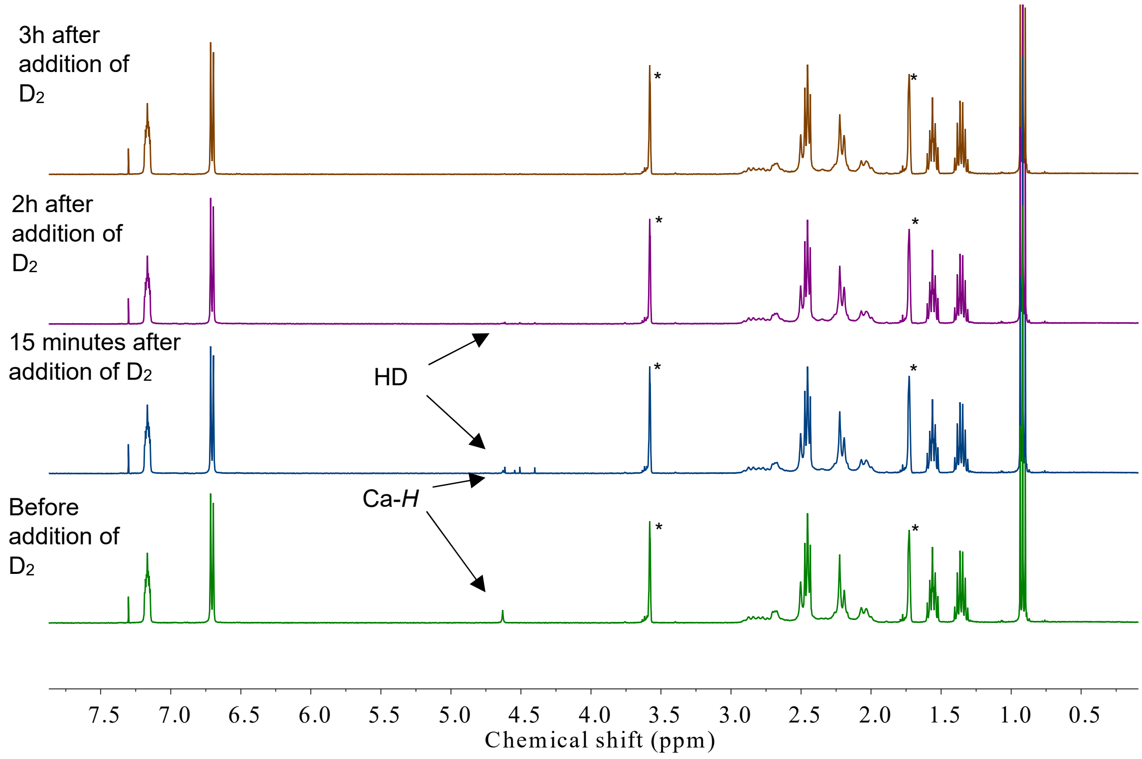


Figure S41: Stacked $^1H$ NMR spectra of the reaction of **3b** with $D_2$ (1bar) in THF-$d_8$ (*) at 25 °C.

3.1.2 Reaction with Ethylene

In a J. Young-type NMR tube, $[(Me_5PACP)_2Ca_2(\mu\text{-}H)_2][B(C_6H_4\text{-}4\text{-}{}^nBu)_4]_2$ (10 mg, 6 μmol) or $[(Me_5PACP)_2Ca_2(\mu\text{-}D)_2][B(C_6H_4\text{-}4\text{-}{}^nBu)_4]_2$ (10 mg, 6 μmol) was dissolved in 0.5 mL of THF-*d*8. The mixture was degassed applying three "freeze-pump-thaw" cycles and charged with ethylene (1 bar) at 0 °C. The in-situ generated ethyl complex was analyzed by $^1$H NMR spectroscopy. For the $^{13}$C NMR measurement, the sample was put into a precooled (0 °C) NMR spectrometer and measured at −20 °C.

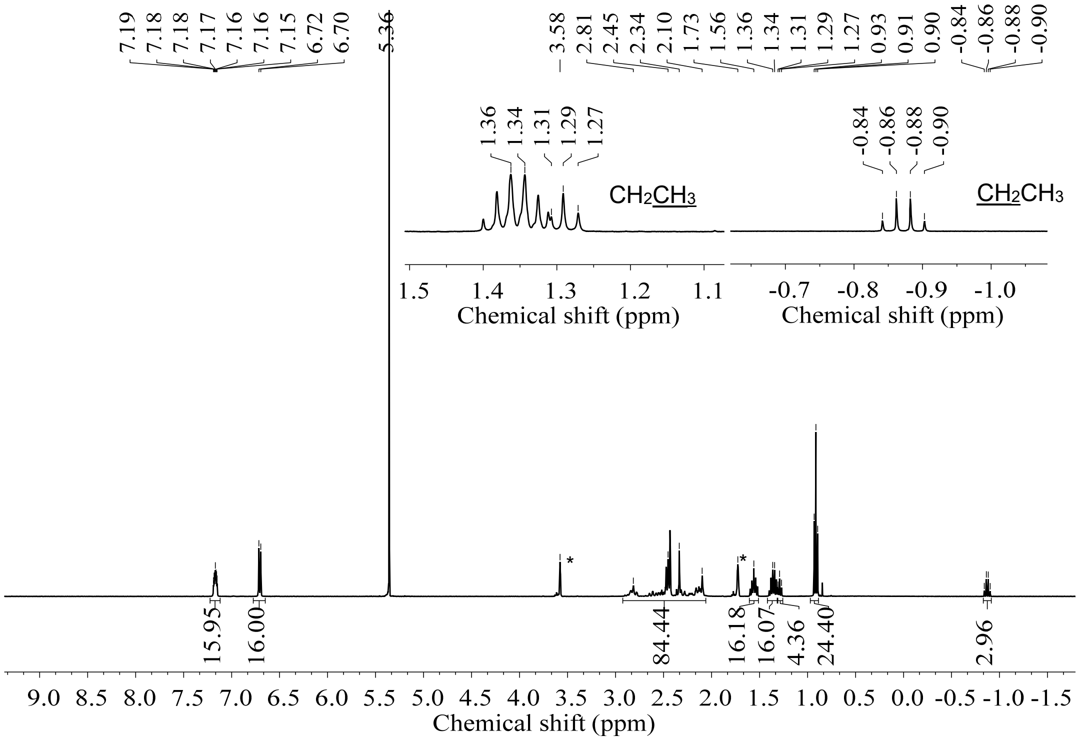


Figure S42: *In-situ* $^1$H NMR spectrum of the reaction of **3b** with ethylene (1 bar) in THF-d8 (*) at 25 °C.

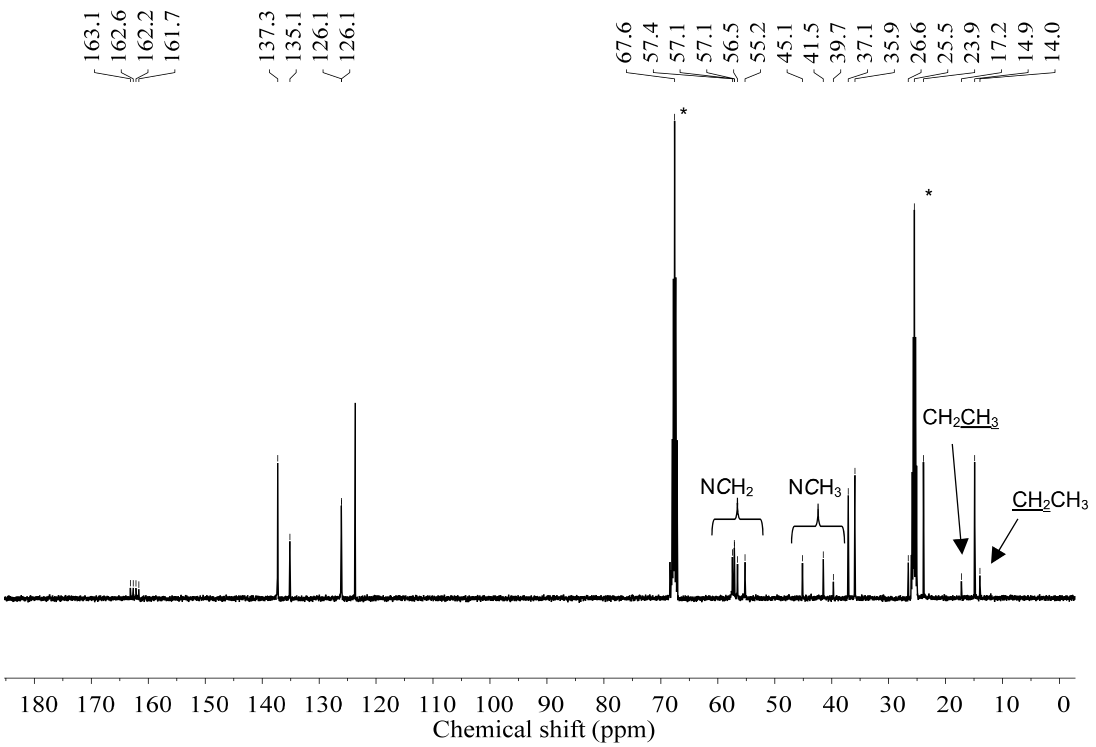


Figure S43: *In-situ* $^{13}C$ NMR spectrum of the reaction of **3b** with ethylene (1 bar) in THF-d8 (*) at −20 °C.

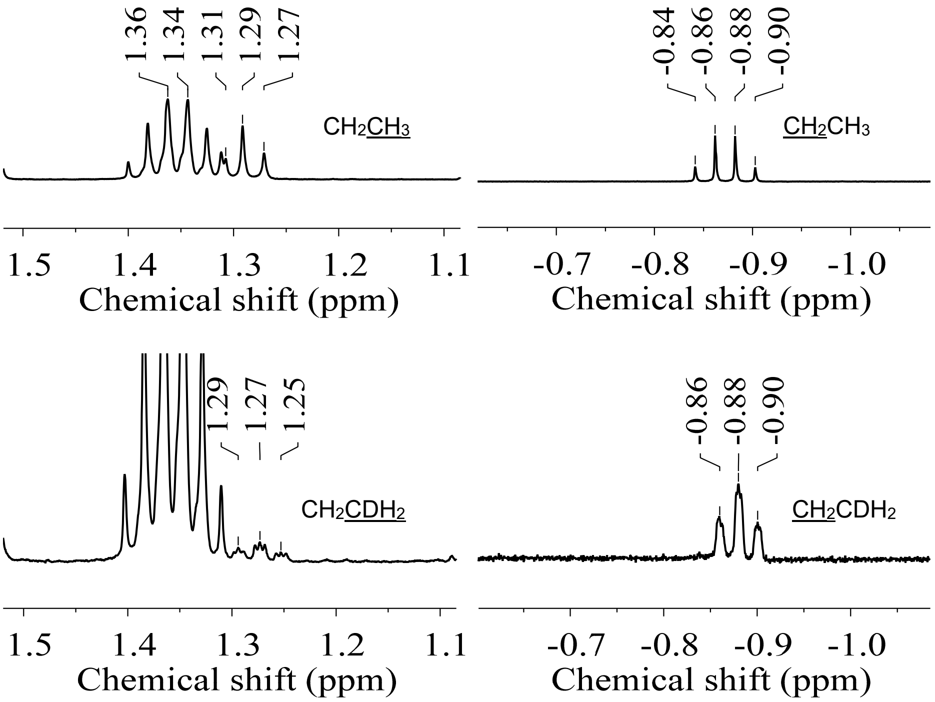


Figure S44: Cut-out of the *in-situ* $^{1}H$ NMR spectra of the reaction of **3b** (top) and **3b-*d*2** (bottom) with ethylene (1 bar) in THF-$d_8$ at 25 °C.

3.1.3 Reaction with 1-Octene

In a J. Young-type NMR tube, $[(Me_5PACP)_2Ca_2(\mu\text{-}H)_2][B(C_6H_4\text{-}4\text{-}^nBu)_4]_2$ (10 mg, 6 µmol) or $[(Me_5PACP)_2Ca_2(\mu\text{-}D)_2][B(C_6H_4\text{-}4\text{-}^nBu)_4]_2$ (10 mg, 6 µmol) was dissolved in 0.4 mL of THF-$d_8$. 1-octene (2 mg, 18 µmol) and 1,4-$(SiMe_3)_2C_6H_4$ (3 mg, 13 µmol) in 0.1 mL of THF-$d_8$ were added and the mixture analyzed by NMR spectroscopy.

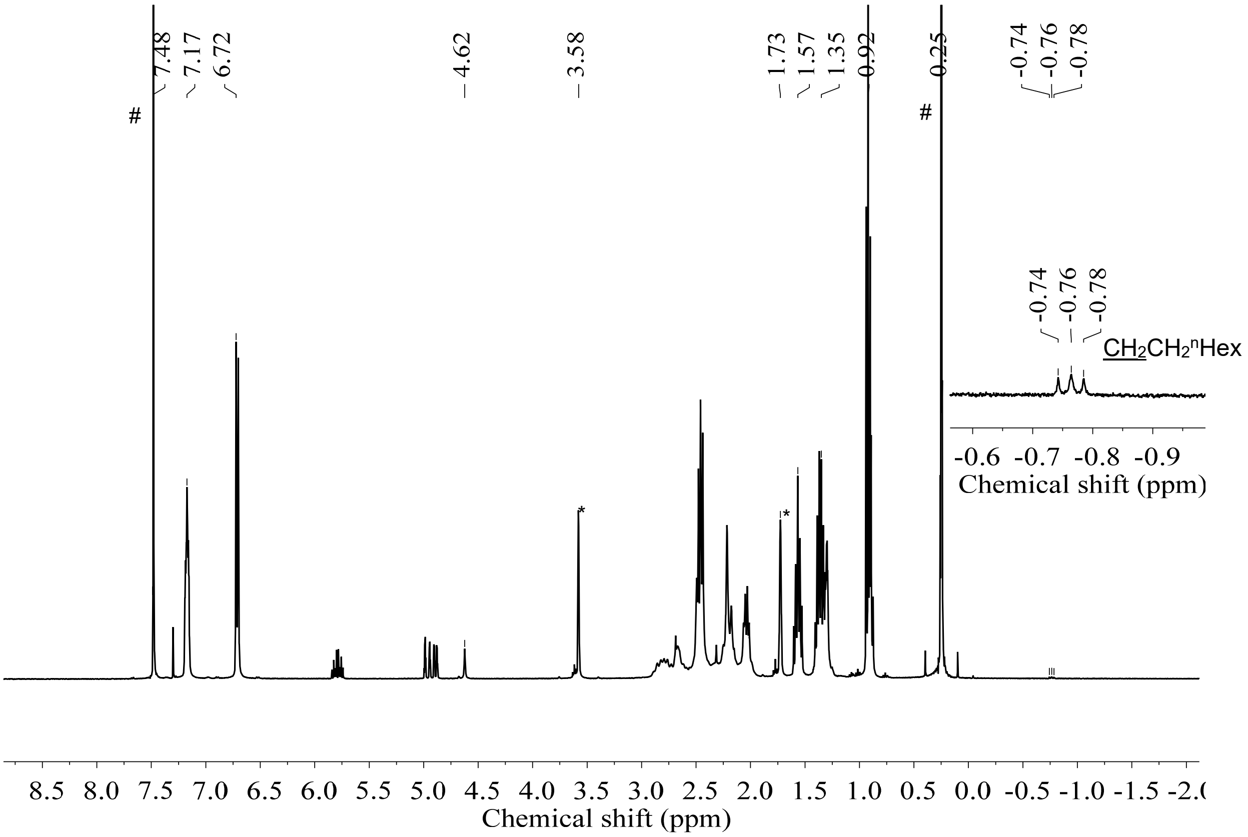


Figure S45: *In-situ* $^1H$ NMR of the reaction of **3b** with 1-octene in THF-$d_8$ (*) at 25 °C. # denotes 1,4-$(SiMe_3)_2C_6H_4$.

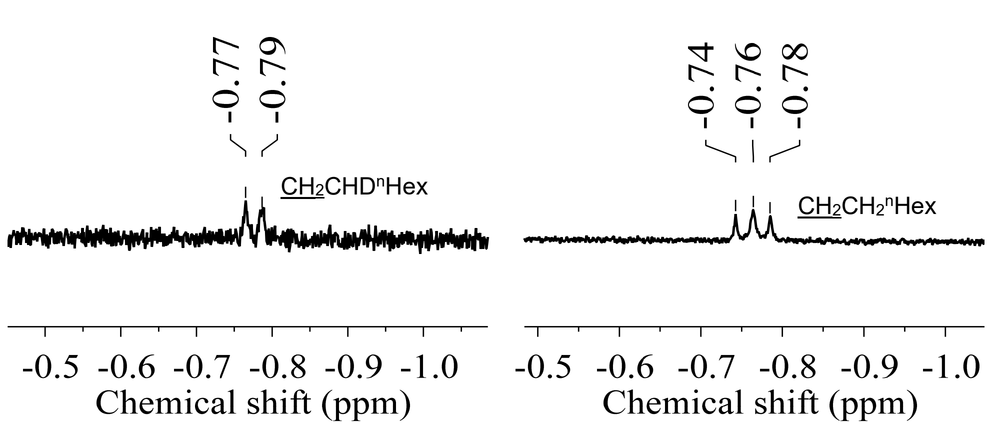


Figure S46: Cutout of the *in-situ* $^1H$ NMR spectra of the reaction of **3b-$d_2$** (left) and **3b** (right) with 1-octene in THF-$d_8$ at 25 °C.

## 3.2 Hydrogenation and Hydrosilylation Catalysis

For catalytic experiments, three stock solutions were prepared. One stock solution contained $[(Me_5PACP)_2Ca_2(\mu\text{-}H)_2][B(C_6H_4\text{-}4\text{-}^nBu)_4]_2$ (42 mg, 24 µmol in 0.8 mL THF-$d_8$), a second stock solution contained (n-octy)methylsilane (143 mg, 0.9 mmol in 1.5 mL THF-$d_8$) and a third stock solution contained 1-octene (101 mg, 0.9 mmol in 1.5 mL THF-$d_8$) and the internal standard 1,4-$(SiMe_3)C_6H_4$ (50 mg, 225 µmol).

For hydrogenation experiments, 0.1 mL of the catalyst stock solution and the olefin stock solution were transferred into a J. Young-style NMR tube and 0.4 mL pure THF-$d_8$ was added. The reaction mixture was degassed applying three "freeze-pump-thaw" cycles and charged with $H_2$ (1 bar). Progress of the reaction was monitored by $^1$H NMR spectroscopy.

For hydrosilylation experiments, 0.1 mL of the catalyst stock solution, the olefin stock solution and the silane stock solution were transferred into a J. Young-style NMR tube and 0.3 mL pure THF-$d_8$ was added. The reaction progression was monitored by $^1$H NMR spectroscopy.

For the hydrosilylation of ethylene, $[(Me_5PACP)_2Ca_2(\mu\text{-}H)_2][B(C_6H_4\text{-}4\text{-}^nBu)_4]_2$ (4.4 mg, 2.5 µmol) and n-OctMeSiH$_2$ (16 mg, 0.1 mmol) were combined in THF-$d_8$ (0.6 mL), the solution degassed applying three "freeze-pump-thaw" cycles and pressurized with ethylene (1 bar). The reaction progression was monitored by $^1$H NMR spectroscopy.

## 3.3 Kinetic Investigations

Stock solutions for hydrosilylation experiments with catalyst **3b** were prepared as described in 3.2. The hydride stock solution for hydrosilylation experiments with catalyst **6** was prepared by dissolving [($Me_4$TACD)H][B($C_6H_4$-4-$^n$Bu)$_4$] (52 mg, 30 μmol) in THF-$d_8$ (1 mL).

For each catalytic experiment, the corresponding amount of the three stock solutions was added to a J.Young-style NMR tube. For experiments with a ten-fold excess of olefin or silane, these substrates were weighed out and added separately. In all experiments, THF-$d_8$ was added until the total reaction volume reached 0.6 mL and progress of the reaction was monitored by $^1$H NMR spectroscopy.

The spectroscopic data was processed using MestReNova Version 12.0.1. Partial reaction orders where examined applying Burés' method (Variable Time Normalization Analysis) using Microsoft Excel software.[S6]

### 3.3.1 Reaction Progress

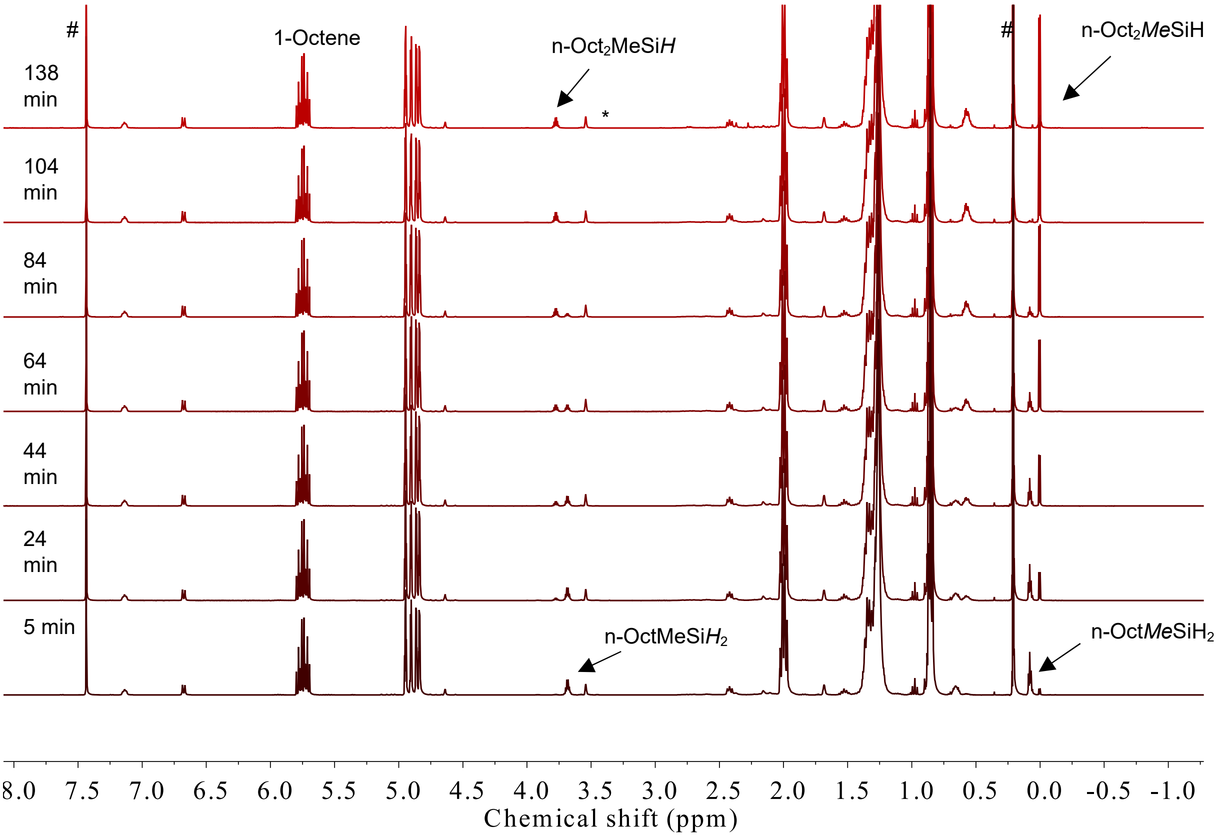


Figure S47: Exemplatory stacked $^1$H NMR spectra showing the reaction progress for the hydrosilylation of 1-octene mediated by **3b** in THF-$d_8$ (*) at 25 °C. Spectra are shown for the reaction with a tenfold excess of 1-octene.

## 3.3.2 Methyl group shuffling

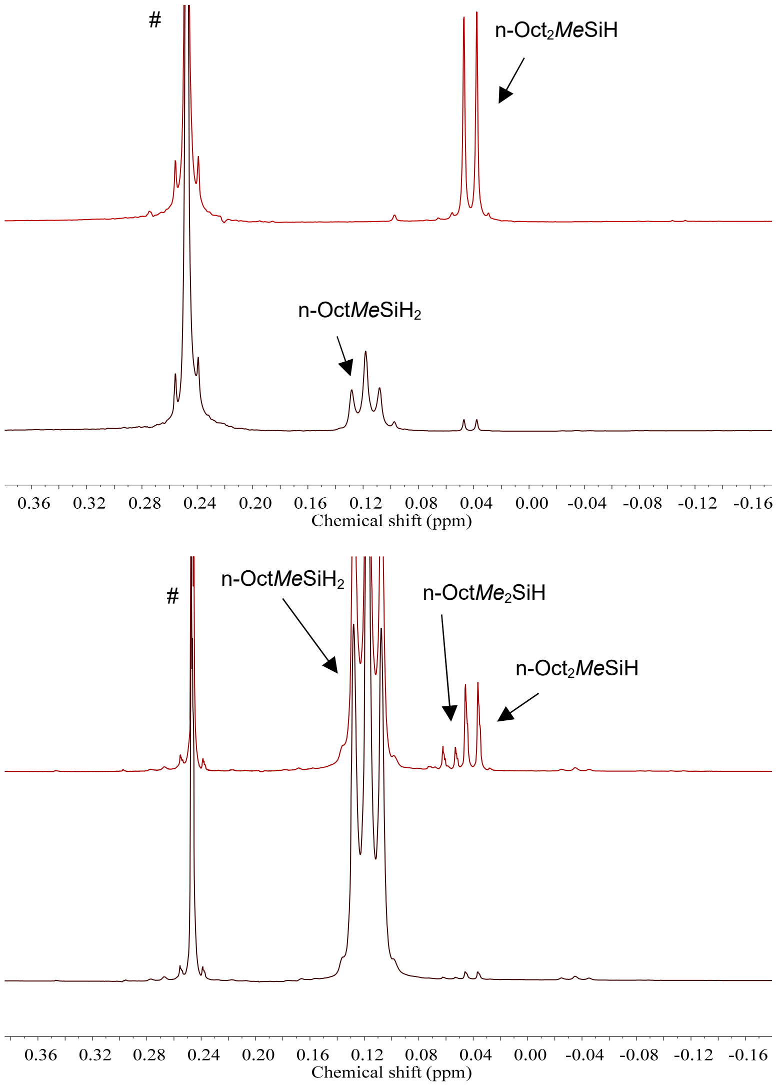


Figure S48: Comparison between the hydrosilylation with an excess of 1-octene (top) after 5 minutes and after 138 minutes and an excess of n-octylmethylsilane (bottom) after 14 minutes and after 24 hours. #denotes internal standard 1,4-$(SiMe_3)_2C_6H_4$. The methyl groups of substrate, product and side-product are highlighted.

### 3.3.3 Kinetic Isotope Effect

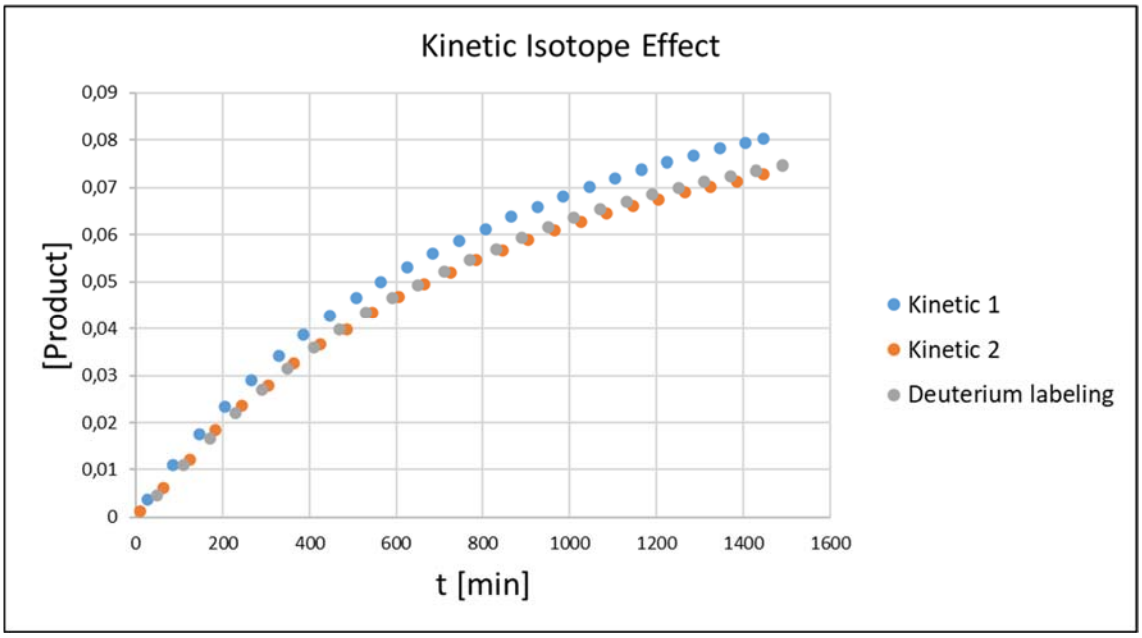


Figure S49: Concentration profiles of the hydrosilylation of 1-octene with n-OctMe$SiH_2$/n-OctMe$SiD_2$. In all cases, substrate concentrations were 0.1 M with 5 mol% of **3b** used as the catalyst. Kinetic 1 and Kinetic 2 were performed with n-OctMe$SiH_2$ under identical conditions and display the expected margin of error for the concentration profiles.

### 3.3.4 Variable Time Normalization Analysis for $[(Me_5PACP)_2Ca_2(\mu\text{-}H)_2]^{2+}$ mediated hydrosilylation

#### 3.3.4.1 Partial reaction order of the catalyst

Table S1: Variation of catalyst concentration for determination of the partial reaction order of **3b**.

| [**3b**] (mol·L$^{-1}$) | [1-octene] (mol·L$^{-1}$) | [$^{n}$OctMeSiH$_2$] (mol·L$^{-1}$) |
|---|---|---|
| 0.005 | 0.1 | 0.1 |
| 0.0075 | 0.1 | 0.1 |
| 0.01 | 0.1 | 0.1 |

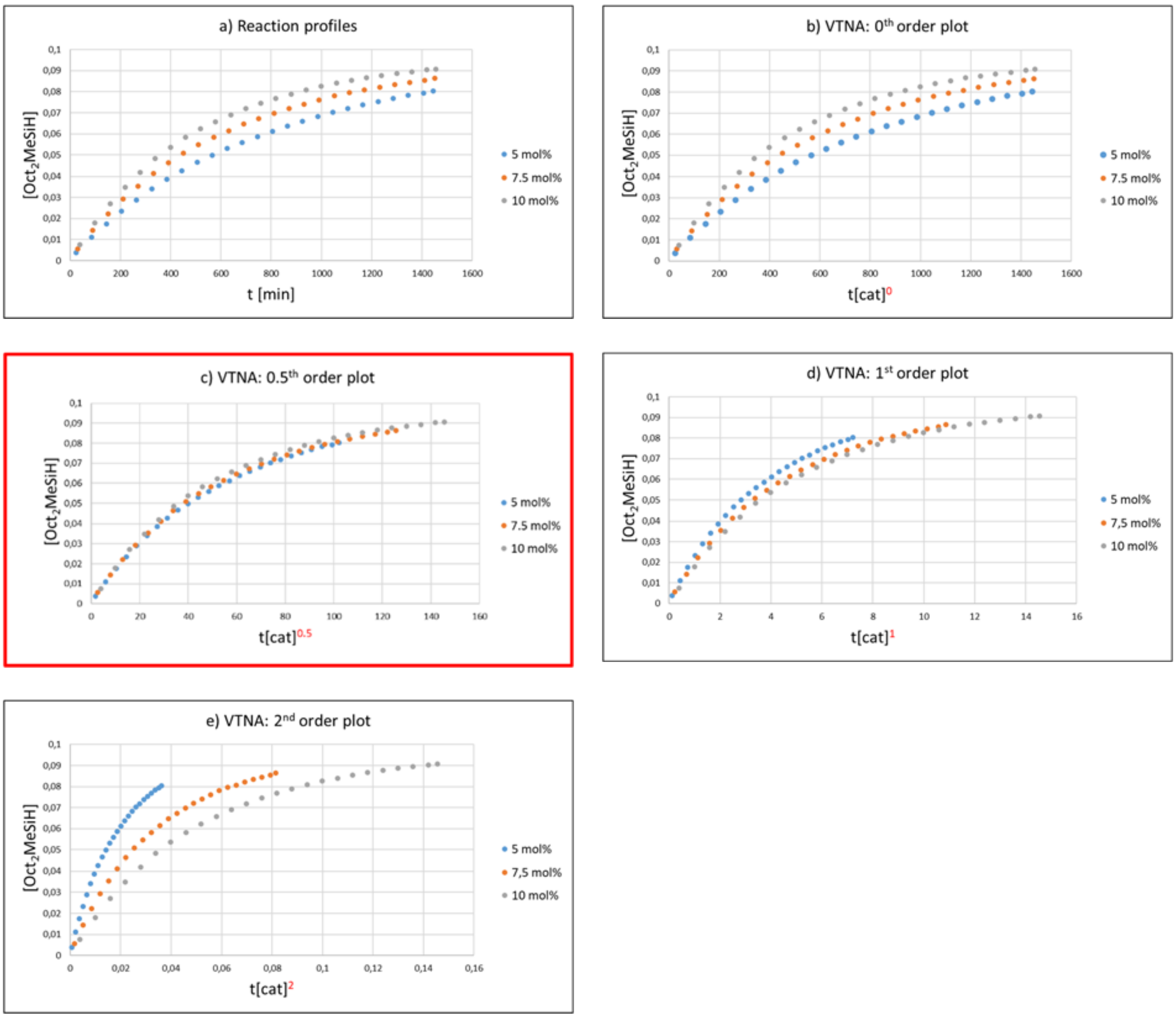


Figure S50: Effect of catalyst concentration on hydrosilylation catalysis mediated by **3b**. a) Reaction profile for different catalyst concentrations. b) to e) VTNA plots for different partial reaction orders of **3b**. c) shows that a 0.5$^{th}$ partial reaction order for **3b** gives a fitting plot.

### 3.3.4.2 Partial reaction order of the alkene

Table S2: Variation of 1-octene concentration for determination of the partial reaction order of the alkene.

| [**3b**] (mol·L$^{-1}$) | [1-octene] (mol·L$^{-1}$) | [$^{n}$OctMeSiH$_2$] (mol·L$^{-1}$) |
|---|---|---|
| 0.005 | 0.1 | 0.1 |
| 0.005 | 0.125 | 0.1 |
| 0.005 | 0.15 | 0.1 |
| 0.005 | 0.2 | 0.1 |
| 0.005 | 1 | 0.1 |

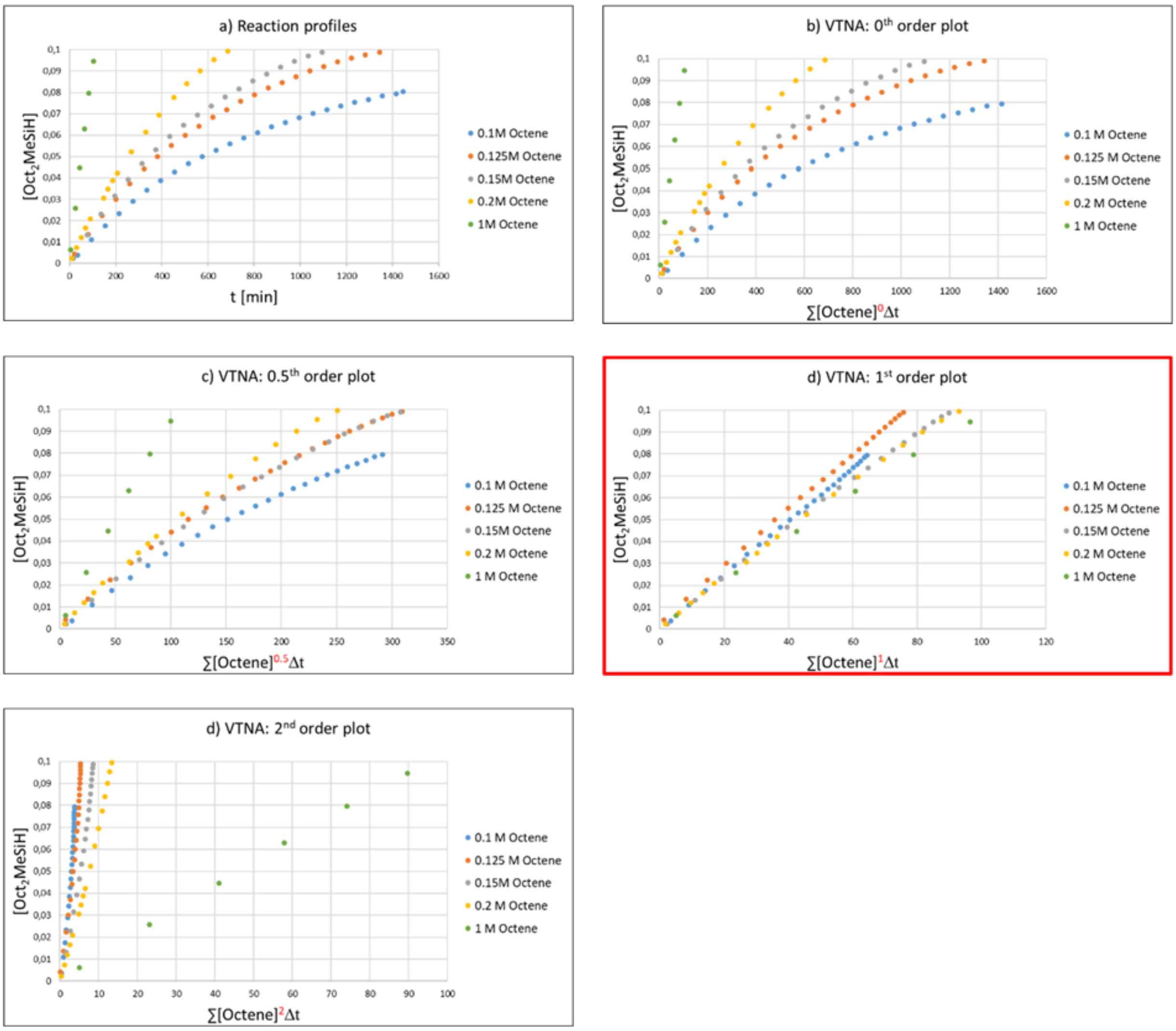


Figure S51: Effect of catalyst concentration on hydrosilylation catalysis mediated by **3b**. a) Reaction profile for different 1-octene concentrations. b) to e) VTNA plots for different partial reaction orders of 1-octene. d) shows that a 1$^{st}$ partial reaction order for the alkene gives a fitting plot.

.

### 3.3.4.3 Partial reaction order of the silane

Table S3: Variation of n-octylmethylsilane concentration for determination of the partial reaction order of the silane.

| [**3b**] (mol·L$^{-1}$) | [1-octene] (mol·L$^{-1}$) | [$^{n}$OctMeSiH$_2$] (mol·L$^{-1}$) |
|---|---|---|
| 0.005 | 0.1 | 0.1 |
| 0.005 | 0.1 | 0.125 |
| 0.005 | 0.1 | 0.15 |
| 0.005 | 0.1 | 0.2 |
| 0.005 | 0.1 | 1 |

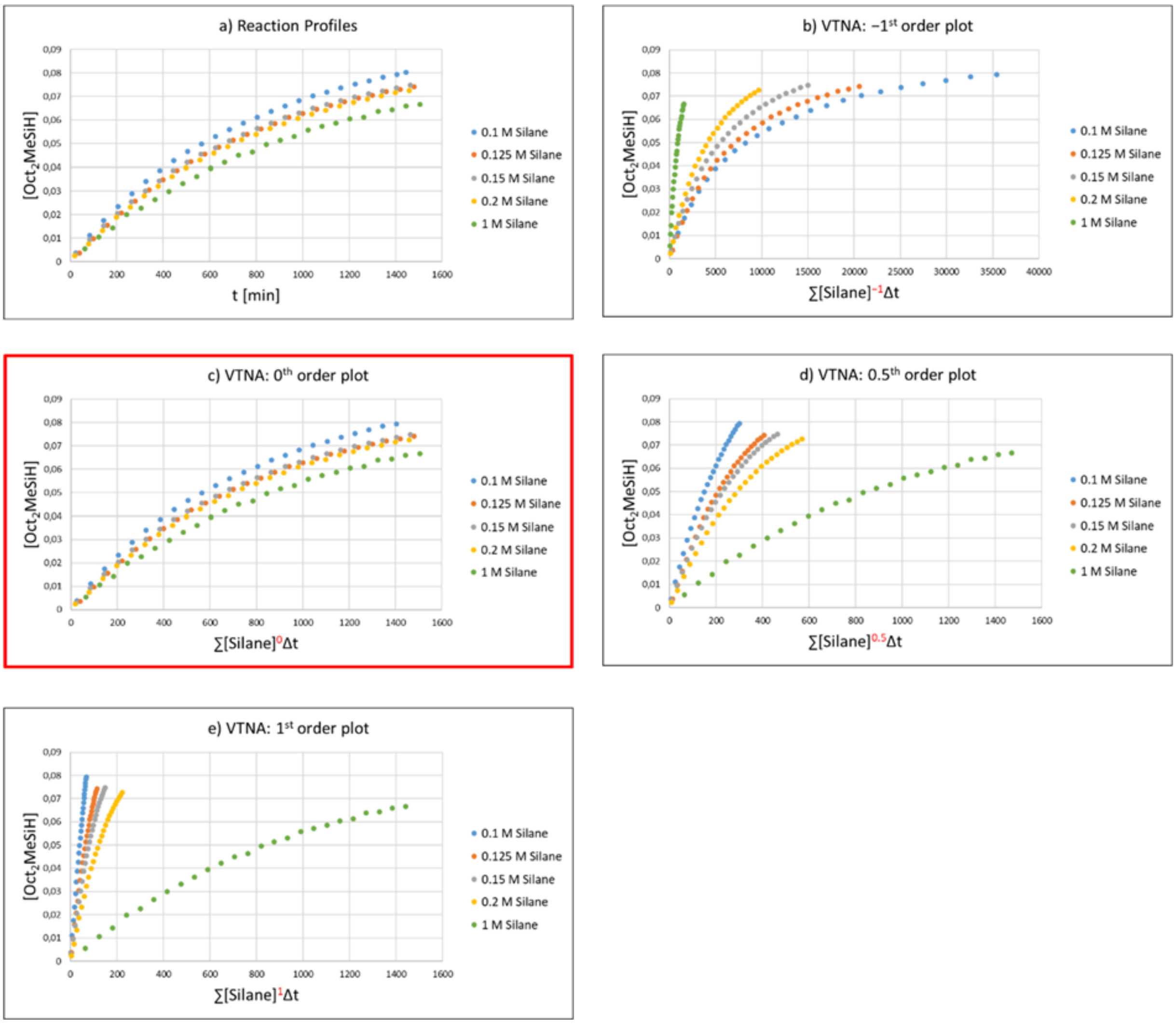


Figure S52: Effect of silane concentration on hydrosilylation catalysis mediated by **3b**. a) Reaction profile for different silane concentrations. b) to e) VTNA plots for different partial reaction orders of n-octylmethylsilane. c) shows that a 0$^{th}$ partial reaction order for the silane gives a fitting plot.

### 3.3.5 Variable Time Normalization Analysis for $[(Me_4TACD)_2Ca_2(\mu\text{-}H)_2(thf)]^{2+}$ mediated hydrosilylation

#### 3.3.5.1 Partial reaction order of the catalyst

Table S4: Variation of catalyst concentration for determination of the partial reaction order of **6**.

| [**6**] (mol·L$^{-1}$) | [1-Octene] (mol·L$^{-1}$) | [$^{n}OctMeSiH_2$] (mol·L$^{-1}$) |
|---|---|---|
| 0.005 | 0.1 | 0.1 |
| 0.0075 | 0.1 | 0.1 |
| 0.01 | 0.1 | 0.1 |

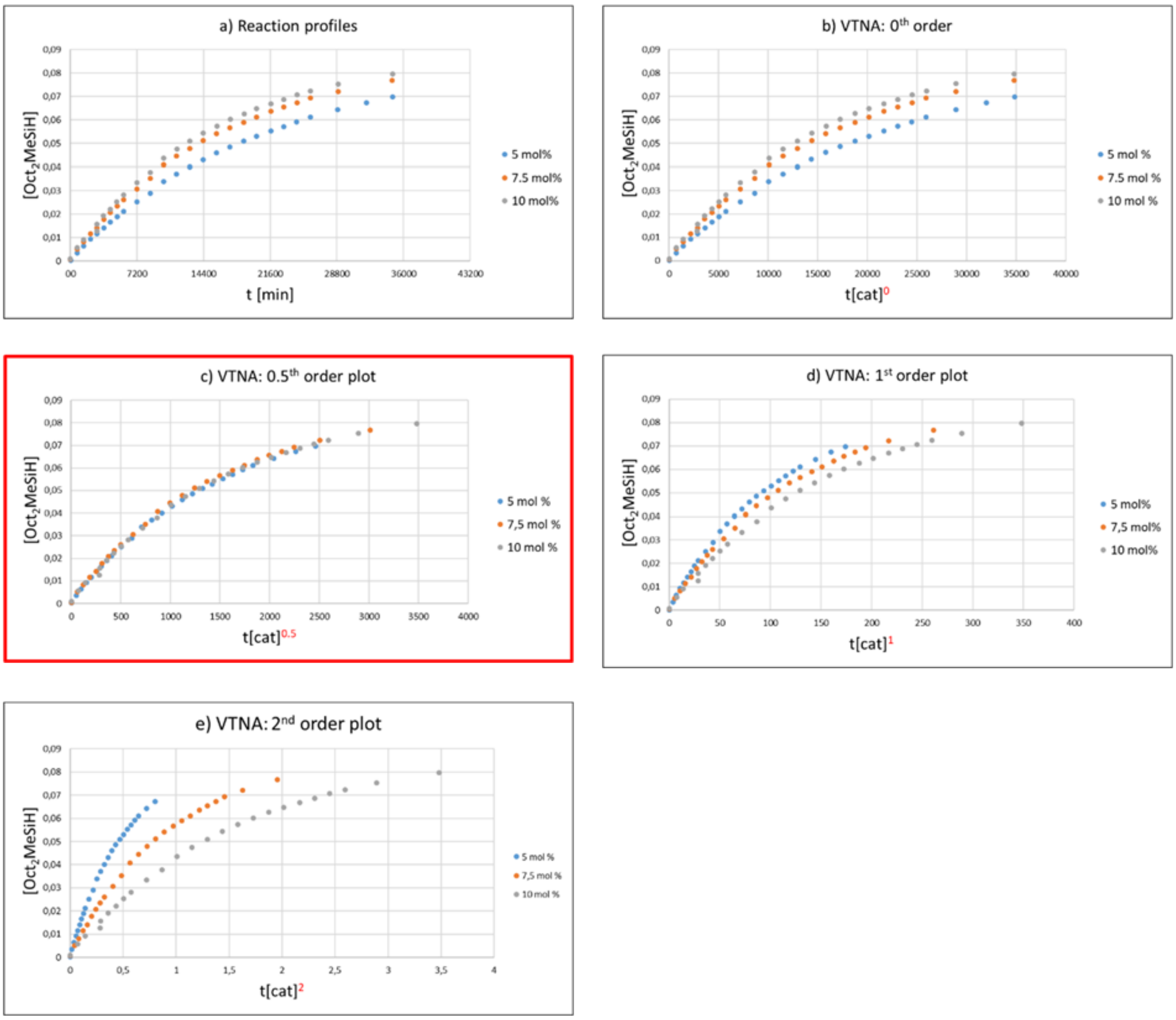


Figure S53: Effect of catalyst concentration on hydrosilylation catalysis mediated by **6**. a) Reaction profile for different catalyst concentrations. b) to e) VTNA plots for different partial reaction orders of **6**. c) shows that a 0.5$^{th}$ partial reaction order for **6** gives a fitting plot.

### 3.3.5.2 Partial reaction order of the alkene

Table S5: Variation of 1-octene concentration for determination of the partial reaction order of the alkene.

| [**6**] (mol·L$^{-1}$) | [1-Octene] (mol·L$^{-1}$) | [$^{n}$OctMeSiH$_2$] (mol·L$^{-1}$) |
|---|---|---|
| 0.005 | 0.1 | 0.1 |
| 0.005 | 0.2 | 0.1 |
| 0.005 | 1 | 0.1 |

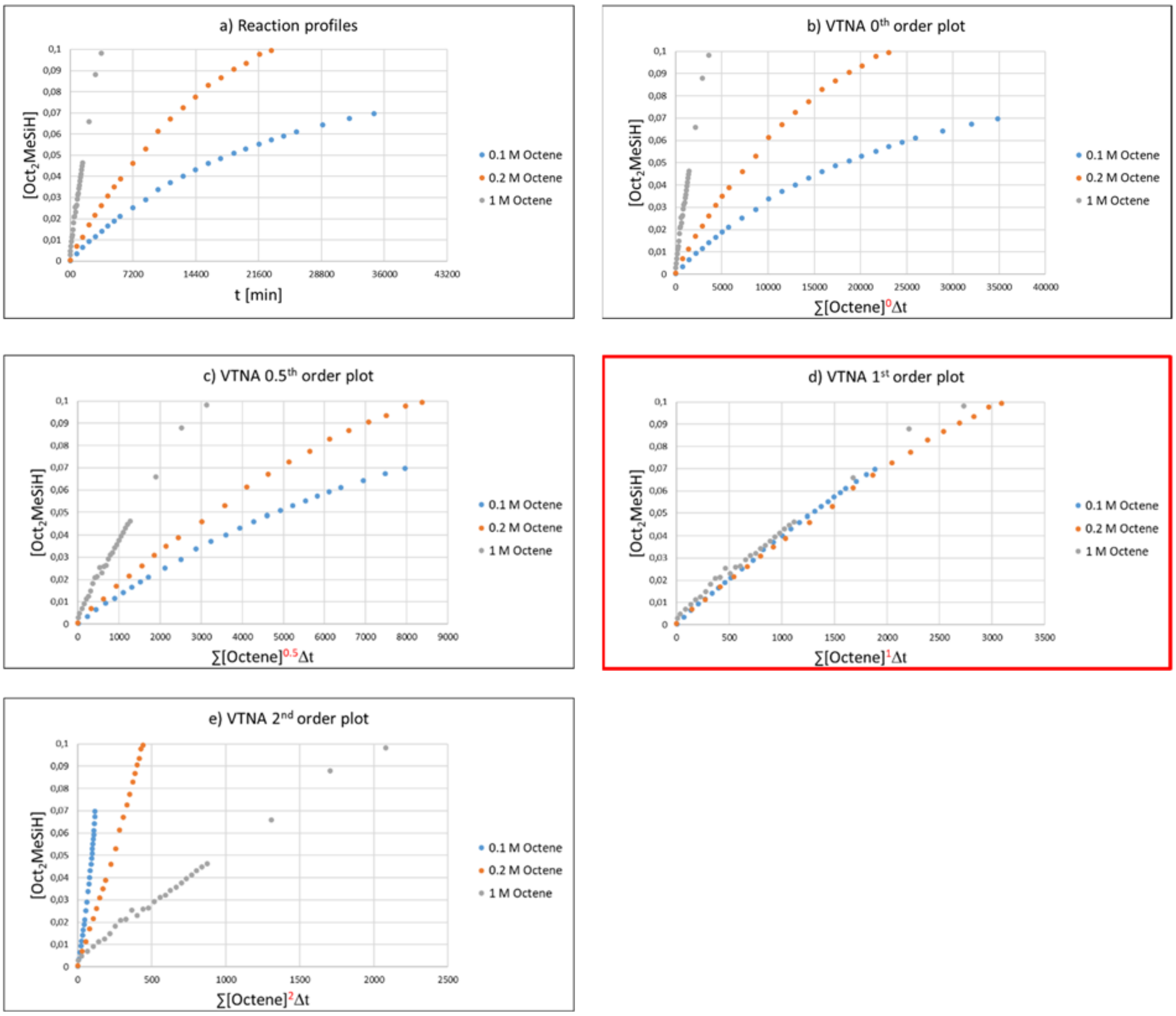


Figure S54: Effect of catalyst concentration on hydrosilylation catalysis mediated by **6**. a) Reaction profile for different 1-octene concentrations. b) to e) VTNA plots for different partial reaction orders of 1-octene. d) shows that a 1$^{st}$ partial reaction order for the alkene gives a fitting plot.

### 3.3.5.3 Partial reaction order of the silane

Table S6: Variation of n-octylmethylsilane concentration for determination of the partial reaction order of the silane.

| [**6**] (mol·L$^{-1}$) | [1-octene] (mol·L$^{-1}$) | [$^{n}$OctMeSiH$_2$] (mol·L$^{-1}$) |
|---|---|---|
| 0.005 | 0.1 | 0.1 |
| 0.005 | 0.1 | 0.2 |
| 0.005 | 0.1 | 1 |

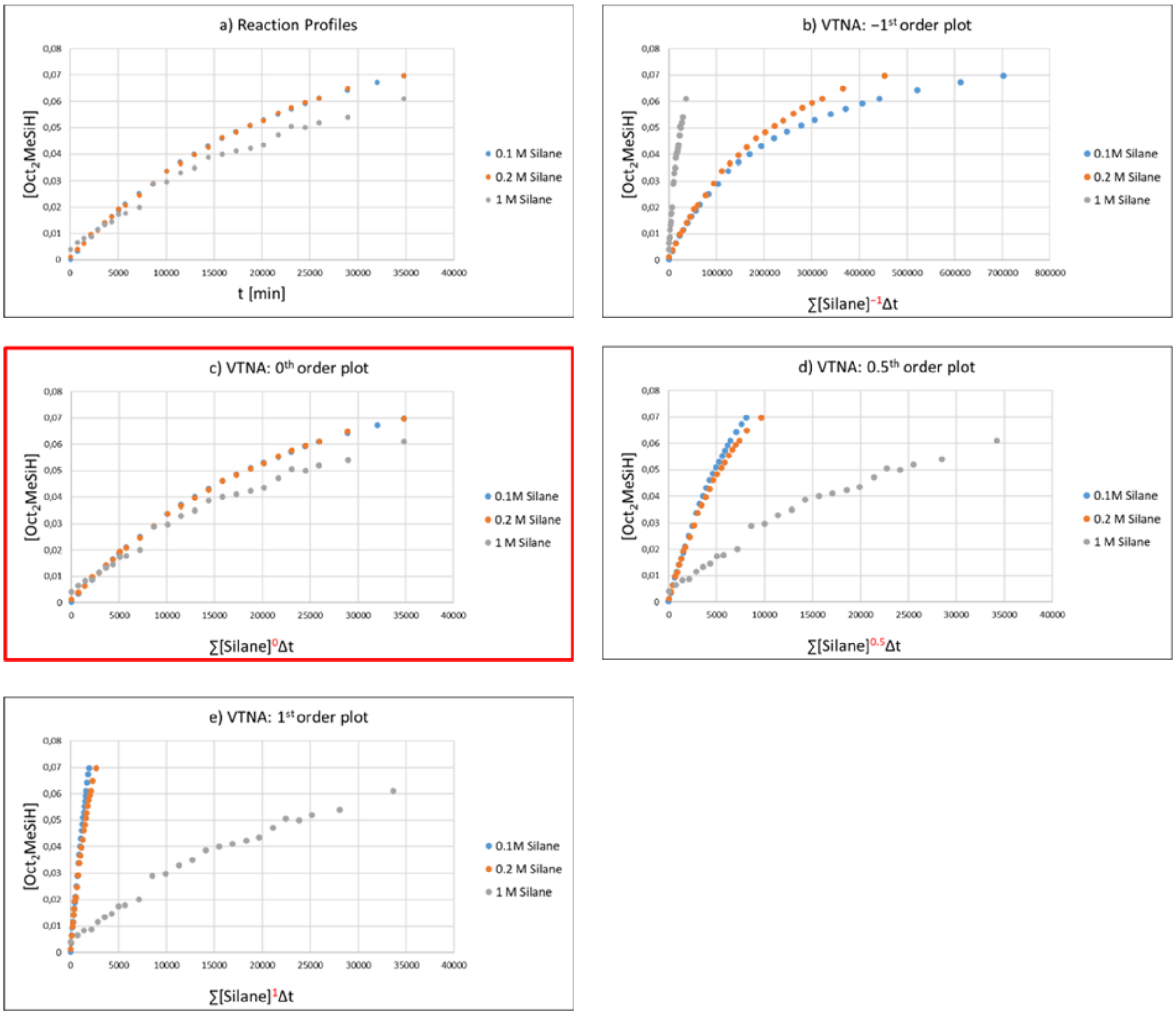


Figure S55: Effect of silane concentration on hydrosilylation catalysis mediated by **6**. a) Reaction profile for different silane concentrations. b) to e) VTNA plots for different partial reaction orders of n-octylmethylsilane. c) shows that a 0$^{th}$ partial reaction order for the silane gives a fitting plot.

3.3.6 Determination of $k_{obs}$.

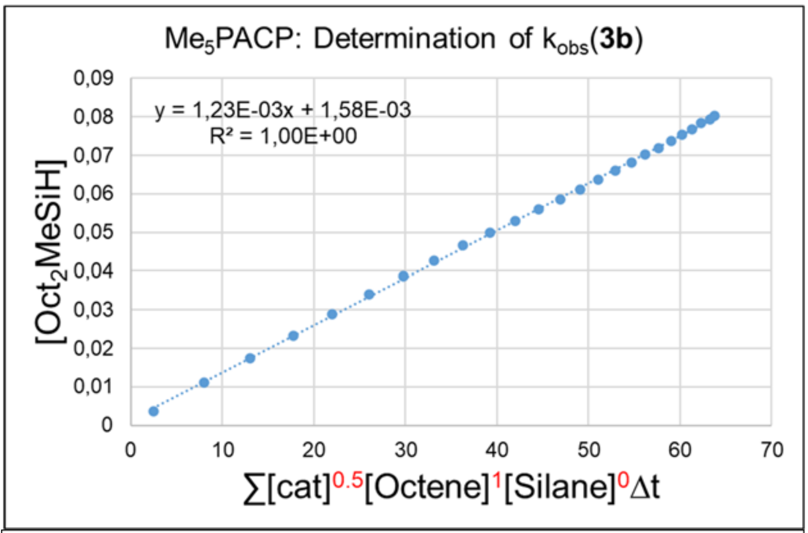


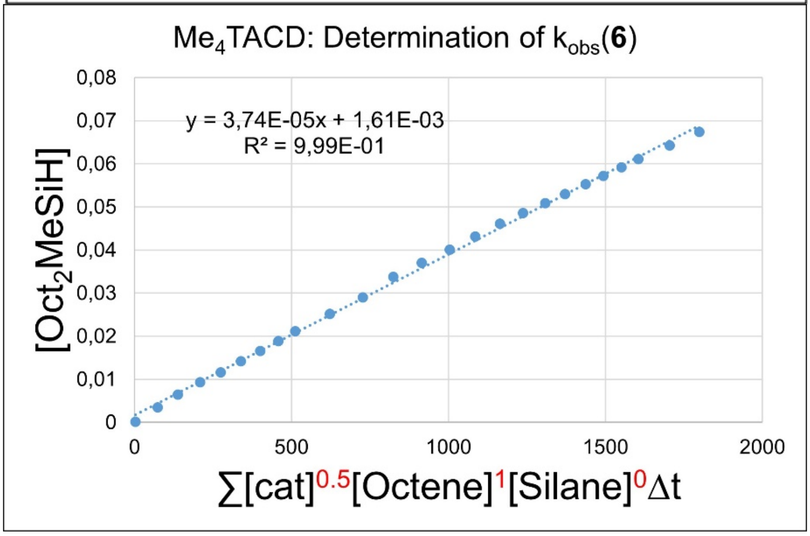


Figure S56: Determination of $k_{obs}$ for **3b** (top) and **6** (bottom). The slope of the linear regression line corresponds to $k_{obs}$ of the reaction. The kinetic data is taken from the reactions carried out with 5 mol% of catalyst and 0.1 M of substrate in 0.6 mL of THF-$d_8$.

# 4 Crystal Structure Determinations

X-ray diffraction data were collected at 100 K on an Eulerian 4-circle diffractometer STOE STADIVARI in ω-scan mode with Mo-Kα (**1b**) or with Cu-Kα (**3b**, **6**). The crystal structures were solved by direct methods using SHELXT[S7] and all refinements were carried out against $F^2$ with SHELXL[S8] as implemented in the program system Olex2.[S9] The molecular cation in **3b** shows crystallographic $\bar{1}$ symmetry around Wyckoff position 1*g*. The crystal packing of **3b** contains crystallographically independent, non-coordinated molecules of THF. The lattice of **6** also contains non coordinated THF molecules of THF. Because these could not be reliably refined, they were not included in the structure factor calculation with their atom positions and the SQUEEZE routine was applied using the program “solvent mask” as implemented in Olex2. In **3b**, disorder was found for the atoms C5 and C6 of the ligand $Me_5$PACP, for a methyl group of the borate (C35), and for the atoms C56 as well as C61, C62, C63 within non-coordinated THF. This disorder could be resolved well with split positions. Due to disorder in a THF group, one distance restraint (concerning the atoms C61B and C62B) was applied using the command DFIX as implied in SHELXL. In **6**, distance restraints were included in the refinement of the disordered carbon atoms of a $Me_4$TACD ligand (C1-C8) as well as for the bond C37 between C38 (the latter being refined with split positions).

Non-hydrogen atoms were refined with anisotropic displacement parameters. Restraints were employed using the command RIGU as implied in the program SHELXL for the refinement of the anisotropic displacement parameters of atoms C28 and C29 in **3b**. Hydrogen atoms were included in idealized positions. Only the hydrogen atom of the N*H* unit in **1b** (H1), the hydride atom in **3b** (H1), as well the hydride atoms in **6** (H1 and H2) were located in a Fourier difference map and were refined with isotropic displacement parameters. Refinement results are given in Table S7. Graphical representations were performed with the program DIAMOND.[S10]

Crystals of **4** were obtained at -40 °C from THF/n-pentane under an atmosphere of ethylene within eight weeks. X-ray diffraction data were collected with a crystal of dimensions 0.32 × 0.33 × 0.35 mm at 100 K on an Eulerian 4-circle diffractometer STOE STADIVARI in ω-scan mode with Cu-Kα radiation. Not all reflections could be assigned with hkl values. Indexing 75711 reflections gave a unit cell of $a$ = 16.071(3) Å, $b$ = 19.404(4) Å, $c$ = 20.219(4) Å, β = 96.72(3)°, $V$ = 6262(2) Å$^3$. Refinement in space group $P2_1/c$ (no. 14) based on 12100 reflections (6471 with $I > 2\sigma(I)$, $R_{int.}$ = 0.0375, $I/\sigma(I)$ = 0.0389, 3.2 ≤ $\theta$ ≤ 72.0, -17 ≤ $h$ ≤ 19, -16 ≤ $k$ ≤ 23, -24 ≤ $l$ ≤ 20) gave a structure model corresponding to a composition of $C_{21}H_{48}CaN_5O$, $C_{40}H_{50}B$, 0.5($C_4H_8O$) for Z = 4 and showed the expected connectivity with a terminal ethyl group coordinated to a calcium center. Refinement values of $R1$ = 0.1531, $wR2$ = 0.4211 (based on 6471 reflections with $I > 2\sigma(I)$) and with $R1$ = 0.2038, $wR2$ = 0.4522 (based on all 12100

reflections) were obtained with a goodness of fit of 1.551. We note that the displacement parameters in the structure model of **4b** are comparatively high for a data collection at 100 K. Re-integration of the same dataset allowed indexing of 315670 reflections, but still not all reflections could be assigned with hkl values. The structure could be solved with new cell parameters of $a$ = 41.7851(17) Å, $b$ = 19.3953(7) Å, $c$ = 39.9392(17) Å, $\beta$ = 104.457(3)°, $V$ = 31343(2) Å$^3$ in space group $P2_1/c$ with five crystallographically independent molecules ($Z$ = 4). As with the smaller unit cell, the solution reveals a monomeric calcium center with a terminal ethyl group, but the refinement based on 59728 reflections. (20391 with $I > 2\sigma(I)$, $R_{int.}$ = 0.0972, $I/\sigma(I)$ = 0.0645, 2.3 ≤ $\theta$ ≤ 72.2, -51 ≤ $h$ ≤ 44, -23 ≤ $k$ ≤ 16, -37 ≤ $l$ ≤ 49) still showed severe disorder of the *n*-butyl groups and was not satisfactory. We assume that our structure model represents a simplified average and that a more severe crystallographic problem, e.g. a modulation, still remains unsolved. This agrees with the high displacement parameters.

CCDC-2034969 (**1b**), CCDC-2034970 (**3b**), CCDC-2034971 (**6**), CCDC-2043816 (**4b**) contain the supplementary crystallographic data for this paper. These data can be obtained free of charge from the Crystallographic Data Centre via www.ccdc.cam.ac.uk/data_request/cif.

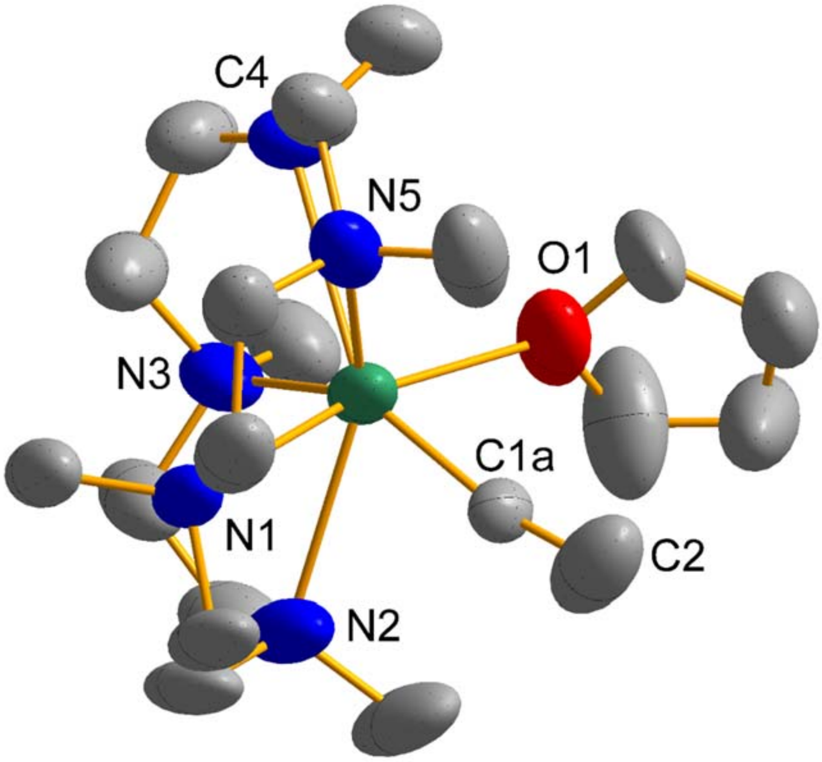


Figure S57: DIAMOND representation of the average structure of the cationic part of **4b**. Hydrogen atoms are omitted. Atom C1 which was refined with split positions is shown with one positional isomer.

Table S7: Crystallographic data of **1b**, **3b** and **5**.

| | **1b** | **3b** | **6** |
|---|---|---|---|
| formula | $C_{15}H_{36}N_5$, $C_{40}H_{52}B$ | $C_{30}H_{72}Ca_2N_{10}$, 2($C_{40}H_{52}B$), 4($C_4H_8O$) | $C_{28}H_{66}Ca_2N_8O$, 2($C_{40}H_{52}B$) |
| *Fw* / g·mol$^{-1}$ | 830.11 | 2028.80 | 1698.29 |
| cryst. color, habit | colorless block | colorless plate | colorless plate |
| crystal size / mm | 0.32 × 0.33 × 0.35 | 0.09 × 0.19 × 0.36 | 0.07 × 0.19 × 0.27 |
| crystal system | monoclinic | triclinic | triclinic |
| space group | $P2_1/n$ | $P\bar{1}$ | $P\bar{1}$ |
| *a* / Å | 13.5386(8) | 14.1093(16) | 14.7282(4) |
| *b* / Å | 19.0407(12) | 15.3694(18) | 17.6655(5) |
| *c* / Å | 19.9140(13) | 16.5715(18) | 25.1789(7) |
| *α* / ° | | 97.386(9) | 98.216(2) |
| β / ° | 94.143(5) | 102.439(9) | 93.584(2) |
| *γ* / ° | | 117.225(8) | 107.277(2) |
| *V* / Å$^3$ | 5120.1(6) | 3011.8(6) | 6153.0(3) |
| *Z* | 4 | 1 | 2 |
| $d_{calc}$ / Mg·m$^{-3}$ | 1.077 | 1.119 | 0.917 |
| μ(MoKα) / mm$^{-1}$ | 0.062 | | |
| μ(CuKα) / mm$^{-1}$ | | 1.232 | 1.111 |
| *F*(000) | 1832 | 1116 | 1864 |
| *θ* range / ° | 2.05, 26.38 | 3.35, 72.56 | 3.93, 71.81 |
| index ranges | -14 ≤ *h* ≤ 16, -23 ≤ *k* ≤ 23, -24 ≤ *l* ≤ 24 | -17 ≤ *h* ≤ 13, -17 ≤ *k* ≤ 18, -19 ≤ *l* ≤ 20 | -17 ≤ *h* ≤ 18, -19 ≤ *k* ≤ 21, -16 ≤ *l* ≤ 30 |
| refln. | 38231 | 30221 | 43771 |
| independ. reflns ($R_{int}$) | 10453 (0.0597) | 11178 (0.0365) | 21643 (0.0455) |
| observed reflns | 5900 | 7834 | 11802 |
| data/ restr./ param. | 10453 / 0 / 563 | 11178 / 4 / 730 | 43771 / 30 / 1090 |
| $R_1$, $wR2$ [$I > 2\sigma(I)$] | 0.0642, 0.1677 | 0.0644, 0.1801 | 0.0741, 0.2058 |
| $R_1$, $wR2$ (all data) | 0.1156, 0.1888 | 0.0851, 0.1901 | 0.1142, 0.2232 |
| GooF on $F^2$ | 0.972 | 1.085 | 0.942 |
| largest diff. peak, hole / e·Å$^3$ | 1,133, -0.353 | 0.977, -0.563 | 0.693, -0.510 |
| CCDC number | 2034969 | 2034970 | 2034971 |

# 5 Computational Details

Calculations were carried out at the DFT level using the hybrid functional B3PW91[S11] with the Gaussian 09[S12] suite of programs. Polarized all-electron triple-ζ 6-311G(d,p)[S13] basis sets were used for Ca, C, H, O and N. Geometry optimization was carried out without any symmetry restriction. The nature of the extrema (minimum or transition state) was verified with analytical frequency calculations. The NBO analysis[S14] was finally carried out on the optimized geometry.

Ca NBO:

| | NBO | Bond length | Occupancy | Center(bond % contribution) | Hybridization (function,%) | Wiberg bond index matrix |
|---|---|---|---|---|---|---|
| 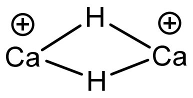  | Ca7-H17 | 2.31324 | 1.78077 | Ca(10.98%);H(89.02%) | Ca(s21 p63 d16);H(s100) | 0.2996 |
| | Ca7-H17* | | 0.03885 | Ca(89.02%);H(10.98%) | Ca(s21 p63 d16);H(s100) | |
| | Ca7-H16 | 2.27566 | 1.78657 | Ca(11.28%);H(88.72%) | Ca(s21 p62 d17);H(s100) | 0.3086 |
| | Ca7-H16* | | 0.03998 | Ca(88.72%);H(11.28%) | Ca(s21 p62 d17);H(s100) | |
| 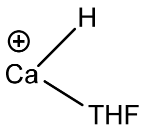  | Ca1-H2 | 2.08112 | 1.95931 | Ca(17.37%);H(82.63%) | Ca(s42 p39 d19);H(s100) | 0.5382 |
| | Ca1-H2* | | 0.02537 | Ca(82.63%);H(17.37%) | Ca(s42 p39 d19);H(s100) | |

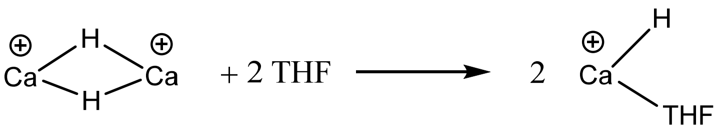


Δ*H* = -34.1 kcal/mol

Dissociation of the dimer by THF is highly favorable.

Molecular Orbitals：

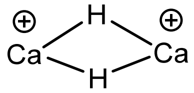


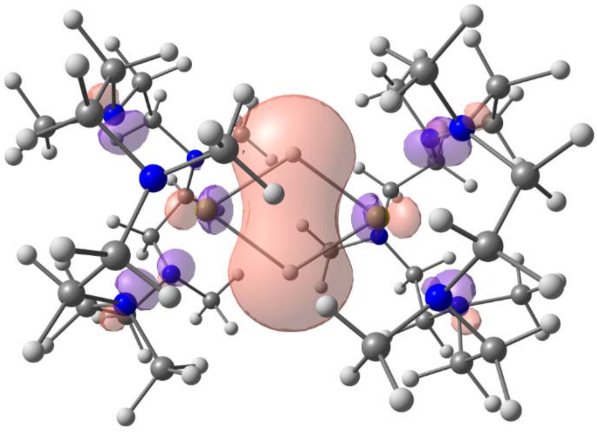

HOMO-1

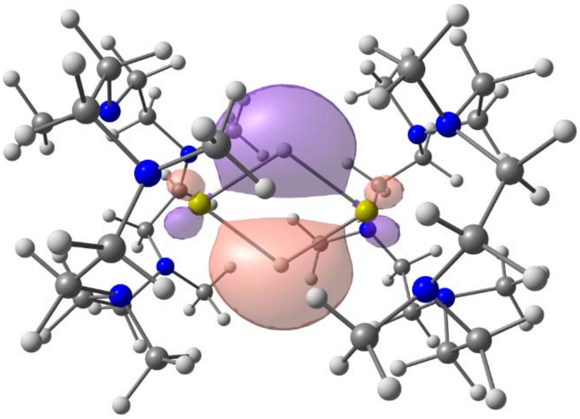

HOMO

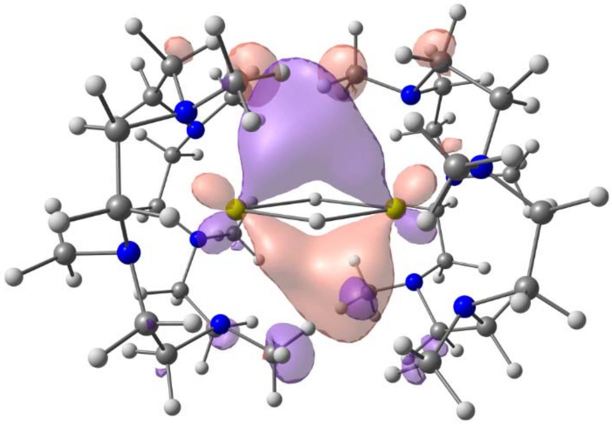

LUMO

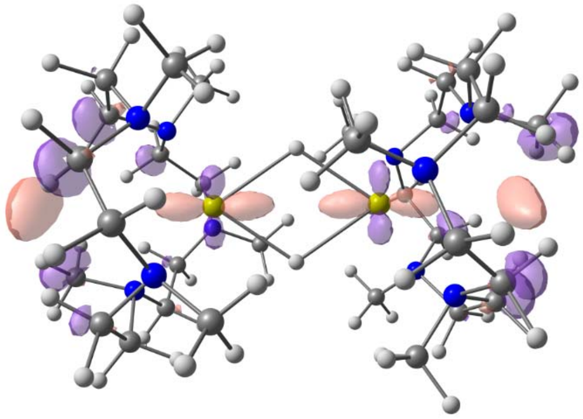

LUMO+2

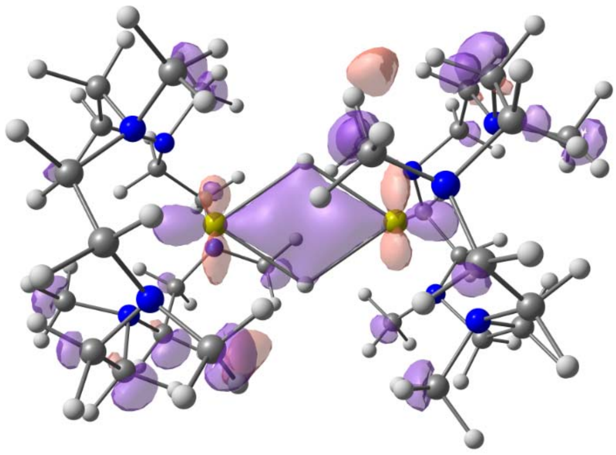

LUMO+4

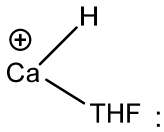


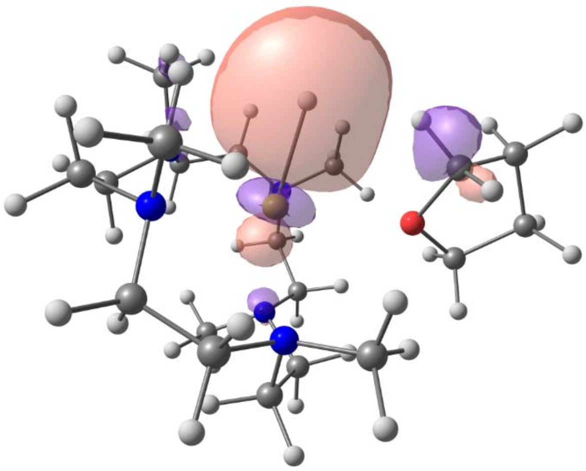

HOMO

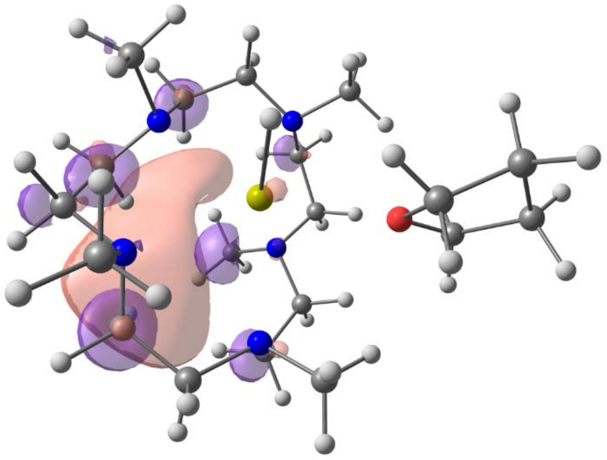

LUMO

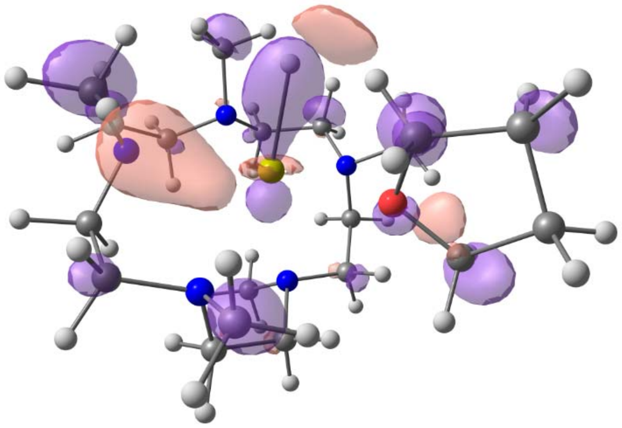

LUMO+3

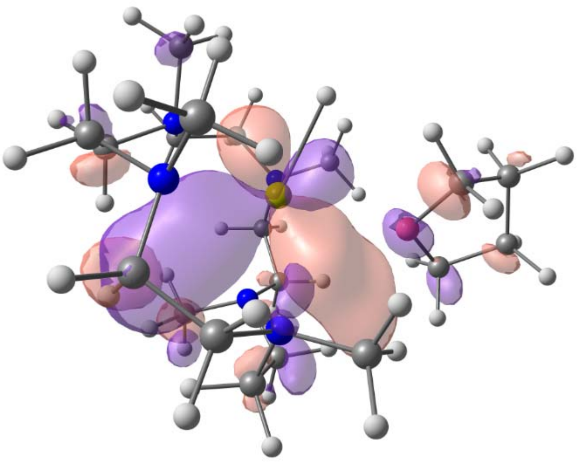

LUMO+4

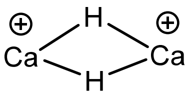

:

| | | | |
|---|---|---|---|
| C | 5.97968500 | 19.57377300 | 21.51426600 |
| H | 4.37181400 | 19.38591500 | 20.10495500 |
| N | 6.39764800 | 19.13530100 | 22.86068200 |
| H | 6.64005500 | 19.15051600 | 20.74077700 |
| H | 6.11093000 | 20.65744100 | 21.45286300 |
| C | 4.53791200 | 19.20724300 | 21.18089800 |
| Ca | 4.29015700 | 19.47343000 | 24.61238300 |
| C | 7.54849300 | 19.93414300 | 23.30966700 |
| C | 6.81053400 | 17.71597200 | 22.81380500 |
| N | 3.53482800 | 19.93660800 | 21.98404300 |
| H | 4.38283500 | 18.13397700 | 21.34083700 |
| Ca | 5.03906900 | 22.54309700 | 26.69307900 |
| N | 5.27986900 | 16.83851900 | 24.56713500 |
| N | 3.30422700 | 17.87970100 | 26.60526700 |
| N | 1.79689300 | 18.77163000 | 24.15543000 |
| H | 5.73897700 | 20.42287100 | 26.08823300 |
| H | 3.59023800 | 21.59364700 | 25.21724700 |
| H | 7.26604000 | 20.98517000 | 23.39010900 |
| H | 8.39924800 | 19.85197600 | 22.61502400 |
| H | 7.86796100 | 19.60741900 | 24.30086700 |
| C | 6.68431200 | 17.02041800 | 24.15696000 |
| H | 7.85100500 | 17.63572400 | 22.46155000 |
| H | 6.20473500 | 17.19697300 | 22.06725800 |
| C | 3.49123600 | 21.34845600 | 21.56925300 |
| C | 2.19006900 | 19.35526100 | 21.75277700 |
| N | 2.93156800 | 22.88127500 | 28.44476100 |
| N | 4.04939100 | 25.17802300 | 26.73827700 |
| N | 6.02503200 | 24.13679000 | 24.70018300 |
| N | 7.53233600 | 23.24488600 | 27.15004400 |
| N | 5.79437000 | 22.07994800 | 29.32144000 |
| C | 5.23711300 | 16.44736300 | 25.98833500 |
| C | 4.67052100 | 15.77823600 | 23.74931700 |
| C | 3.83378600 | 16.50148700 | 26.56742500 |
| C | 1.83553500 | 17.88759300 | 26.48650100 |
| C | 3.70994000 | 18.50340500 | 27.86679200 |
| C | 1.37001600 | 17.68049300 | 25.05262500 |
| C | 0.89066600 | 19.92123000 | 24.31711300 |
| C | 1.78974100 | 18.29022000 | 22.76013100 |
| H | 7.20898500 | 16.05130400 | 24.11331500 |
| H | 7.18759100 | 17.61536200 | 24.92699300 |
| H | 2.81357400 | 21.90063800 | 22.22419300 |
| H | 3.15271100 | 21.45886300 | 20.52729100 |
| H | 4.47588500 | 21.80713100 | 21.65118300 |
| H | 1.45791000 | 20.16568800 | 21.77873300 |
| H | 2.11968500 | 18.92640500 | 20.74198900 |
| C | 1.78070600 | 22.08246000 | 27.99577000 |
| C | 3.34951300 | 22.44281200 | 29.79118600 |
| C | 2.51870500 | 24.30061000 | 28.49162300 |
| C | 2.64494500 | 24.99614800 | 27.14846100 |
| C | 4.09214600 | 25.56915200 | 25.31707200 |
| C | 4.65876600 | 26.23831900 | 27.55605800 |
| C | 5.49547600 | 25.51500800 | 24.73799000 |
| C | 7.49372200 | 24.12889700 | 24.81897300 |
| C | 5.61935200 | 23.51304900 | 23.43866400 |
| C | 7.95922000 | 24.33601900 | 26.25285100 |
| C | 8.43856200 | 22.09528800 | 26.98834500 |

| | | | |
|---|---|---|---|
| C | 7.53948500 | 23.72630100 | 28.54534500 |
| C | 4.79128600 | 22.80933800 | 30.12456200 |
| C | 5.83792700 | 20.66810800 | 29.73625800 |
| C | 7.13913400 | 22.66128000 | 29.55271100 |
| H | 5.88487800 | 17.13615600 | 26.54284000 |
| H | 5.65072600 | 15.43485700 | 26.13233500 |
| H | 3.62482400 | 15.62742000 | 24.01940100 |
| H | 5.19538600 | 14.81769500 | 23.87405600 |
| H | 4.69370900 | 16.03796800 | 22.68971200 |
| H | 3.16007000 | 15.87136900 | 25.98257600 |
| H | 3.84402000 | 16.06445500 | 27.57786600 |
| H | 1.47571600 | 18.85372600 | 26.85452000 |
| H | 1.37612000 | 17.12233800 | 27.13312000 |
| H | 4.79229700 | 18.43181600 | 27.98263900 |
| H | 3.22702400 | 18.03133000 | 28.73793600 |
| H | 3.45688500 | 19.56593400 | 27.84847100 |
| H | 1.77766700 | 16.74171700 | 24.66419700 |
| H | 0.27447100 | 17.56462300 | 25.03807100 |
| H | 0.77721500 | 20.15507400 | 25.37628500 |
| H | -0.10690000 | 19.71239300 | 23.89939300 |
| H | 1.30554300 | 20.81356000 | 23.84678100 |
| H | 2.47090300 | 17.43302800 | 22.69735800 |
| H | 0.78995100 | 17.91853400 | 22.47689400 |
| H | 2.06313000 | 21.03142500 | 27.91533800 |
| H | 0.92994600 | 22.16465600 | 28.69040500 |
| H | 1.46125700 | 22.40918600 | 27.00456600 |
| H | 2.68914200 | 22.86608600 | 30.56466400 |
| H | 3.21826000 | 21.35914500 | 29.85260400 |
| H | 1.47823100 | 24.38087400 | 28.84386700 |
| H | 3.12450400 | 24.81960500 | 29.23817200 |
| H | 2.12028500 | 25.96527100 | 27.19209400 |
| H | 2.14165700 | 24.40120100 | 26.37843600 |
| H | 3.44437400 | 24.88035500 | 24.76258000 |
| H | 3.67854400 | 26.58166000 | 25.17305500 |
| H | 5.70445600 | 26.38912400 | 27.28593600 |
| H | 4.13390200 | 27.19886000 | 27.43131500 |
| H | 4.63561300 | 25.97861000 | 28.61566900 |
| H | 6.16918800 | 26.14513500 | 25.32283300 |
| H | 5.48525200 | 25.95201900 | 23.72754000 |
| H | 7.85354300 | 23.16275600 | 24.45097900 |
| H | 7.95314900 | 24.89414000 | 24.17234800 |
| H | 4.53699700 | 23.58462200 | 23.32278800 |
| H | 6.10228500 | 23.98510600 | 22.56752000 |
| H | 5.87241800 | 22.45052300 | 23.45701900 |
| H | 7.55156500 | 25.27479800 | 26.64126900 |
| H | 9.05476500 | 24.45188700 | 26.26741500 |
| H | 8.55201600 | 21.86146700 | 25.92916800 |
| H | 9.43612700 | 22.30411300 | 27.40607400 |
| H | 8.02368100 | 21.20294800 | 27.45865400 |
| H | 6.85833100 | 24.58350100 | 28.60810700 |
| H | 8.53927700 | 24.09798100 | 28.82858700 |
| H | 4.95737200 | 22.63067800 | 31.20051000 |
| H | 4.94637500 | 23.88260000 | 29.96460900 |
| H | 6.51557700 | 20.11589600 | 29.08133200 |
| H | 6.17644400 | 20.55771400 | 30.77822300 |
| H | 4.85326600 | 20.20945800 | 29.65433400 |
| H | 7.87128300 | 21.85084500 | 29.52677800 |
| H | 7.20951300 | 23.09015000 | 30.56349400 |

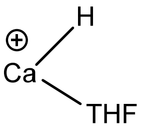

:

| | | | |
|---|---|---|---|
| Ca | 4.96165600 | 22.69332600 | 26.67700200 |
| H | 5.58405000 | 20.82280200 | 26.01005800 |
| N | 2.90753400 | 23.01507100 | 28.50781800 |
| N | 4.08801700 | 25.28333500 | 26.75491900 |
| N | 5.97134700 | 24.14078200 | 24.67268600 |
| N | 7.51586800 | 23.16087400 | 27.06662500 |
| N | 5.71100200 | 22.06178500 | 29.17130500 |
| C | 1.71970900 | 22.25169000 | 28.09456800 |
| C | 3.34313300 | 22.54410700 | 29.83761200 |
| C | 2.57567700 | 24.45228800 | 28.56671800 |
| C | 2.69510500 | 25.16014100 | 27.22382100 |
| C | 4.11048200 | 25.67310600 | 25.33169300 |
| C | 4.79147800 | 26.28948200 | 27.56046500 |
| C | 5.48616500 | 25.53317800 | 24.69198900 |
| C | 7.44321200 | 24.09447700 | 24.76567400 |
| C | 5.54079400 | 23.46119800 | 23.44042600 |
| C | 7.95168000 | 24.26052200 | 26.19256700 |
| C | 8.32109900 | 21.95824800 | 26.79586100 |
| C | 7.61615100 | 23.54700000 | 28.48185900 |
| C | 4.82323100 | 22.77120700 | 30.10736200 |
| C | 5.57130400 | 20.60129500 | 29.30889700 |
| C | 7.11604100 | 22.45811800 | 29.42312800 |
| H | 1.97779700 | 21.19579900 | 27.98128900 |
| H | 0.90355100 | 22.33785100 | 28.82928100 |
| H | 1.35844900 | 22.60654600 | 27.12911600 |
| H | 2.75798100 | 23.02575700 | 30.63735600 |
| H | 3.11935000 | 21.47697300 | 29.91058100 |
| H | 1.55162100 | 24.59247200 | 28.95001500 |
| H | 3.23397300 | 24.93234300 | 29.29597400 |
| H | 2.21949600 | 26.15302000 | 27.30017400 |
| H | 2.13216800 | 24.60198700 | 26.46896200 |
| H | 3.40054000 | 25.03111300 | 24.80039300 |
| H | 3.75682700 | 26.71035400 | 25.19972900 |
| H | 5.82522800 | 26.39780400 | 27.23027200 |
| H | 4.30633000 | 27.27694900 | 27.49240700 |
| H | 4.81892400 | 25.99689500 | 28.61171500 |
| H | 6.20865800 | 26.15298100 | 25.22971200 |
| H | 5.44278700 | 25.94587200 | 23.67170200 |
| H | 7.76730600 | 23.12786800 | 24.37182300 |
| H | 7.90520600 | 24.86016800 | 24.12141300 |
| H | 4.45996600 | 23.55391400 | 23.31977900 |
| H | 6.02407400 | 23.88727500 | 22.54723000 |
| H | 5.77895400 | 22.39625000 | 23.51045600 |
| H | 7.58098100 | 25.20199600 | 26.61431200 |
| H | 9.05129700 | 24.34609100 | 26.17471300 |
| H | 8.33839700 | 21.75128100 | 25.72678500 |
| H | 9.35626200 | 22.08286000 | 27.15458000 |
| H | 7.86687700 | 21.07902900 | 27.25026000 |
| H | 7.03004600 | 24.46415000 | 28.62549400 |
| H | 8.65685300 | 23.79091700 | 28.76078300 |
| H | 5.03058900 | 22.46822000 | 31.14894100 |
| H | 5.05598700 | 23.84200300 | 30.04636800 |
| H | 6.22962200 | 20.10657700 | 28.59345900 |
| H | 5.80305000 | 20.25907000 | 30.33012000 |
| H | 4.55829900 | 20.28283200 | 29.05657600 |
| H | 7.74988400 | 21.57440100 | 29.32296800 |
| H | 7.24707200 | 22.80210400 | 30.45967200 |

| | | | |
|---|---|---|---|
| C | 2.81753800 | 20.59146700 | 24.93739400 |
| O | 2.87980000 | 22.03209300 | 25.12224900 |
| C | 1.87112000 | 22.63803300 | 24.28806300 |
| C | 1.28514800 | 21.52692900 | 23.40585700 |
| C | 2.33536200 | 20.41754800 | 23.51115000 |
| H | 3.82056500 | 20.21325600 | 25.15848600 |
| H | 2.09392100 | 20.17058200 | 25.65116300 |
| H | 1.10427200 | 23.11238600 | 24.91486100 |
| H | 2.34917900 | 23.42051600 | 23.68717200 |
| H | 0.32844800 | 21.18176400 | 23.81066400 |
| H | 1.10905900 | 21.86544200 | 22.38179900 |
| H | 1.92450700 | 19.42243600 | 23.32406600 |
| H | 3.16025800 | 20.58507500 | 22.81046600 |

THF：

| | | | |
|---|---|---|---|
| C | 2.78367900 | 20.71735200 | 25.11737800 |
| O | 3.12962700 | 22.08737200 | 25.22243400 |
| C | 2.36504300 | 22.90189200 | 24.35086000 |
| C | 1.39148300 | 22.00331600 | 23.56602600 |
| C | 1.66512100 | 20.57174500 | 24.06890500 |
| H | 3.67795900 | 20.14461100 | 24.83499600 |
| H | 2.46170600 | 20.35479000 | 26.10344100 |
| H | 1.82879600 | 23.65405400 | 24.94590200 |
| H | 3.04630900 | 23.44196500 | 23.67881800 |
| H | 0.35373700 | 22.29611300 | 23.74748300 |
| H | 1.56221400 | 22.08522600 | 22.48913100 |
| H | 0.76831300 | 20.12796900 | 24.50974600 |
| H | 1.97612600 | 19.91647500 | 23.25073600 |

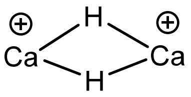


*Me4TACD:*

| | | | |
|---|---|---|---|
| Ca | -5.07305327 | 9.87477656 | 23.26276913 |
| Ca | -3.67791645 | 8.94948876 | 26.44832912 |
| N | -3.90545316 | 9.33337373 | 21.03420313 |
| N | -4.50352138 | 12.08526630 | 22.09306590 |
| N | -7.28436360 | 11.15404933 | 22.96784310 |
| N | -6.68448393 | 8.40210242 | 21.91270388 |
| N | -1.78621675 | 10.26192618 | 27.56930074 |
| N | -4.58941501 | 9.79240097 | 28.70580551 |
| N | -4.44975771 | 6.92784646 | 27.81328612 |
| N | -1.64105300 | 7.38922240 | 26.69223957 |
| C | -3.32318318 | 10.57638246 | 20.47942259 |
| H | -2.36117787 | 10.73955167 | 20.97520897 |
| H | -3.09743890 | 10.46416270 | 19.40737261 |
| C | -4.21351594 | 11.80021740 | 20.67236273 |
| H | -3.73170900 | 12.66602669 | 20.19075995 |
| H | -5.16134510 | 11.65377538 | 20.14684681 |
| C | -5.68933748 | 12.95904112 | 22.23374905 |
| H | -5.66738838 | 13.37655769 | 23.24486077 |
| H | -5.62897371 | 13.81775726 | 21.54608792 |
| C | -7.01638215 | 12.24368535 | 22.00198931 |
| H | -7.82467267 | 12.99146508 | 22.03516634 |
| H | -7.04044003 | 11.82289037 | 20.99252433 |
| C | -8.31462646 | 10.23044236 | 22.44086691 |
| H | -8.72416969 | 9.67451298 | 23.28979633 |
| H | -9.16005606 | 10.78655642 | 22.00651134 |
| C | -7.77655728 | 9.25466147 | 21.39847996 |
| H | -8.61168471 | 8.63896533 | 21.02809632 |
| H | -7.40593985 | 9.80943053 | 20.53170207 |

| | | | |
|---|---|---|---|
| C | -5.89796487 | 7.81316794 | 20.80626664 |
| H | -5.32851051 | 6.97653675 | 21.22198379 |
| H | -6.56064814 | 7.38220217 | 20.03888766 |
| C | -4.94885723 | 8.79825641 | 20.13195047 |
| H | -4.48718275 | 8.29990861 | 19.26465081 |
| H | -5.51670526 | 9.64006984 | 19.72639437 |
| C | -2.84044845 | 8.33256948 | 21.22266680 |
| H | -2.08329061 | 8.72403851 | 21.90782846 |
| H | -2.34799301 | 8.07423557 | 20.27334054 |
| H | -3.24685233 | 7.42119989 | 21.66566002 |
| C | -3.34732153 | 12.74242861 | 22.72786619 |
| H | -3.53850902 | 12.86148047 | 23.79686805 |
| H | -3.14301017 | 13.72770971 | 22.28285516 |
| H | -2.45035837 | 12.12847854 | 22.61542772 |
| C | -7.73797899 | 11.71841459 | 24.25143871 |
| H | -7.91055602 | 10.91169959 | 24.96947356 |
| H | -8.67203616 | 12.28902573 | 24.14114108 |
| H | -6.97361849 | 12.37569750 | 24.67058245 |
| C | -7.23155779 | 7.31481203 | 22.74372187 |
| H | -6.41012362 | 6.75359390 | 23.19564850 |
| H | -7.85988090 | 6.62837765 | 22.15668137 |
| H | -7.84259151 | 7.72269180 | 23.55274403 |
| C | -2.39614347 | 11.02719697 | 28.68036775 |
| H | -1.62197751 | 11.40834197 | 29.36519169 |
| H | -2.87758419 | 11.90737426 | 28.24367931 |
| C | -3.41489182 | 10.23245747 | 29.49115974 |
| H | -2.93647022 | 9.34851132 | 29.92293414 |
| H | -3.73459718 | 10.84906649 | 30.34641855 |
| C | -5.29167535 | 8.68447457 | 29.39103232 |
| H | -5.41124685 | 8.89434490 | 30.46559758 |
| H | -6.30475168 | 8.63234030 | 28.98049523 |
| C | -4.59928638 | 7.33514667 | 29.22632008 |
| H | -3.60583170 | 7.37234514 | 29.68208896 |
| H | -5.16390163 | 6.57773999 | 29.79300112 |
| C | -3.40848956 | 5.88645093 | 27.66497403 |
| H | -3.53708944 | 5.09268943 | 28.41780392 |
| H | -3.55881254 | 5.41159568 | 26.69086048 |
| C | -1.98393594 | 6.42381593 | 27.76015153 |
| H | -1.28741595 | 5.57035013 | 27.74489969 |
| H | -1.83691433 | 6.91259631 | 28.72762973 |
| C | -0.45926361 | 8.19406665 | 27.07367608 |
| H | 0.33654880 | 7.55783684 | 27.49197583 |
| H | -0.04704432 | 8.62861886 | 26.15754697 |
| C | -0.77972686 | 9.30312305 | 28.07103168 |
| H | -1.15323274 | 8.86500397 | 29.00085707 |
| H | 0.15573219 | 9.82160605 | 28.33496826 |
| C | -1.16763697 | 11.20333441 | 26.61948912 |
| H | -0.68812100 | 10.66050434 | 25.80086949 |
| H | -0.40417887 | 11.83045824 | 27.10340754 |
| H | -1.93915574 | 11.84729936 | 26.19008851 |
| C | -5.51911286 | 10.92041101 | 28.51699883 |
| H | -5.02718775 | 11.73557084 | 27.98329172 |
| H | -5.89893528 | 11.30213477 | 29.47644772 |
| H | -6.37023927 | 10.59987174 | 27.91033733 |
| C | -5.72697389 | 6.41137365 | 27.29057742 |
| H | -6.51785617 | 7.15639626 | 27.41005602 |
| H | -6.04245889 | 5.49530036 | 27.81163000 |
| H | -5.62473383 | 6.19809331 | 26.22351854 |
| C | -1.36010837 | 6.67021191 | 25.43651068 |
| H | -2.23130679 | 6.09166872 | 25.12382260 |
| H | -0.50123456 | 5.98999954 | 25.53807631 |

| | | | |
|---|---|---|---|
| H | -1.14172045 | 7.38769117 | 24.64113140 |
| H | -4.36647007 | 8.13787030 | 24.48232827 |
| H | -4.40459060 | 10.68273730 | 25.23780114 |

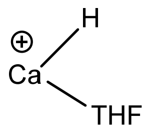


*Me4TACD:*

| | | | |
|---|---|---|---|
| Ca | -4.95204900 | 9.66258300 | 23.19859000 |
| N | -3.93868800 | 9.38493400 | 20.77868100 |
| N | -4.43396800 | 11.96065200 | 22.22279900 |
| N | -7.17911300 | 10.94515100 | 23.16090000 |
| N | -6.68524700 | 8.35766700 | 21.72345100 |
| C | -3.37788700 | 10.68710900 | 20.35486600 |
| H | -2.38325500 | 10.77619900 | 20.80216800 |
| H | -3.22535200 | 10.71377600 | 19.26353400 |
| C | -4.22990900 | 11.88432800 | 20.76767700 |
| H | -3.75103600 | 12.80066200 | 20.38458600 |
| H | -5.20785600 | 11.83025600 | 20.27955300 |
| C | -5.59157400 | 12.80742600 | 22.57612600 |
| H | -5.49436500 | 13.05447700 | 23.63671700 |
| H | -5.56302100 | 13.76267200 | 22.02650800 |
| C | -6.94424900 | 12.14419200 | 22.33038600 |
| H | -7.73451600 | 12.89243200 | 22.50695900 |
| H | -7.03095900 | 11.85821100 | 21.27718700 |
| C | -8.25342900 | 10.11031200 | 22.59154900 |
| H | -8.61593900 | 9.45154300 | 23.38686000 |
| H | -9.11914800 | 10.72035400 | 22.28574200 |
| C | -7.79632400 | 9.27487700 | 21.39989100 |
| H | -8.66057900 | 8.71546600 | 21.00559400 |
| H | -7.47257500 | 9.93732000 | 20.59214700 |
| C | -5.97497600 | 7.92724900 | 20.50183300 |
| H | -5.38537200 | 7.04246700 | 20.76140000 |
| H | -6.68773000 | 7.60257300 | 19.72576400 |
| C | -5.06326700 | 8.99506800 | 19.90423900 |
| H | -4.68606600 | 8.62652300 | 18.93594700 |
| H | -5.64736200 | 9.89108800 | 19.67660100 |
| C | -2.88189300 | 8.36239700 | 20.72370200 |
| H | -2.06831300 | 8.63180100 | 21.40275700 |
| H | -2.46419400 | 8.25547000 | 19.71058300 |
| H | -3.27261200 | 7.39130900 | 21.03588600 |
| C | -3.23172600 | 12.48580900 | 22.89598900 |
| H | -3.36492000 | 12.39897300 | 23.97706200 |
| H | -3.03604100 | 13.53259700 | 22.61683200 |
| H | -2.35389800 | 11.89276300 | 22.62708300 |
| C | -7.52302300 | 11.33918000 | 24.53984500 |
| H | -7.69520300 | 10.44508100 | 25.14435300 |
| H | -8.42832200 | 11.96535200 | 24.56915700 |
| H | -6.68905500 | 11.86990900 | 25.00087600 |
| C | -7.19193100 | 7.17082000 | 22.43069500 |
| H | -6.35354800 | 6.53516900 | 22.72533800 |
| H | -7.88197900 | 6.58246600 | 21.80628200 |
| H | -7.72595300 | 7.46480500 | 23.33779800 |
| H | -4.43664600 | 10.42435500 | 25.05999000 |
| C | -2.76086300 | 7.37279600 | 24.34342600 |
| O | -4.18939500 | 7.54433700 | 24.13955500 |
| C | -4.88852800 | 7.23217700 | 25.37684200 |
| C | -3.82120700 | 7.20855400 | 26.45681600 |
| C | -2.60781900 | 6.67981400 | 25.69048000 |
| H | -2.36473800 | 6.79359900 | 23.50454700 |

| | | | |
|---|---|---|---|
| H | -2.29498000 | 8.36706100 | 24.35927500 |
| H | -5.63963400 | 8.01074800 | 25.55062400 |
| H | -5.38250900 | 6.26042900 | 25.25528000 |
| H | -3.63950900 | 8.22791700 | 26.81115100 |
| H | -4.10369300 | 6.58105900 | 27.30559400 |
| H | -1.65678300 | 6.92359100 | 26.16993600 |
| H | -2.66251000 | 5.59218600 | 25.57402000 |

# 6 References


[S1] a) V. Leich, T. P. Spaniol, L. Maron, J. Okuda, *Angew. Chem. Int. Ed.* **2016**, *55*, 4794-4797; b) A. Causero, G. Ballmann, J. Pahl, H. Zijlstra, C. Farber, S. Harder, *Organometallics* **2016**, *35*, 3350-3360; c) A. S. S. Wilson, M. S. Hill, M. F. Mahon, *Organometallics* **2018**, *38*, 351-360. d) M. S. Hill, M. F. Mahon, A. S. S. Wilson, C. Dinoi, L. Maron, E. Richards, *Chem. Commun.* **2019**, *55*, 5732-5735.

[S2] a) S. Harder, S. Müller, E. Hübner, *Organometallics* **2004**, *23*, 178-183; b) B. M. Wolf, C. Maichle-Mössmer, R. Anwander, *Angew. Chem. Int. Ed.* **2016**, *55*, 13893-13897.

[S3] J. H. Coates, D. A. Hadi, S. F. Lincoln, *Aust. J. Chem.* **1982**, *35*, 903.

[S4] R. M. Fabicon, M. Parvez, H. G. Richey, *Organometallics* **1999**, *18*, 5163-5169.

[S5] D. Schuhknecht, C. Lhotzky, T. P. Spaniol, L. Maron, J. Okuda, *Angew. Chem. Int. Ed.* **2017**, *56*, 12367-12371.

[S6] a) J. Burés, *Angew. Chem. Int. Ed.* **2016**, *55*, 16084-16087; b) J. Burés, *Angew. Chem. Int. Ed.* **2016**, *55*, 2028-2031; c) C. D. T. Nielsen, J. Burés, *Chemical Science* **2019**, *10*, 348-353.

[S7] G.M. Sheldrick, *Acta Crystallogr. A* **2015**, 71, 3-8.

[S8] G.M. Sheldrick, *Acta Crystallogr. C* **2015**, 71, 3-8.

[S9] O. V. Dolomanov, L. J. Bourhis, R. J. Gildea, J. A. K. Howard, H. Puschmann, *J. Appl. Crystallogr.* **2009**, *42*, 339-341.

[S10] K. B. H. Putz, *Diamond - Crystal and Molecular Structure Visualization, Crystal Impact,* Bonn, **2017**.

[S11] a) J. P. Perdew, Y. Wang, *Phys. Rev. B* **1992**, *45*, 13244-13249. b) A. D. Becke, *J. Chem. Phys.* **1993**, *98*, 5648-5652.

[S12] M. J. Frisch, G. W. Trucks, H. B. Schlegel, G. E. Scuseria, M. A. Robb, J. R. Cheeseman, V. G. Zakrzewski, J. A. Montgomery, R. E. Stratman, J. C. Burant, S. Dapprich, J. M. Millam, A. D. Daniels, K. N. Kudin, M. C. Strain, O. Farkas, J. Tomasi, V. Barone, M. Cossi, R. Cammi, B. Mennucci, C. Pomelli, C. Adamo, S. Clifford, J. Ochterski, G. A. Petersson, P. Y. Ayala, Q. Cui, K. Morokuma, D. K. Malick, A. D. Rabuck, K. Raghavachari, J. B. Foresman, J. Cioslowswi, J. V. Ortiz, A. G. Baboul, B. B. Stefanov, G. Liu, A. Liashenko, P. Piskorz, I. Komaromi, R. Gomperts, R. Martin, D. J. Fox, T. Keith, M. A. AlLaham, C. Y. Peng, A. Nanayakkara, C. Gonzalez, M. Challacombe, P. M. W. Gill, B. Jonhson, W. Chen, M. W. J. Wong, L. Andres, M. Head-Gordon, E. S. Replogle, J. A. Pople, Gaussian 09; D.01 ed. Pittsburgh PA **2009**.

[S13] J.-P. Blaudeau, M. P. McGrath, L. A. Curtiss, L. Radom, *J. Chem. Phys.* **1997**, *107*, 5016-5021.

[S14] A. E. Reed, L. A. Curtiss, F. Weinhold, *Chem. Rev.* **1988**, *88*, 899-926.
